# Decriminalizing same-sex relationships and its impact on social media discussions in Asia

**DOI:** 10.1038/s41562-026-02485-6

**Published:** 2026-06-10

**Authors:** Kokil Jaidka, Preetika Verma, Yiting Chen

**Affiliations:** 1 Department of Communications and New Media, National University of Singapore, Singapore, Singapore; 2 School of Computer Science, Carnegie Mellon University, Pittsburgh, PA USA; 3 School of Journalism and Mass Communication, University of Wisconsin Madison, Madison, WI USA

**Keywords:** Human behaviour, Politics and international relations

## Abstract

While legal reforms may coincide with shifts in public discourse, broader normative change often remains uneven and context dependent. Since 2018, four Asian countries—India, Taiwan, Thailand and Singapore—have decriminalized same-sex relationships, providing an opportunity to examine how such institutional changes are associated with public discussions of LGBTQ+ topics. Here, using supervised and unsupervised natural language processing methods, we analysed 136,255 Facebook news posts and 65,969 public Instagram captions within a 100-day window before and after each reform. To contextualize online discourse with offline sentiment, we incorporated nationally representative survey data from the Gallup World Poll (*N* = 46,200). Following decriminalization, online discussions were characterized by reduced moralized and affectively negative language, particularly in contexts with greater freedom of expression. By contrast, offline perceptions of neighbourhood safety for same-sex couples shifted more modestly and unevenly across age groups and countries.

## Abstract (web summary)

Legal reforms decriminalizing same-sex relations in Asia led to modest normative changes, reflected in reduced online moral judgement and improved offline perceptions of neighbourhood safety, particularly among younger populations.

## Main

Nearly 35% of countries continue to criminalize same-sex sexual activity^1^; however, under increasing international pressure, many have taken steps towards greater inclusion of same-sex relationships^2^. For instance, in 2018, India repealed the 377A law and legalized consensual same-sex intercourse between adults^3^. Four years later, Singapore repealed a similar 377A law^4^. Meanwhile, Taiwan legalized same-sex marriage in 2019^5^. In 2022, Thailand announced a future date after which same-sex marriages would be legalized^6^. However, the normative acceptance of same-sex relationships goes beyond legislative action and is intertwined with social and government infrastructural support. Especially in conservative societies, individuals who violate societal ‘moral contracts’ often face hate speech^7–9^.

To further explain the dynamics between legislative change and social attitudes, Structuration Theory^10^ offers a valuable lens, as it posits how social practices are shaped by both individual agency (the actions of individuals) and structures (institutional frameworks). Legislative changes, such as the decriminalization of LGBTQ+ identities, can act as catalysts to changes in social norms and attitudes. When governments formally recognize LGBTQ+ rights, they help reinforce societal values of inclusion, which can then influence public discourse, both offline and online. Therefore, the actions of institutions (such as the decriminalization of same-sex relationships) and individual expressions on social media are interconnected, with both contributing to shifts in public opinion and acceptance of LGBTQ+ individuals. For instance, a lack of representation in government policies may reflect in how LGBTQ+ people are underrepresented or vilified in the media and online or portrayed through stereotypes^11,12^. This may also explain why individuals were more likely to express their opinions and provide their rationale on LGBTQ+ issues when news emphasizes LGBTQ+ issues^13^.

Most studies on public opinion towards LGBTQ+ people rely on national-level surveys, which may not capture online realities and are often affected by government censorship and social desirability biases, particularly in conservative societies. Yet, the debate over whether online spaces are more inclusive for marginalized groups, such as LGBTQ+ individuals, or more vilifying compared with offline realities remains unresolved. Few opportunities exist to explore how external changes, such as the lifting of laws criminalizing same-sex relationships, shape public discourse and media attention to LGBTQ+ issues. This motivates our first research question:

**RQ1**: What are the effects of decriminalizing same-sex relationships on (a) news coverage and (b) social media attention to LGBTQ+ topics?

Empirical findings present a mixed picture of whether social media helps LGBTQ+ individuals bridge identity differences with broader society^14^. On the one hand, some research highlights social media’s role in promoting acceptance and advancing LGBTQ+ rights^15,16^, while on the other, studies suggest that platform design and anonymity can reinforce social norms that restrict open expression, particularly for queer individuals^17–19^. In conservative societies, individuals who violate dominant ‘moral contracts’ often face hate speech and backlash^7–9^. For instance, a UK survey found that over 60% of LGBTQ+ respondents experienced online harassment^20^, and such exposure has been linked to psychological distress and social distrust^21–24^.

Beyond measuring incidence, however, we lack a fine-grained understanding of how users morally reason about LGBTQ+ topics across different cultural and platform contexts. Prior research shows that moral language often structures public discourse^25^, and that moral values shape how news events and marginalized groups are framed^26–29^. LGBTQ+ issues are particularly likely to trigger moral intuitions that influence stereotypes, judgements and public evaluations of who deserves moral consideration^30^.

To examine these dynamics, we use Moral Foundations Theory (MFT) as a measurement framework. MFT organizes moral reasoning into two key clusters: individualizing foundations (care/harm and fairness/cheating), which emphasize justice and protection of individuals; and binding foundations (loyalty/betrayal, authority/subversion and sanctity/degradation), which emphasize group cohesion, tradition and purity^31^. These foundations provide a structured way to measure how moral stereotypes manifest in discourse about LGBTQ+ identities, particularly in response to major institutional shifts.

While MFT has been widely applied to political ideology and moral framing, few studies have leveraged it to systematically analyse public discourse about LGBTQ+ issues across multiple social media platforms and national contexts. Recent work has shown that moralized language is a key component of online toxicity^32^, highlighting the need for a framework that can detect shifts in moral reasoning over time. Given the dual legal and moral nature of decriminalization, our next research question asks:

**RQ2**: How does the legal acceptance of same-sex relationships affect (a) affective negativity and (b) moral stereotypes in LGBTQ+ social media discourse?

Structuration theory would also explain endogenous confounds, if any. In societies with higher levels of social equality and stronger institutional support for civil liberties, structures that promote equality and protect individual rights tend to be more robust and deeply embedded in the social fabric. Structuration theory would therefore lead one to expect that, in societies with higher levels of social equality and institutional support for civil liberties, legislative changes around decriminalizing LGBTQ+ minorities may lead to a reconfiguration of moral attitudes. In this way, examining the interactive effect of democratic indicators can help us identify how the impact of legislative changes differs depending on the institutional and social environment. This motivates our final research question:

**RQ3**: To what extent do country-level institutional indicators moderate online and offline responses to legislative change?

Our analysis draws on three datasets. The Facebook dataset comprises 136,255 multilingual posts from national news outlets in India, Taiwan, Thailand and Singapore (2016–2023; 86.87% in English). The Instagram dataset comprises 7.6 million public posts from the same countries and period (74.37% in English). The Gallup dataset comprises 46,200 survey responses from the Gallup World Poll (2017–2022), in which respondents were asked whether they perceived their neighbourhood as safe for same-sex couples. Triangulating social media discourse with nationally representative survey data allows us to compare online and offline attitudes, and to assess whether our findings generalize beyond the Instagram demographic^33^.

To answer RQ1 and RQ2, we applied interrupted time series (ITS) analyses to examine post-legislative shifts in toxicity, moral stereotypes and engagement across the Facebook and Instagram datasets, and in perceived neighbourhood safety in the Gallup dataset. To answer RQ3, we added interaction terms between the post-legislative indicator and institutional or demographic moderators to assess how country-level context shapes the direction and magnitude of these shifts.

Our study makes both theoretical and methodological contributions. Theoretically, it contextualizes critical social issues by linking news agendas and public opinion to underlying social and structural mechanisms. Methodologically, we introduce (1) an unsupervised neural network-based approach to filter relevant social media data, and (2) a measure of daily attention to LGBTQ+ topics, operationalized as the proportion of a user’s or outlet’s overall activity. These methods allow us to assess how legislative change shapes everyday engagement on social media.

## Results

### Variables of interest

**Fig. 1:**
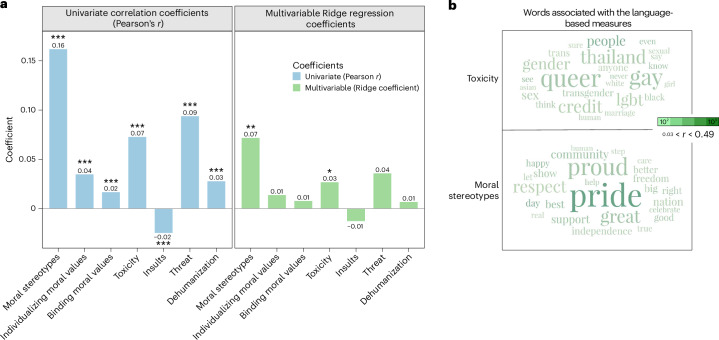
Language features associated with LGBTQ+ discourse on Instagram. **a**, Univariate Pearson correlations (*r*) and multivariable ridge regression coefficients for language-based measurements predicting whether a post concerns LGBTQ+ topics. Left: univariate correlations between each linguistic feature and LGBTQ+ topic classification. Right: coefficients from a multivariable ridge regression including all features simultaneously. Univariate associations are uniformly significant, whereas multivariable estimates reveal that only a subset of features retain independent predictive power. Detailed coefficients are reported in Supplementary Table 10. **b**, Words most strongly correlated with the toxicity and moral stereotype measures shown in **a**. Word size denotes the magnitude of its correlation with LGBTQ+ topic classification (*P* < 0.001, corrected for multiple comparisons). Shade indicates normalized frequency, with darker shades reflecting higher relative frequency. Detailed coefficients are reported in Supplementary Table 11. Source data

To identify which language features most strongly predict whether a post is about LGBTQ+ topics, we conducted an exploratory analysis using Pearson correlation and ridge regression, following standard methods in computational linguistics^34–36^. Figure 1a reports coefficients from these analyses, showing how language-based features such as moral stereotypes^37^, individualizing and binding moral values^38^, hate speech^39^, threats^40^ and dehumanization^41^ are associated with the likelihood that a post concerns LGBTQ+ topics (Supplementary Table 10).

Moral stereotypes (*β* = 0.072, 95% confidence interval (CI) 0.023 to 0.122, *P* = 0.004) and toxicity (*β* = 0.027, 95% CI 0.004 to 0.050, *P* = 0.021), derived from the Perspective API^39^, emerged as strong positive predictors, absorbing 3.7% of the variance from related features. These findings support the use of these two measures as core indicators of moral and affective content in our analysis. Figure 1b further unpacks these coefficients by visualizing the specific words most strongly associated with high toxicity and moral stereotype scores.

However, the word-level correlations for the toxicity score raise concerns because they suggest that posts mentioning the words ‘queer’ (*r* = 0.316, 95% CI 0.309 to 0.324, *P* < 0.001) or ‘gay’ (*r* = 0.313, 95% CI 0.305 to 0.321, *P* < 0.001) would be more likely to be flagged as toxic. This reflects a known limitation of supervised hate speech classifiers, which often conflate sexual identity terms with harmful content^42^. While we mitigate this issue by restricting our dataset to LGBTQ+ discourse and using toxicity as a relative (rather than absolute) affective signal, it underscores the need for caution when interpreting results derived from such models.

By contrast, the moral stereotypes measure, based on a curated lexicon, shows stronger alignment with LGBTQ+ advocacy language. Words like ‘pride’ (*r* = 0.345, 95% CI 0.338 to 0.352, *P* < 0.001), ‘proud,’ (*r* = 0.225, 95% CI 0.218 to 0.232, *P* < 0.001) and ‘community’ (*r* = 0.127, 95% CI 0.120 to 0.135, *P* < 0.001) are associated with higher scores, reinforcing its theoretical relevance. All word-level correlations are reported in Supplementary Table 11. The transparency of the lexicon-based approach also allows us to manually audit and adapt it for greater validity in this context.

Taken together, these diagnostic analyses demonstrate both the utility and the limitations of using off-the-shelf affective and moral language measures. While they help capture shifts in the public discourse around LGBTQ+ issues, their outputs must be interpreted with an awareness of potential bias, especially when the models themselves may reflect societal prejudice.

### Changes in attention

**Fig. 2:**
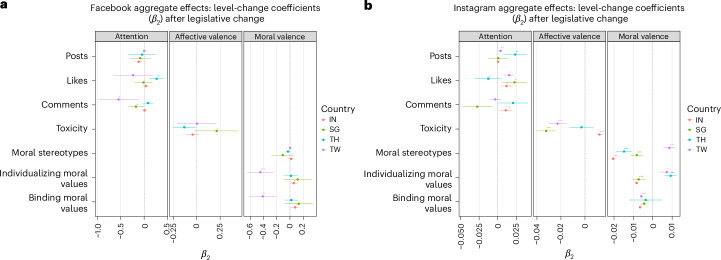
Effects of legislative change on attention, affective valence and moral valence of LGBTQ+ discourse. **a**,**b**, OLS estimates of the effect of the legislative change on the attention to and valence of posts by news outlets on Facebook (**a**) and Instagram posts by individual accounts (**b**) (bandwidth 100 days). The whiskers report 95% CIs. In **a**, evidence is from the Facebook dataset, and in **b**, evidence is from the Instagram dataset (***P ≤ 0.001, **P ≤ 0.01, *P ≤ 0.05, ^+^*P* ≤ 0.1). The full results for the Instagram dataset are reported in Supplementary Tables 12–14. IN, India; SG, Singapore; TH, Thailand; TW, Taiwan. Source data

Figure 2a,b (Supplementary Tables 12 and 15) demonstrates the impact of legislative changes on various characteristics of news articles and public discourse, respectively, focusing on attention, affective valence and moral valence measures, where *β*_2_ denotes the coefficient on the post-period indicator and *β*_3_ the slope change. Across both platforms, attention effects were mixed and country-specific, with no consistent direction emerging across the four countries. For the Facebook dataset, post volume declined only in India (*β*_2_ = −0.115, standard error (SE) 0.036, 95% CI −0.185 to −0.045, *P* = 0.002), with no statistically significant change in Singapore, Thailand or Taiwan. Comment volume fell significantly in Taiwan (*β*_2_ = −0.545, SE 0.218, 95% CI −0.971 to −0.118, *P* = 0.027), while remaining unchanged in Singapore (*β*_2_ = −0.174, SE 0.087, 95% CI −0.344 to −0.004, *P* = 0.050), India (*β*_2_ = 0.008, SE 0.022, *P* = 0.730) and Thailand (*β*_2_ = 0.082, SE 0.060, *P* = 0.175). Likes increased only in Thailand (*β*_2_ = 0.267, SE 0.084, 95% CI 0.102 to 0.432, *P* = 0.002), with no statistically significant change elsewhere. These findings are reported in Supplementary Tables 12–14.

For the Instagram dataset, post volume followed a similarly mixed pattern, increasing significantly in Thailand (*β*_2_ = 0.024, SE 0.008, 95% CI 0.008 to 0.040, *P* = 0.004) and Taiwan (*β*_2_ = 0.004, SE 0.001, 95% CI 0.001 to 0.006, *P* = 0.002), but remaining unchanged in India (*β*_2_ = 3.07 × 10^−4^, SE 6.07 × 10^−4^, 95% CI −8.84 × 10^−4^ to 0.001, *P* = 0.614) and Singapore (*β*_2_ = 7.91 × 10^−4^, SE 0.007, 95% CI −0.013 to 0.014, *P* = 0.908). Likes broadly increased following the legislative change: significant increases were observed in India (*β*_2_ = 0.012, SE 0.004, 95% CI 0.004 to 0.019, *P* = 0.049), Singapore (*β*_2_ = 0.023, SE 0.009, 95% CI 0.006 to 0.039, *P* = 0.020) and Taiwan (*β*_2_ = 0.015, SE 0.004, 95% CI 0.009 to 0.022, *P* = 0.033), with no statistically significant change in Thailand (*β*_2_ = −0.013, SE 0.009, 95% CI −0.031 to 0.006, *P* = 0.071). Comment activity increased only in India (*β*_2_ = 0.011, SE 0.005, 95% CI 0.002 to 0.020, *P* < 0.001), with no statistically significant change in Singapore (*β*_2_ = −0.027, SE 0.010, *P* = 0.190), Thailand (*β*_2_ = 0.021, SE 0.010, *P* = 0.246) or Taiwan (*β*_2_ = −0.003, SE 0.004, *P* = 0.100).

### Changes in affective and moral valence

Toxicity patterns (Fig. 2a,b and Supplementary Tables 13 and 16) showed no substantive effects on Facebook but varied markedly by country on Instagram. On Facebook, toxicity declined only in Thailand (*β*_2_ = −0.134, SE 0.062, 95% CI −0.256 to −0.012, *P* = 0.036); no statistically significant change was observed in India (*β*_2_ = −0.045, SE 0.038, *P* = 0.247), Singapore (*β*_2_ = 0.213, SE 0.120, *P* = 0.081) or Taiwan (*β*_2_ = 0.003, SE 0.106, *P* = 0.979).

For the Instagram dataset, on the one hand, significant immediate declines were observed in Singapore (*β*_2_ = −0.032, SE 0.004, 95% CI −0.040 to −0.024, *P* < 0.001) and Taiwan (*β*_2_ = −0.023, SE 0.004, 95% CI −0.030 to −0.015, *P* < 0.001), while on the other hand, toxicity increased significantly in India (*β*_2_ = 0.012, SE 0.001, 95% CI 0.010 to 0.015, *P* < 0.001). No statistically significant immediate change was observed in Thailand (*β*_2_ = −0.003, SE 0.005, 95% CI −0.013 to 0.007, *P* = 0.501). Slope change estimates (Supplementary Table 16) suggest these effects continued to develop over time: the decline in Taiwan intensified (*β*_3_ = −4.94 × 10^−4^, SE 6.78 × 10^−5^, 95% CI −6.27 × 10^−4^ to −3.62 × 10^−4^, *P* < 0.001), the increase in India continued (*β*_3_ = 0.002, SE 2.49 × 10^−5^, 95% CI 0.002 to 0.002, *P* < 0.001), and a negative trend in toxicity emerged in Thailand despite no statistically significant immediate effect (*β*_3_ = −0.001, SE 8.84 × 10^−5^, 95% CI −0.001 to −9.36 × 10^−4^, *P* < 0.001). The slope change in Singapore was not statistically significant (*β*_3_ = 1.51 × 10^−4^, SE 6.95 × 10^−5^, 95% CI 1.44 × 10^−5^ to 2.87 × 10^−4^, *P* = 0.096).

Moral language outcomes (Fig. 2a,b and Supplementary Tables 14 and 17) showed clearer cross-country differentiation. On Facebook, no statistically significant changes in moral stereotyping were observed in any country (all *P* > 0.10). Binding and individualizing moral discourse showed a divergent pattern between India and Taiwan: binding moral discourse increased in India (*β*_2_ = 0.077, SE 0.027, 95% CI 0.024 to 0.131, *P* = 0.008) while decreasing in Taiwan (*β*_2_ = −0.411, SE 0.104, 95% CI −0.615 to −0.208, *P* = 0.002), with no statistically significant change in Singapore (*P* = 0.224) or Thailand (*P* = 0.685). Similarly, individualizing moral discourse decreased significantly in Taiwan (*β*_2_ = −0.448, SE 0.101, 95% CI −0.647 to −0.249, *P* = 0.001), while no statistically significant change was observed in India (*β*_2_ = 0.055, SE 0.028, 95% CI 7.50 × 10^−3^ to 0.110, *P* = 0.063), Singapore or Thailand (both *P* > 0.29).

By contrast, Instagram showed clearer and more consistent cross-country patterns, where moral stereotyping declined in three of the four countries: India (*β*_2_ = −0.020, SE 5.82 × 10^−4^, 95% CI −0.022 to −0.019, *P* < 0.001), Thailand (*β*_2_ = −0.015, SE 0.002, 95% CI −0.019 to −0.011, *P* < 0.001) and Singapore (*β*_2_ = −0.008, SE 0.002, 95% CI −0.011 to −0.005, *P* < 0.001). Taiwan showed a significant increase (*β*_2_ = 0.009, SE 0.002, 95% CI 0.006 to 0.012, *P* < .001). Binding moral discourse declined broadly, with significant decreases in Singapore (*β*_2_ = −0.004, SE 5.49 × 10^−4^, 95% CI −0.006 to −0.003, *P* < 0.001) and Taiwan (*β*_2_ = −0.006, SE 2.13 × 10^−4^, 95% CI −0.006 to −0.005, *P* < .001), no statistically significant change in India (*β*_2_ = − 0.007, SE 3.25 × 10^−4^, 95% CI −0.007 to −0.006, *P* = 0.051) and no statistically significant change in Thailand (*β*_2_ = −0.004, SE 0.004, 95% CI −0.012 to 0.005, *P* = 0.437). Individualizing moral discourse showed a divergence: declines in India (*β*_2_ = −0.008, SE 4.59 × 10^−4^, 95% CI −0.009 to −0.007, *P* = 0.037) and Singapore (*β*_2_ = −0.007, SE 0.002, 95% CI −0.010 to −0.004, *P* < 0.001) contrasted with increases in Thailand (*β*_2_ = 0.009, SE 0.002, 95% CI 0.006 to 0.012, *P* = 0.003) and Taiwan (*β*_2_ = 0.007, SE 0.002, 95% CI 0.003 to 0.011, *P* = 0.035).

Slope change estimates (Supplementary Table 17) indicate that most of these effects continued to develop over time. For moral stereotyping, negative trends persisted in India (*β*_3_ = −4.66 × 10^−4^, SE 1.14 × 10^−5^, 95% CI −4.89 × 10^−4^ to −4.44 × 10^−4^, *P* < 0.001), Thailand (*β*_3_ = −6.92 × 10^−4^, SE 3.70 × 10^−5^, 95% CI −7.65 × 10^−4^ to −6.19 × 10^−4^, *P* < 0.001), and Taiwan (*β*_3_ = −5.34 × 10^−4^, SE 2.69 × 10^−5^, 95% CI −5.87 × 10^−4^ to −4.81 × 10^−4^, *P* < 0.001), while a positive slope change in Singapore suggests a return towards pre-intervention levels (*β*_3_ = 2.04 × 10^−4^, SE 2.76 × 10^−5^, 95% CI 1.50 × 10^−4^ to 2.58 × 10^−4^, *P* < 0.001). For individualizing moral discourse, negative trends continued in India (*β*_3_ = −2.95 × 10^−4^, SE 9.02 × 10^−6^, 95% CI −3.13 × 10^−4^ to −2.77 × 10^−4^, *P* < 0.001) and Thailand (*β*_3 _= −2.43 × 10^−4^, SE 2.84 × 10^−5^, 95% CI −2.99 × 10^−4^ to −1.88 × 10^−4^, *P* < 0.001); slope changes were not significant in Singapore (*β*_3_ = 1.71 × 10^−6^, SE 2.82 × 10^−5^, 95% CI −5.36 × 10^−5^ to 5.70 × 10^−5^, *P* = 0.306) or Taiwan (*β*_3_ = −6.02 × 10^−5^, SE 3.55 × 10^−5^, 95% CI −1.30 × 10^−4^ to 9.40 × 10^−6^, *P* = 0.151). For binding moral discourse, negative trends continued in India (*β*_3_ = −1.31 × 10^−4^, SE 6.39 × 10^−6^, 95% CI −1.43 × 10^−4^ to −1.18 × 10^−4^, *P* < 0.001), Singapore (*β*_3_ = −5.36 × 10^−5^, SE 9.45 × 10^−6^, 95% CI −7.21 × 10^−5^ to −3.50 × 10^−5^, *P* < 0.001) and Taiwan (*β*_3_ = −7.96 × 10^−5^, SE 3.78 × 10^−6^, 95% CI −8.70 × 10^−5^ to −7.21 × 10^−5^, *P* < 0.001), while a positive slope change in Thailand indicates the initial effect diminished over time (*β*_3_ = 4.73 × 10^−4^, SE 7.73 × 10^−5^, 95% CI 3.21 × 10^−4^ to 6.24 × 10^−4^, *P* < 0.001).

### Contextual variation in observed responses

To further characterize the heterogeneity observed across countries in the preceding analyses, and to address RQ3, we examined whether country-level institutional indicators—media censorship, freedom of expression, gender equality and social group equality—were associated with variation in post-legislative patterns of toxic discourse. We estimated models that included interaction terms between the post-legislative indicator and each contextual variable (defined in the ‘Contextual variation in observed responses’ section in Methods).

**Fig. 3:**
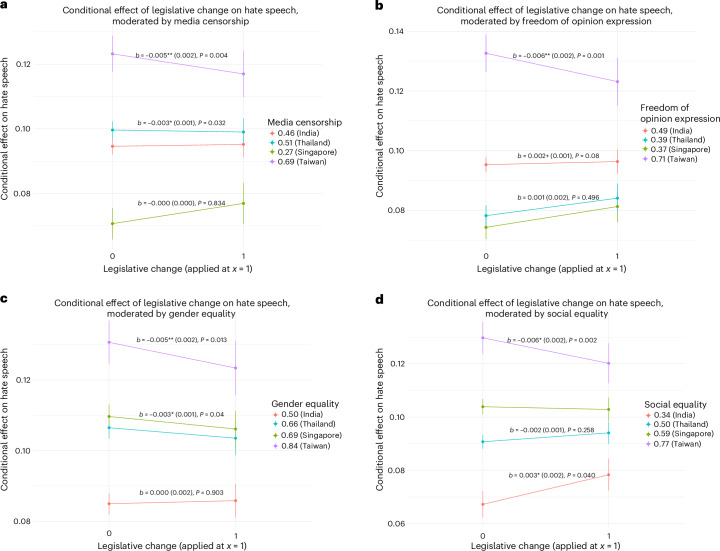
Institutional moderators of the effect of legislative change on online hate speech. **a**–**d**, The estimated marginal effects of legislative change on the prevalence of hate speech on Instagram, conditioned by: media censorship (**a**), freedom of opinion expression (**b**), gender equality (**c**) and social equality (**d**). Values of each moderator correspond to country-level scores: India, Thailand, Singapore and Taiwan. The *y* axis reflects the conditional effect of the legislative change variable (0 = pre-change, 1 = post-change), derived from OLS models with interaction terms. The data comprise the Instagram dataset. The unit of analysis is the daily measures of hate speech. Each line represents one country; points represent model-predicted marginal means, and vertical lines indicate 95% CIs. Data were grouped based on country-specific legislative changes. The full set of coefficients are reported in Supplementary Tables 15–17. Source data

Figure 3 shows that the main effect of legislative change on hate speech was negligible and non-significant (*b* = 0.001, SE 0.010, 95% CI –0.019 to 0.021), indicating no average shift in toxicity when institutional context is not taken into account. This null average effect, however, masked heterogeneity across institutional contexts: all four interaction terms were statistically significant (0.039 ≤ *b* ≤ 0.068, 0.006 ≤ SE ≤ 0.012, *P* < 0.001; Supplementary Table 18), indicating that the association between legislative change and toxicity varied with the institutional environment. In these models, the main effect of legislative change when institutional indicators approach their minimum is negative (Supplementary Table 18: −0.042 ≤ *b*_after _≤ −0.025, all *P* < 0.001), and the positive interaction terms indicate that this effect is progressively attenuated as institutional freedom and equality increase. Predicted toxicity increased significantly under the gender equality model (Δ = 0.011, 95% CI 0.003 to 0.019) and the freedom of expression model (Δ = 0.007, 95% CI 0.000 to 0.014).

### Triangulation with survey data

Table 1 reports results from a regression analysis examining how legislative change relates to public perceptions of neighbourhood safety for same-sex couples, based on annual Gallup surveys. We constructed a nationally representative daily indicator of perceived LGBTQ+ safety using Gallup’s survey weights. To address data sparsity during the COVID-19 lockdown period, we applied a minimum bandwidth of 230 days to ensure model stability and convergence.

**Table 1:** Evidence from the Gallup dataset: main effects of legislative change on attitudes towards same-sex couples

	Estimate	SE	*p*	95% CI	Sig.
(Intercept)	13.143	4.881	0.008	3.577 to 22.709	**
Legislative change	−11.523	4.881	0.019	−21.089 to −1.957	*
Number of days	0.051	0.022	0.020	0.008 to 0.094	*
Legislative change × Days	−0.052	0.022	0.019	−0.094 to −0.009	*
Observations	180				
Marginal *R*^2^	0.041				
Conditional *R*^2^	0.133				

Coefficients are marginal effects; standard errors, *P* values (3 decimal places) and 95% CIs are reported. (***P ≤ 0.001; **P ≤ 0.01; *P ≤ 0.05; ^+^*P* ≤ 0.1).

The marginal effects of legislative change on attitudes towards same-sex couples are as follows. The coefficient for legislative change is negative and statistically significant (*β* = −11.523, SE 4.881, 95% CI −21.089 to −1.957, *P* = 0.019), indicating that reported perceptions of neighbourhood safety for gay men and lesbians declined following the reform, net of temporal trends and other controls. The negative interaction between legislative change and time (*β* = −0.052, SE 0.022, 95% CI −0.094 to −0.009, *P* = 0.019) suggests that this decline modestly intensified over the observed period rather than attenuating.

#### Contextual variation in self-reports

We also examined whether differences between patterns observed in the Instagram and Gallup datasets correspond to demographic variation, particularly the younger and more digitally engaged populations typically represented on Instagram.

Age was associated with heterogeneity in reported neighbourhood safety following the legislative change. In India and Singapore, younger Gallup respondents were more likely to report perceiving their neighbourhoods as safe for same-sex couples, whereas in Thailand, older respondents were less likely to report such perceptions.

**Fig. 4:**
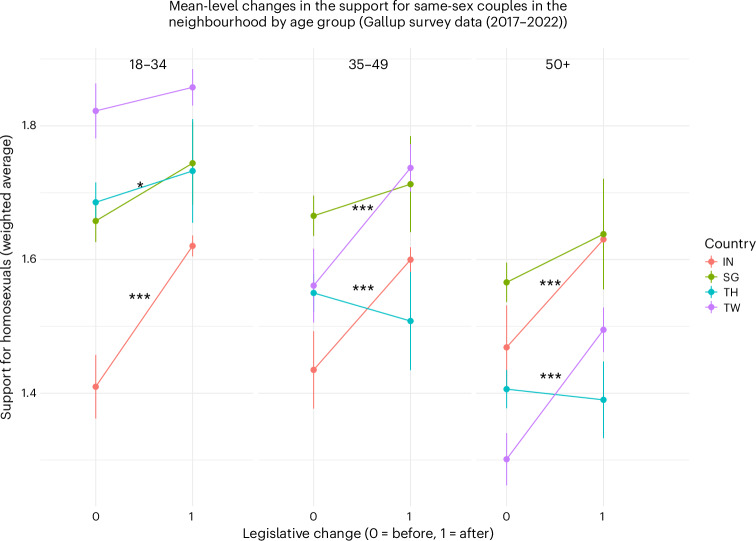
Age-group differences in public support for same-sex couples before and after legislative change (Gallup World Poll, 2017–2022). The figure shows mean-level changes in perceived neighbourhood support for same-sex couples across three age groups (18–34, 35–49 and 50+) in India (IN), Singapore (SG), Thailand (TH) and Taiwan (TW), based on *N* = 46,200 nationally representative survey responses. Legislative change is coded as a binary variable (0 = before, 1 = after). Points represent weighted daily mean values, and vertical lines denote 95% CIs. Reported differences are based on independent-sample two-tailed *t*-tests comparing means before and after legislative change within each age group and country, adjusted for multiple comparisons. The full set of results are reported in Supplementary Tables 19 and 20. ***P ≤ 0.001, **P ≤ 0.01, *P ≤ 0.05, ^+^*P* ≤ 0.1). Source data

Figure 4 illustrates these patterns in greater detail. Cross-column comparisons highlight demographic variation in reported perceptions, while intercolumn comparisons illustrate cross-national differences within the same age cohort. Marked within-country variation was observed in Taiwan, and notable between-country differences were found among respondents aged 18–34. Following the legislative change, reported perceptions of neighbourhood support for same-sex couples increased across all age groups in India (*b* = 0.21 for ages 18–34, *b* = 0.17 for ages 35–49, *b* = 0.16 for ages 50+; SE 0.008–0.010, all *P* < 0.001), among respondents aged 35–49 and 50+ in Taiwan (*b* = 0.18 and 0.19, respectively; SE 0.017–0.018, both *P* < 0.001), and among respondents under 35 in Singapore (*b* = 0.09, SE 0.032, *P* = 0.019). Detailed estimates are reported in Supplementary Tables 19 and 20.

### Additional robustness checks

#### Controlling for account-level heterogeneity in the Instagram dataset

To assess whether observed changes reflect within-user behavioural shifts rather than compositional differences across accounts, that is, whether effects arise because existing users changed their behaviour or because different users entered the conversation, we re-estimated all models using mixed-effects models with random intercepts for account (lmer). These models are reported in Supplementary Information appendix C.7. Results were most consistent for moral language outcomes: declines in binding moral discourse replicated within accounts across all four countries, in some cases exceeding the aggregate estimates, and moral stereotype results were consistent in direction for Thailand and Taiwan. By contrast, engagement and toxicity outcomes showed greater divergence. Toxicity reversed direction in India (aggregate: *β* = 0.012, 95% CI 0.010 to 0.015, *P* < 0.001; within-user: *β* = −0.019, 95% CI −0.024 to −0.014, *P* < 0.001) and Taiwan (aggregate: *β* = −0.023, 95% CI −0.030 to −0.015, *P* < 0.001; within-user: *β* = 0.019, 95% CI 0.015 to 0.023, *P* < 0.001), and likes reversed in Singapore and Thailand, indicating that these aggregate patterns partly reflect compositional shifts in who entered the conversation following the legislative change. Taken together, the within-user results suggest that shifts in moral framing, particularly binding moral language, reflect genuine behavioural change among existing users, while engagement and toxicity outcomes are more sensitive to audience composition.

#### Placebo Instagram datasets

We also replicated our analysis with four placebo datasets collected from the unfiltered Instagram dataset that comprised hashtags related to food, travel, photography or fashion. The analyses did not yield statistically significant associations for these placebo datasets, as expected, providing evidence that the observed shifts in LGBTQ+ discourse are not attributable to general temporal trends in unrelated topics. While these null results support the specificity of the findings, we do not interpret them as definitive evidence of no change in unrelated discourse. Rather, they lend additional confidence that our main results are unlikely to be artefacts of broader platform dynamics. These results are reported in Supplementary Information appendix C.7.3.

## Discussion

This study examined whether the decriminalization of same-sex relationships predicts shifts in online discourse across countries and platforms. Drawing on structuration theory^10^, we conceptualize legal reforms as moments that may reconfigure the institutional conditions under which social practices unfold. Our ITS and mixed-effects analyses show that legislative change systematically predicts post-intervention variation in discourse, although the magnitude and direction of these shifts differ across sociopolitical contexts.

Our results demonstrate that institutional reforms predict measurable changes in online discourse, but not in uniform ways. Legal recognition is associated with statistically significant reductions in moral stereotyping and affective toxicity in several contexts, while in others the effects are weaker or absent. These heterogeneous effects are consistent with structuration theory, which posits that institutional transformation reshapes the field of possible action but does not eliminate pre-existing structural constraints. In our models, countries with stronger institutional safeguards exhibit more pronounced reductions in moral polarization and toxic language, whereas more constrained environments show more limited or adverse discursive shifts. These findings suggest that institutional context conditions how legal reform translates into observable changes in public discourse. When legislative change reflects or reinforces prevailing social norms and institutional protections, it may reduce the salience or perceived legitimacy of hostile discourse; where it diverges from dominant social attitudes, it may instead activate defensive responses, producing measurable upticks in toxic and morally polarized content.

We further find that legislative reform predicts declines in moral polarization and affective toxicity in Instagram discourse. Posts became significantly less likely to invoke hate speech in Singapore and Taiwan; in Thailand, no significant immediate change was observed but a significant negative slope change emerged over time (*β*_3_ = −0.001, *P* < 0.001). Posts also became significantly less likely to rely on moral stereotyping in India, Singapore and Thailand. Extending prior work on social media and LGBTQ+ identity formation^15,16^, our findings indicate that legal reform is a meaningful predictor of how LGBTQ+ topics are morally framed online. Online discourse appears responsive to institutional change, although the strength of this response varies with structural protections such as press freedom and civil liberties.

At the same time, these affective shifts did not coincide with increased visibility or participation. Across both Instagram and Facebook, legislative reform predicts mixed attention effects, with declines observed in most country–platform combinations but notable exceptions, including a significant increase in commenting activity in India on Instagram. This attenuation of engagement may reflect issue-attention cycles following major policy milestones^43^, ongoing concerns about surveillance and exposure^18,44,45^, or platform-specific demographic and moderation dynamics^46^. For example, Facebook’s comparatively older user base, typically less supportive of LGBTQ+ rights^47^, may partially account for more muted engagement patterns.

In sum, these findings suggest that legislative reform functions as a meaningful institutional predictor of discursive change, but its effects are mediated by broader structural conditions. Consistent with structuration theory, legal reforms alter the institutional parameters within which discourse unfolds, generating statistically detectable shifts in framing and affect. However, the translation of institutional change into durable transformations in public discourse depends on contextual enablers such as civic norms, platform governance and protections for expressive participation.

## Conclusion

Prior accounts suggest that LGBTQ+ individuals remain highly visible yet unevenly protected in digital spaces, where expressions of solidarity, stigma and silence often coexist^48–52^. While the repeal of Section 377A marked an important legal milestone, it does not on its own guarantee broader social inclusion. Our findings show that legal reform predicts statistically significant shifts in online discourse, although the magnitude and direction of these shifts vary across institutional contexts and reflect pre-existing inequalities in media environments and civic protections.

These patterns highlight that legal reform, while symbolically powerful, does not automatically produce inclusive public discourse. Instead, its effects depend on how legal change interacts with platform governance, cultural norms and broader political conditions. This observation aligns with research emphasizing that visibility and participation online are unevenly distributed and shaped by structural constraints^18,45^.

For policymakers, our findings underscore the importance of pairing legal reform with complementary institutional supports, including protections for free expression, safeguards against harassment, and inclusive media governance. For platforms, they point to the need for design and moderation practices that account for uneven vulnerability and differential exposure. For researchers, the results highlight the importance of situating digital trace data within broader sociopolitical contexts and of interpreting post-reform shifts as empirically identifiable changes in discourse rather than purely descriptive fluctuations.

Overall, our study suggests that legal change reconfigures the institutional terrain within which online discourse unfolds and predicts measurable transformations in framing and affect, without deterministically fixing their direction. Understanding how institutional reforms interact with platform infrastructures and social norms remains essential for assessing the broader societal consequences of legal change and for supporting more equitable digital publics.

### Limitations

To address issues of sample representativeness, we have triangulated our findings with survey data. We have also conducted multiple unsupervised and semi-supervised passes to filter our dataset for relevance. For the Facebook and Instagram datasets, while our analysis focused on Instagram and Facebook posts and engagement on LGBTQ+ discourse about the four countries studied, the responses we analysed may include contributions from users outside these regions. The CrowdTangle API does not provide location-specific social media posts; as such, our findings reflect global discourse on local LGBTQ+ issues, which may incorporate perspectives beyond the target countries. This may also be appropriate, given the transnational nature of online discussions on LGBTQ+ topics and rights, and suggests that future research could explore ways to better isolate region-specific public opinion in similar contexts. We also recognize that, in focusing on LGBTQ+ discourse more broadly, we lose the nuance between stances towards LGBTQ+ people and LGBTQ+ self-expression, which we plan to study in the future to better understand individual cognitive and emotional appraisals after the legislative change, such as through self-disclosures of emotional well-being^35^, stress^53^, locus of control^54^ and loneliness^55^.

A related limitation concerns code-mixed language. In Southeast Asia, multilingual communication is common, and social media users often blend English with Mandarin, Tamil, Bahasa Indonesia, Thai and other regional languages within a single post. Such intrasentential code-mixing may reduce the precision of translation and automated classification systems not explicitly trained on these patterns. Although we validated our measures through human annotation and robustness checks, future work could improve accuracy by fine-tuning models on Southeast Asian code-mixed corpora ^56^.

For the Gallup dataset, we offer the public perception of attitudes towards same-sex couples as the closest approximation of our measures for social media data and hope that future survey panels will consider a broader repertoire of questions about attitudes towards marginalized communities. Although a single-item measure is not ideal, and a third-person perception of environmental safety differs from a direct report of personal attitudes towards LGBTQ+ people, it is the only available daily trend, as the other major indicator, the Social Acceptance Index of LGBTI people, is reported as 4-year averages across 178 counties^57^. Furthermore, a perception-based variable in face-to-face surveys can minimize the risk of biased responses in questions of a sensitive nature. It may also offer a reasonable proxy for personal opinions, as prior work on self- and perceived attitudes of peers towards lesbians and gay men suggest that people perceive others’ attitudes on the basis of their own predispositions^58^. Analyses based on an online panel of 856 American adults suggested that their media consumption and interpersonal contact predict their attitudes towards same-sex couples, and they presume similar but attenuated effects when they consider the attitudes of their close friends or their peer group. Our research, which focuses on daily averages, appropriately interprets the data as macrolevel shifts in societal attitudes. Yet, including account information as fixed effects in these models suggest that for India and Taiwan, as well as for Thailand to some extent, the findings can also be interpreted as the shift in how the LGBTQ+ discourse was being framed at the individual level.

## Methods

The study protocol was determined as exempted from a full review by the Institutional Review Board of the National University of Singapore (NUS-IRB-LS-20-097E and NUS-IRB-2025-839).

### Ethics and inclusion statement

The research questions addressed in the study aim to illuminate the role of policy infrastructure in protecting minority communities online. Although care has been taken to ensure that the insights do not perpetuate the harmful profiling of LGBTQ+ communities, some of the example social media posts reported in Supplementary Table 5 may be deemed offensive by readers. The examples included in this Article are not verbatim quotes but paraphrased representations, rewritten for anonymity and ethical compliance. Our analyses clarify and evaluate the possible artefacts in the data wherever relevant, such as its demographic representativeness. Additional steps have been taken to ensure the responsible release of the data. Robustness checks and triangulated methods address the generalizability of the findings and the reproducibility of the results.

#### Data

##### Primary dataset: newsfeeds on Facebook

Facebook posts from over 50 major news outlets in India, Taiwan, Thailand and Singapore were collected using the CrowdTangle API’s media outlet lists. The research time window was from 2016 to 2023, spanning at least 2 years before and up to 2 years after the legislative change in each country. CrowdTangle is an insights tool from Meta that offers API access to content on public Facebook pages. The fraction of social media users who use social media for news ranges from 58% to 91% (58% for Singapore and Taiwan, 84% for India and 91% for Thailand)^6^. Therefore, the Facebook feed of news outlets comprises a high-reach medium to reach citizens in South/Southeast Asia. Therefore, measures of attention, affect and morality based on this feed offer a valid media agenda estimate.

The dataset comprised 136,255 posts, with most posts in 4 languages. In total, 86.87% of posts were in English, 5.19% of posts were in Thai, 2.01% of posts were in Chinese and 1.96% of posts were in Hindi. Detailed information regarding the distribution of posts from each country is included in Supplementary Table 1.

##### Secondary dataset: Instagram captions

The secondary dataset was sourced from Instagram captions via the CrowdTangle API, encompassing public posts about the same four countries during the identical timeframe. Given Instagram’s status as Asia’s second-most popular platform^59^ and its significance for LGBTQ+ individuals’ self-expression^60,61^, text analysis of Instagram posts is integral to this study. Furthermore, even and especially when the visual content of Instagram posts may be abstract, picturing inanimate objects, landscapes or everyday experiences^62^, analysing the captions ensures that we can contextualize the content based on the users’ intention through the self-declared hashtags. In our case, we narrowed our focus to the global LGBTQ+ discourse about a country. While this study aims to analyse social media discourse surrounding LGBTQ+ legislation in India, Taiwan, Thailand and Singapore, the CrowdTangle API did not allow location-based searches of social media posts. Nevertheless, users often rely on hashtags or contextual tags to indicate the focus or relevance of their posts, such as using tags related to specific countries, cities or events. Accordingly, without a way to restrict the CrowdTangle API to geocoded posts, we curated country-specific datasets through searching based on country keywords, such as India, Taiwan, Thailand and Singapore, and then further filtering the dataset to retain only those posts that are relevant to LGBTQ+ topics. Other studies have adopted a similar approach for curating online datasets relevant to a geographic location by using location-specific keywords in the search query, for example, for curating location-specific Google News results^63–65^.

As a result of this choice, the data used in this study probably include contributions from users both within and outside these countries. This means that the discourse captured on social media may reflect a broader, global conversation around LGBTQ+ issues in each country, rather than being strictly limited to local public opinion. The full list of keywords is provided in Supplementary Table 3. Ultimately, the Instagram dataset comprised 65,969 posts. In total, 74.37% of the posts were in English, 6.19% in Chinese and 3.04% in Thai.

##### Tertiary dataset: survey data from the Gallup World Poll

Daily individual-level perceptions of attitudes towards LGBTQ+ people and rights were drawn from the Gallup World Poll ^66^, a cross-national, repeated cross-sectional survey administered to nationally representative samples using standardized questionnaires and translation protocols. We analyse responses from four countries—India, Singapore, Taiwan and Thailand—collected between 2017 and 2022 (total *N* = 46,200). Across countries, samples were broadly gender-balanced (for example, 45−61% women), with age distributions spanning young adulthood through older age (18–65+). Sample demographics are reported in Supplementary Table 7. To make respondents’ levels of education comparable across countries, Gallup classifies the educational attainment into three categories: 1 = elementary education or up to 8 years of basic education; 2 = secondary education or 9–15 years of education; and 3 = tertiary education or 4 years of education beyond ‘high school’ or received a 4-year college degree (mean (*M*) = 1.84, s.d. 0.68).

#### Measures from the Facebook and Instagram datasets

A description of the data preprocessing steps, including machine translation and filtering, are reported in Supplementary Information appendix A. To measure the change in social media discussions about LGBTQ+ topics, a morally loaded issue, we measured the message-level affective valence and the moral valence of the social media posts during the period of interest.

For the Facebook and Instagram datasets in each country, we calculated day-user- or day-news outlet-level averages (hereafter collectively referred to as day-account-level averages) for measures of attention and valence based on the translated posts. The means, medians and standard deviations of these scores are reported in Supplementary Table 9. To tackle the inconsistency of the number of posts each day and avoid type I errors, we sampled the approximate daily mean of 100 posts per user per day for each analysis. All variables were scaled to values between 0 and 1 to interpret the interaction term easily. Finally, we constructed time series as daily account-level 3-day rolling averages. The individual measures of valence were calculated using state-of-the-art neural network models, defined in Table 2 and reported below.

**Table 2:** The measures derived from the Facebook and Instagram datasets

Dimension	Operationalization	Source
Attention		
Posts	The average proportion of daily posts about LGBTQ+ topics	CrowdTangle API
Likes	The average proportion of daily likes on posts about LGTBQ+ topics	CrowdTangle API
Comments	The average proportion of daily comments on posts about LGTBQ+ topics	CrowdTangle API
Affective valence		
Toxicity	Whether a post contains abuses, slurs, identity attacks and so on (0 to 1 continuous values).	Perspective API
Moral valence		
Moral stereotypes	Language that emphasizes moral traits in discussions of LGBTQ+ topics	(Nicolas, Bai & Fiske, 2020)^37^
Individualizing moral foundations: care	The tendency to avoid harming others and to help those in need. It involves promoting well-being, alleviating suffering and protecting the vulnerable (0 to 1 continuous values)	(Hopp, 2021)^38^
Fairness	The tendency to value and enforce fairness, justice and impartiality, and avoid cheating and exploitation (0 to 1 continuous values)	(Hopp, 2021)^38^
Binding moral foundations: loyalty	The tendency to value and protect one’s group or community (0 to 1 continuous values)	(Hopp, 2021)^38^
Authority	The tendency to respect and follow established rules and leaders, and value order and tradition (0 to 1 continuous scale)	(Hopp, 2021)^38^

For attention, the CrowdTangle API reports the aggregate numbers of interactions (for example, the numbers of likes and comments). We computed daily Attention measures for LGBTQ+ topics by determining the proportion of posts, comments and likes relative to their overall number for each account. Other studies have also used similar concepts to operationalize topical attention in the news^67^.

For affective valence, we calculated the toxicity score as the average of six dimensions reported by the Perspective API, namely toxicity, insult, profanity, identity attack, threat and sexual explicitness. The Perspective API has been applied and validated in many recent studies to measure online hate speech in different domains, such as online conversations, political comments and product reviews^68–72^.

For moral valence, we applied the single-dimension Moral Stereotypes Dictionary^37^ to quantify the use of moral language. The moral foundations framework^73^ categorizes moral motivations into two dimensions: individualizing and binding values. Therefore, we also applied the extended five-dimension Moral Foundation Dictionary (eMFD)^38^ to calculate the individualizing and binding moral value scores for LGBTQ+ discourse. We also evaluated a machine learning approach for measuring moral values that is discussed in Supplementary Information appendix C.7.2.

Individualizing moral values reflect the pursuit of compassionate and fair treatment of individuals and consist of the care/harm and fairness/cheating values^31^. Care refers to mentions that show empathy to vulnerable others, while fairness discusses justice, equality and reciprocity^74^. Binding moral values connect people to well-defined roles in groups and communities. They include mentions of loyalty, authority and sanctity^30^. The message-level scores of care and fairness were averaged to obtain a score for individualizing moral values. Similarly, the average score of authority and loyalty was used as the binding moral value scores (individualizing moral values *M* = 0.048, s.d. 0.14; binding moral values *M* = 0.045, s.d. 0.012 for the Instagram datasets). Sanctity was not included in the operationalization owing to its low validity against human annotations, as reported in Supplementary Table 6.

#### Measures from the Gallup dataset

To measure LGBTQ+ attitudes, respondents in each country were asked whether they perceive their community as safe for lesbians and gay men (1 = yes, 0 = no), considered the primary dependent variable. Gallup also provides response-level weights, which we used to construct a daily weighted average of public opinion towards same-sex couples in each country.

#### Contextual variation in observed responses

We next examined how observed responses vary across demographic and institutional contexts. Demographic information was available only for the Gallup data; therefore, we included age as a moderator in the Gallup dataset to examine whether age groups influence the effect of legislative changes on overall negative attitudes towards same-sex couples in the neighbourhood. The age variable was divided into three terciles: 18–34, 35–49 and 50 and above, based on recent reports that at least over two-thirds of Instagram’s user base is under 35 (ref. ^47^). Using the 50 and above age group as the reference category, we included an interaction term in the primary effect model to examine its effect on the shift in discourse after the legislative change.

For the Instagram and Facebook datasets, we expect that country-level social and legislative norms around media freedom, freedom of expression, and social equality may affect public responses to legislative change. Accordingly, country-level democratic indicators from the Global State of Democracy Indices (GSoD)^75^ and the Varieties of Democracy (V-Dem) dataset were included as contextual moderators. All indicators are scaled 0–1, where higher values indicate greater freedom or equality. The four moderators are described below:

- Media freedom: The extent of government print and broadcast censorship effort, where higher scores indicate less government censorship (that is, more media freedom). Country averages: Taiwan 0.73, India 0.47, Singapore 0.36, Thailand 0.24.
- Freedom of expression: A composite index of freedom of expression, where higher scores indicate greater freedom. Country averages: Taiwan 0.74, India 0.49, Singapore 0.42, Thailand 0.36.
- Gender equality: A composite measure of gender-based disparities, where higher scores indicate greater equality. Country averages: Taiwan 0.82, Singapore 0.68, Thailand 0.62, India 0.48.
- Social group equality in civil liberties: The extent to which all social groups enjoy equal civil liberties, where higher scores indicate greater equality. Country averages: Taiwan 0.75, Singapore 0.50, India 0.49, Thailand 0.34.

#### ITS analysis

As a preliminary step, we conducted exploratory analyses to identify linguistic features most strongly associated with LGBTQ+ discourse. Specifically, we estimated pairwise Pearson correlations and ridge regression models on a combined dataset of LGBTQ+-related and placebo (non-LGBTQ+) posts, as described in Supplementary Information appendix B.6. Ridge regression models were estimated using the regressors package (v0.0.3), with regularization parameters ranging from 10^−5^ to 2 and all predictors rescaled to the [0, 1] interval.

Our primary analyses (RQ1–RQ3) use an ITS design, which is well suited for evaluating discrete interventions occurring at known timepoints in high-frequency observational data. The ITS framework enables estimation of both immediate level changes and post-intervention trend changes following legislative reform.

The core model is specified as

1 $${Y}_{\mathrm{feature},t}={\beta }_{0}+{\beta }_{1}T+{\beta }_{2}{X}_{t}+{\beta }_{3}(T\times {X}_{t}),$$

where *T* denotes time relative to the legislative change (with *T* = 0 at the intervention), and *X*_*t*_ is a binary indicator equal to 1 for observations after the intervention and 0 otherwise. The coefficient *β*_2_ captures the immediate level change following the reform, while *β*_3_ captures changes in the post-intervention slope relative to the pre-intervention trend. Consistent with prior work using ITS designs ^76–80^, our primary inferential focus is on the level change, with slope estimates interpreted as secondary dynamic adjustments.

#### Analytical procedure

Analyses were conducted separately by country to account for contextual heterogeneity in media systems and political discourse. As a robustness check, we additionally estimated specifications with account-level fixed effects to control for time-invariant differences across users; these results are reported in Supplementary Fig. 1. For the main analyses, we use a symmetric window of 100 days before and after the legislative change. For the Gallup survey data, which are observed at lower temporal resolution, a wider window of 230 days was used.

##### Model diagnostics and assumption checks

ITS models rely on assumptions regarding temporal dependence, variance structure and functional form. Because social media data are inherently noisy, bursty and shaped by platform dynamics, these assumptions cannot be taken for granted and must be empirically evaluated.

We assessed serial dependence using Durbin–Watson and Breusch–Godfrey tests (seven lags), and evaluated heteroskedasticity using the Breusch–Pagan test. Across outcomes related to attention and engagement (for example, comments, likes and views), diagnostics indicated strong temporal dependence and frequent heteroskedasticity, consistent with prior work on high-frequency online behaviour. By contrast, moral and affective outcomes exhibited more heterogeneous patterns: some outcomes showed mild serial dependence or variance instability, while others showed little evidence of assumption violations. In addition, although the distribution of residuals departs from normality in several specifications, as is typical for high-frequency behavioural data, the large sample sizes (for example, *N* = 136,255 Facebook posts; *N* = 65,969 Instagram posts) ensure that coefficient estimates remain asymptotically normal. Under these conditions, inference based on robust standard errors remains valid due to the central limit theorem, even when residual distributions are skewed or heavy-tailed.

To account for these patterns, all models were estimated using heteroskedasticity- and autocorrelation-consistent (Newey–West) standard errors. In addition, we examined influence diagnostics using Cook’s distance; across all outcomes and countries, values remained well below conventional thresholds, indicating that results were not driven by isolated observations.

Finally, to assess robustness to distributional assumptions and local irregularities, we conducted bootstrap resampling of daily observations. Estimates were highly stable across resamples, indicating that the substantive conclusions are not sensitive to model specification or sampling variability. Taken together, these diagnostics suggest that, while social media time series routinely violate classical ordinary least squares (OLS) assumptions, the combination of heteroskedasticity- and autocorrelation-consistent (HAC) inference and resampling provides reliable estimation of intervention effects in this setting.

#### Estimation and inference

We report average standardized coefficients and standard errors based on 100 bootstrap iterations, each drawing approximately 100 daily observations per user with replacement. For the Facebook and Instagram datasets, analyses focus on the 100-day window before and after the legislative change; for the Gallup dataset, the wider temporal spacing required a 230-day window. All hypothesis tests are two-sided. Statistical analyses were conducted in R using the lmerTest package (v3.1.3) and the interactions package (v1.2.0), and in Python using the regressors package (v0.0.3).

To evaluate specificity, we additionally conducted placebo analyses using non-LGBTQ-related Instagram posts. As reported in Supplementary Information appendix B.6, these placebo models yielded no systematic effects, supporting the interpretation that observed changes are specific to LGBTQ+ discourse rather than reflecting platform-wide temporal dynamics.

#### Post-hoc power and sensitivity

Post-hoc power analyses indicate that the Instagram sample (*N* = 65,969) provides approximately 95% power to detect small effects (Cohen’s *d* ≈ 0.05), while the Gallup sample (*N* = 46,200) provides approximately 80% power to detect effects of *d* = 0.15. Effect sizes and CIs are reported throughout, and uncertainty is visualized where relevant (Fig. 2).

### Reporting summary

Further information on research design is available in the Nature Portfolio Reporting Summary linked to this article.

## Supplementary information

Supplementary Information Supplementary Figs. 1 and 2, Methodological Details and Tables 1–30.

Reporting Summary

Peer Review File

## Source data

Source Data Fig. 1 Source data.

Source Data Fig. 2 Source data.

Source Data Fig. 3 Source data.

Source Data Fig. 4 Source data.

Source Data Extended Data Table 2 Source data.

## Data Availability

The Facebook and Instagram datasets were collected using Meta’s Content Library (formerly CrowdTangle), in accordance with Meta’s platform policies and data access requirements. These data are not redistributed by the authors. Readers may apply for access to Meta data via the Meta Content Library and Researcher Platform at https://transparency.meta.com/researchtools/meta-content-library. The Gallup World Poll data were accessed through an institutional subscription. Researchers seeking to use these data must obtain access directly from Gallup through their licensing process. Source data are provided with this paper.

## Code availability

The code and derived data products necessary to reproduce the analyses reported in this Article (excluding raw platform data restricted by third-party terms) are publicly available via the Open Science Framework at https://osf.io/3h478/overview.
