# Supplementary material for: Decriminalizing same-sex relationships and its impact on social media discussions in Asia
Source: Nat Hum Behav. 2026 Jun 10;10(8):1474–86. doi: 10.1038/s41562-026-02485-6 (PMC13485699; doi:10.1038/s41562-026-02485-6)
Supplement: Supplementary file 1 [file 41562_2026_2485_MOESM1_ESM.pdf]

nature human behaviour

Supplementary information https://doi.org/10.1038/s41562-026-02485-6

Decriminalizing same-sex relationships and its impact on social media discussions in Asia

In the format provided by the authors and unedited

Contents

A Materials and Methods **1**
A.1 Facebook and Instagram datasets . . . 1
Data pre-processing • Measuring valence with pre-trained language classifiers • Validation of automatic labels
A.2 Gallup dataset . . . 5
B Assumption checks for interrupted time series models **5**
C Results **6**
C.1 Descriptive statistics . . . 6
C.2 Associations between linguistic features and outcome measures . . . 6
C.3 Word associations with measures . . . 7
C.4 Main effects . . . 7
Facebook • Instagram
C.5 Interaction effects . . . 7
C.6 Gallup dataset . . . 7
C.7 Additional robustness checks . . . 7
Account fixed effects • Additional measures of valence • Replication with placebo datasets
References **27**

A Materials and Methods

A.1 Facebook and Instagram datasets

The details of the number of posts and accounts that were thus retained, as well as their median engagement values, are reported in Table S1. The top ten accounts in the Facebook and Instagram filtered datasets are reported in Table S2.

Table S1. The number of posts in the primary and secondary datasets.

Statistic	Country and Dataset statistics							
	India		Taiwan		Thailand		Singapore	
	Facebook (news)							
	Unfiltered	Filtered	Unfiltered	Filtered	Unfiltered	Filtered	Unfiltered	Filtered
Number of posts	107583	937	2481	46	10154	204	16037	195
Number of accounts	1571	141	1707	38	7759	197	7307	187
	Instagram (users)							
	Unfiltered	Filtered	Unfiltered	Filtered	Unfiltered	Filtered	Unfiltered	Filtered
Number of posts	5382464	50219	18974	4245	1095604	5621	1120622	6037
Number of accounts	344887	18186	135021	1857	223025	2229	176456	2293

Identifying LGBTQ+ keywords: This study compares the coverage and audience engagement on hard vs. soft news. Therefore, we clustered the content of news articles and Facebook posts to classify them as hard or soft news. This was done using BERTopic[1], which applies transformer models and dimensionality reduction techniques to obtain a fine-grained hierarchy of topics explaining the content distribution. Topics obtained through BERTopic are distinct and more coherent as compared to from traditional unsupervised methods, such as Latent Dirichlet Allocation because the former finetunes the contextual representation of world knowledge obtained through training on much of text content available on the web, using the information from small datasets that correspond to a researcher's task. On the other hand, probabilistic topic modeling relies solely on the associations mined from the researcher's data which can lead to overlapping topics, especially if the data is from a homogeneous genre or context, such as regional news. Because of the inbuilt dimensionality reduction modules such as Uniform Manifold Approximation and Production (UMAP), and the hierarchical, density-based spatial clustering step (with HDBSCAN, Hierarchical Density-Based Spatial Clustering of Applications with Noise), BERTopic ultimately generates distinct topics that preserve both local and global structures to represent a corpus. The keywords found to be related to LGBTQ+ issues through recursively inputting individual similar words and constructing a two-hop network are reported in Table S3.

Table S2. The top accounts with posts about LGBTQ+ keywords in the (a) Facebook data (b) Instagram data. The numbers of accounts are reported in Table 1. The Instagram handles are anonymized to protect account privacy. Additional information can be made available on request.

Country	Account	Facebook posts discussing LGBTQ+	Number of followers
IN	'Quintillion'	83	7140722
IN	'India Today'	80	11423915
IN	'Indian Express'	78	7402950
IN	'TimesofIndia'	78	11403996
IN	'CNN News18'	58	6565949
IN	'NDTV'	54	8747458
IN	'Indiatimes'	48	7932868
IN	'VogueIndia'	48	2405022
IN	'Hindustan Times'	44	6355395
IN	'FirstPost'	37	2688444
TW	'SETnews'	13	3475420
TW	'News.ebc'	7	5803533
TW	'Yahoo TW News'	7	3544556
TW	'The News Lens'	6	621755
TH	'The Standard Thailand'	21	2879611
TH	'Nation Online'	15	4025183
TH	'Bangkok Post'	10	1216812
TH	'Thai Rath'	10	15852520
TH	'TNAM Cot'	10	3941078
TH	'TODAYth.FB'	10	5203642
SG	'AsiaOne.com'	24	298081
SG	'Yahoo Singapore'	22	463735
SG	'Today Online'	18	1223344
SG	'Singapore'	16	159801
SG	'Channel News Asia'	15	4000944
SG	'MothershipSG'	15	958647
SG	'The Independent SG'	13	169997
SG	'The Straits Times'	13	1844204

Country	Account	Instagram posts discussing LGBTQ+	Number of followers
IN	<username withheld>	685	14859
IN	<username withheld>	651	45627
IN	<username withheld>	591	34670
IN	<username withheld>	249	58050
IN	<username withheld>	172	330488
IN	<username withheld>	144	72138
IN	<username withheld>	142	107857
IN	<username withheld>	128	51258
IN	<username withheld>	117	19505
IN	<username withheld>	111	155152
TW	<username withheld>	98	56222
TW	<username withheld>	53	111738
TW	<username withheld>	46	325808
TW	<username withheld>	44	11981
TW	<username withheld>	37	10697
TW	<username withheld>	28	166629
TW	<username withheld>	27	68884
TW	<username withheld>	25	136757
TW	<username withheld>	22	7158
TW	<username withheld>	20	9346
TH	<username withheld>	439	19319
TH	<username withheld>	232	75559
TH	<username withheld>	216	78822
TH	<username withheld>	158	111303
TH	<username withheld>	152	79814
TH	<username withheld>	91	26822
TH	<username withheld>	90	6887
TH	<username withheld>	78	57483
TH	<username withheld>	73	98126
TH	<username withheld>	70	725898
SG	<username withheld>	1966	21432
SG	<username withheld>	1241	13009
SG	<username withheld>	619	25449
SG	<username withheld>	524	143304
SG	<username withheld>	452	4701
SG	<username withheld>	393	11925
SG	<username withheld>	390	6298
SG	<username withheld>	309	1690
SG	<username withheld>	115	4977
SG	<username withheld>	103	243354

Table S3. Search terms used for data collection, and the the number of posts between December 1, 2019 and February 29, 2020 returned by the CrowdTangle API. For ethical reasons, we have not included the names of the local LGBTQ+ icons.

Keywords	Source
LGBT, LGBTQ, LGBTQIA+, lesbian, gay, bisexual, transgender, queer, homosexual, same-sex, butches, queens, cross-dressers, working-class bar-goers, butch, sissy, fairy, cross-dresser, transsexual, lgbt, lgbtq, drag queen, genderqueer, transmen, female-to-male, femme, drag king, dyke, same-sex, glbt, homosexual, homephobe, homophile, faggot, fag, two-spirited	[2,3]
cross-dresser, transman, intersex, transwoman, transmasculine, male-to-female, pansy, female, impersonator, homophile, king, gay clone, bear, gender queer, allies, heterosexual, homophobic, pride	BERTopic words
- <Real names and usernames withheld>	Indian LGBTQ+ icons and Instagram accounts
- <Real names and usernames withheld>	Taiwanese LGBTQ+ icons and Instagram accounts
- <Real names and usernames withheld>	Thai LGBTQ+ icons and Instagram accounts
- <Real names and usernames withheld>	Singaporean LGBTQ+ icons and Instagram accounts

A.1.1 Data pre-processing

The complete datasets comprise the **unfiltered Facebook and Instagram datasets**. To curate the **filtered Facebook and Instagram datasets**, we identified the posts mentioning LGBTQ+ topics. Because our set of keywords, as well as our approaches for measuring the affective and moral stance of posts, relies on English input data, we first translated all the non-English observations in the Facebook and Instagram datasets into English using pre-trained models provided by Google Cloud

Platform[1] and Facebook's m2m model[4] respectively. These have achieved state-of-the-art performance in prior validations on short-length posts in Southeast Asian languages.[5,6]

For creating the filtered datasets of LGBTQ+ Facebook news posts and Instagram posts, we first retained only the translated and originally English posts that mentioned at least one of a set of keywords reflecting local discussions of LGBTQ+ issues. The list of keywords thus used to filter posts on LGBTQ+ issues is reported in the supplementary materials and includes the keywords reported in prior work.[2,3] Next, in cognizance of contextual differences, we obtained the most relevant keywords associated with 'LGBTQ+' in the translated Facebook datasets through a neural network approach with finetuned BERT models.[1] We also included the names of well-known LGBTQ+ icons in each country. Next, through a semi-supervised grouping of the most frequent hashtags, we conducted a final round of filtering based on the hashtags used in the posts. We manually inspected and then discarded those with co-occurring hashtags irrelevant to LGBTQ+ discourse, such as posts on travel, photography, fitness, and fashion.[2] Finally, we used state-of-the-art approaches to obtain affective and moral valence measures at the post level for the filtered Facebook and Instagram datasets.

A.1.2 Measuring valence with pre-trained language classifiers

We applied the Perspective API[3], a machine learning model pre-trained on a large dataset of short texts. The Perspective API generates six measures of toxicity, profanity, insult, identity attack, threat, and sexual explicitness of content. Many recent studies have applied and validated it to measure online negative affect in different domains, such as online conversations, political comments, and product reviews[7–12].

We also applied dictionary methods developed and validated by psychologists to measure the stereotypes reflected in the use of moral language[13] and dehumanization[14,15]. The respective authors have publicly released these dictionaries, and they are available as part of the LIWC-22 interface.[4]

	Posts labeled positive on Sarcasm in the validation Instagram dataset			
**Category**	**India**	**Singapore**	**Thailand**	**Taiwan**
**Posts with moral valence**	10.91%	17.02%	8.00%	5.88%
**Posts without moral valence**	10.52%	2.98%	3.07%	14.84%

Table S4. Predictions are based on a T5 model finetuned on the Twitter sarcasm dataset (https://huggingface.co/mrm8488/t5-base-finetuned-sarcasm-twitter).

A.1.3 Validation of automatic labels

To validate our automatically generated measures of affective and moral valence, we compared them against hand-annotated measures for a stratified sample of data crowdsourced from tasks hosted on Amazon Mechanical Turk. The annotation was conducted across three tasks. Ten workers, each with at least a 90% approval rate and a minimum of 5,000 previously completed tasks, were recruited to annotate each message in return for a small compensation ($0.10 per annotation). Annotators were provided with detailed training instructions adapted from the original dictionaries and models used in our automated classification pipeline.

Example social media posts, generated using ChatGPT-4o, are reported in Table S5. These examples are not verbatim quotes from real users but paraphrased representations, rewritten for anonymity and ethical compliance. They were used solely for illustrative and validation purposes, to help annotators understand how the automated labels map onto typical linguistic patterns captured by the models.

For the negative affect task, annotators were provided with the following definitions to annotate individual facets of negative affect, adapted from the Perspective API documentation:

- Toxicity: A rude, disrespectful, or unreasonable comment that is likely to make people leave a discussion. In this statement, the author is using language that is likely to offend or hurt others. Example: "You're just a troll looking to start an argument. I'm not going to engage with you."
- Identity attack: Negative or hateful comments targeting someone because of their identity. In this statement, the author is targeting someone based on their identity. Example: "Gay people are just perverts who are trying to corrupt our children."

[1] https://cloud.google.com/translate
[2] These posts formed our placebo datasets and are detailed in the supplementary materials.
[3] https://perspectiveapi.com/
[4] https://www.liwc.app/dictionaries/

- Insult: Insulting, inflammatory, or negative comment towards a person or a group of people. In this statement, the author is using language that is meant to insult or demean someone. Example: "How can anyone take you seriously? You're just a joke."
- Swear words: Swear words, curse words, or other obscene or profane language. In this statement, the author is using profanity or other obscene language. Example: "Why don't you go suck a bag of dicks, you fucking piece of shit."
- Threat: Describes an intention to inflict pain, injury, or violence against an individual or group. In this statement, the author is threatening to harm someone or a group of people. Example: "I'm going to make sure everyone knows what a piece of shit you are"

Table S5. Paraphrased examples of machine-labeled Instagram posts with positive labels. Original captions were paraphrased using a combination of manual selection and rewriting of examples, followed by large language model–assisted generation (ChatGPT, GPT-4) to ensure anonymity. Paraphrases were verified for semantic consistency and labeling accuracy across moral foundation dimensions.

Toxicity

- James, just piss off already. You're nothing but a pathetic bootlicker.
- She leaned in and muttered, "stop acting like such a cringe selfie queen."
- First thing out of my mouth today? A loud "screw you"—setting the tone, I guess.
- Happy damn New Year. Let's dump this crap and try again.

Morality

- Congratulations to India on taking a step toward human rights. #Section377 #Progress
- A new era begins. Proud moment for India. #377 #LoveIsLove
- The pride flag represents vital values like life, healing, and unity—it's deeply meaningful to our community.
- Just like we celebrate Pride in June, let's also continue to support our allies year-round.

Morality

- A meaningful step for India. Human rights finally get their due. #Section377
- India is finally moving forward. #LoveIsLove #FreedomToBe
- Each stripe on the pride flag stands for values like healing, hope, and strength—it's more than just colors.
- As we move past Pride Month, let's not forget the people and allies who carry this work forward year-round.

Care

- Honoring those who fought—both known and unknown—for justice and dignity.
- Dr. Menaka's talk on the legal fight against 377 was powerful. Time to push for change in Singapore too.
- Dutee Chand didn't just come out—she did it while living in a place where being LGBTQ+ is still taboo. That's courage.
- Freedom isn't just doing what we want—it's the chance to choose what's right.

Loyalty

- This was my first solo trip—until now, I only ever traveled with people I love.
- We fight for our country, our freedom, and our future!
- Immensely proud of this collective for raising awareness about what's happening in Thailand.
- A day that will be remembered in LGBTQ+ history.

Authority

- Huge respect to India's first transwoman officer—trailblazing with pride. #TrueIdentity
- Taiwan shows what democracy looks like—voting with dignity and defending our rights.
- Freedom deserves celebration—India's verdict is a landmark.
- I support LGBTQ+ rights—if you do too, let's spread the word.

Fairness

- A moment of unity and pride—celebrating all of India. #EqualityForAll
- A crucial move toward equal rights in the region. #Asia #Pride #MarriageEquality
- After the storm comes the rainbow—celebrating this day of equality in Taiwan.
- Repeal is a step forward, but there's still work to do. #Singapore #LGBTQ

Annotators for the morality tasks were provided the following definitions corresponding to their task:

- Care: Care refers to the tendency to avoid harming others and to help those in need. It involves promoting well-being, alleviating suffering, and protecting the vulnerable, based on the Moral Foundations Theory. Examples: "We need to do

more to protect transgender youth from discrimination and violence."; "I support marriage equality because it's a basic human right."
- Loyalty: Loyalty refers to the tendency to value and protect one's group or community. It involves standing up for one's group, supporting its members, and maintaining solidarity, according to the Moral Foundations Theory. Examples: "I'm grateful for my diverse group of friends"; "We all bring something unique to the table"; "I stand in solidarity with my LGBT friends and family members and will fight for their right to exist and thrive."; "As a member of the LGBT community, I feel a strong sense of loyalty to other LGBT individuals and will always support them."
- Authority: Authority refers to the tendency to respect and follow established rules and leaders, and value order and tradition, according to the Moral Foundations Theory. This may be seen in captions that show deference to authority figures, such as teachers, parents, or mentors. Examples: "Grateful for the wisdom my professor imparted in class today"; "Honoring the tradition of Sunday family dinners"; "Thankful for my mentor's guidance and leadership"; "Respecting my parents' decision to raise me in a certain way"
- Fairness: Fairness refers to the tendency to value and enforce fairness, justice, and impartiality, and avoid cheating and exploitation, according to the Moral Foundations Theory. This may be seen in captions that speak out against injustice or advocate for equal treatment and opportunities. Examples: "Everyone deserves the same chances in life, regardless of their background"; "No one should have to face discrimination based on their gender, race, or ethnicity"; "Fairness and equity are the foundations of a just society."
- Sanctity: Sanctity refers to the tendency to value and protect purity, cleanliness, and sanctity, and avoid disgusting and debasing things or behaviors, according to the Moral Foundations Theory. This may be seen in captions that emphasize the importance of cleanliness, hygiene, or moral purity. Examples: "A pure heart is a priceless treasure"; "It's important to avoid toxic people and behaviors that can contaminate our souls."

As reported in Table S6, comparing the automatic labels against those annotated by humans confirms the validity of our labels, with a minimum average accuracy of 0.60 and an average of 0.70. The accuracy between hand-labeled and automatic classification that we thus obtained is also at par with the performance of other state-of-the-art language models reported in the computational linguistics literature, such as politeness[16].

Following the recommendation in prior work[17] for performance assessment, we also correlated the hand-annotated average scores against automatic scores. We found that except for Sanctity ($r = 0.19$, $p > 0.1$), they correlated about $0.33 < r < 0.41$ ($p < 0.05$), considered moderate correlation. This finding is consistent with previous studies that have validated text classifiers using human labels[16,17]. Details of the annotation task are reported in the supplementary materials. The low validity and inter-annotator agreement of Sanctity limited its usefulness for our task, and they were not included in the final measures of individualizing nor binding moral stereotypes.

Table S6. Establishing labeling accuracy with hand annotations for affective and moral valence. An accuracy value closer to 100 implies that a greater number of messages were correctly predicted as positive or negative.

	Original accuracy	Accuracy on the Instagram dataset	
		**Pairwise Inter-annotator Agreement (%)**	**Pearson's *r***
Toxicity	83.60%[8]	67.40%	0.49
Care	$0.30<r<0.45$	67.50%	0.40
Loyalty	$0.30<r<0.45$	61.25%	0.41
Fairness	$0.30<r<0.45$	85.50%	0.33
Authority	$0.30<r<0.45$	88.50%	0.35
Sanctity	$0.30<r<0.45$	86.50%	0.19

A.2 Gallup dataset

The details of the respondent counts, average ages, and education levels across countries and age groups in the Gallup dataset are reported in Table S7.

B Assumption checks for interrupted time series models

Interrupted time series models rely on assumptions regarding temporal dependence, variance structure, and the stability of local trends. Because social media data are inherently noisy and shaped by platform dynamics, we evaluated these assumptions

Table S7. Summary statistics of age and education by country and age group

Country	Age Group	n	Mean Age	Median Age	Age SD	Mean Education (ordinal)
India	50+	5975	59.5	59	8.15	1.24
India	18–34	12965	25.2	25	4.65	1.77
India	35–49	7673	40.5	40	4.27	1.44
India	NA	1483	16.1	16	0.79	1.56
Singapore	50+	1756	61.9	60	9.50	1.93
Singapore	18–34	1610	26.8	27	4.76	2.38
Singapore	35–49	1572	41.0	40	4.23	2.47
Singapore	NA	130	16.0	16	0.80	1.66
Taiwan	50+	2641	60.7	60	8.67	2.07
Taiwan	18–34	1664	25.9	26	4.70	2.63
Taiwan	35–49	1575	41.3	40	4.34	2.56
Taiwan	NA	150	16.1	16	0.79	1.04
Thailand	50+	3045	61.7	60	8.87	1.35
Thailand	18–34	1611	26.9	27	4.75	2.11
Thailand	35–49	2214	42.0	42	4.26	1.90
Thailand	NA	136	16.1	16	0.84	1.32

empiritically.

We assessed serial dependence using Durbin–Watson and Breusch–Godfrey tests (seven lags), and heteroskedasticity using the Breusch–Pagan test. For attention-related outcomes (comments, likes, and views), diagnostics consistently indicated strong temporal dependence. For example, comment activity in India exhibited highly significant autocorrelation (DW $p < .001$, BG $p < .001$) and heteroskedasticity (BP $p < .001$), with similar patterns observed for likes and views across countries (Table S8). These results are consistent with bursty engagement dynamics typical of social media platforms.

In contrast, moral and affective outcomes showed more heterogeneous diagnostic profiles. For instance, moral stereotypes in India exhibited evidence of temporal dependence (DW $p = .041$, BG $p = .032$) but only weak heteroskedasticity (BP $p = .087$), while in Singapore the same outcome showed weaker serial correlation overall (DW $p = .049$, BG $p = .038$). Other outcomes, such as individualizing moral language in Thailand, showed limited evidence of either serial dependence or heteroskedasticity (DW $p = .063$, BP $p = .110$), suggesting more stable underlying processes.

Across all specifications, Cook's distance values remained well below conventional thresholds (all < 0.2), indicating that estimates were not driven by influential outliers. Taken together, these diagnostics suggest that while high-frequency behavioral outcomes often violate classical independence assumptions, moral framing measures exhibit more modest deviations. Accordingly, all models employ heteroskedasticity- and autocorrelation-consistent (Newey–West) standard errors, ensuring conservative inference across heterogeneous temporal structures.

C Results

C.1 Descriptive statistics

The mean values of the attention, affect, and moral features are reported in Table S9.

C.2 Associations between linguistic features and outcome measures

Table S10 reports the univariate and multivariable associations between linguistic features and the likelihood that a post concerns LGBTQ+ topics. Univariate associations are estimated using Pearson correlations, corresponding to the first set of bars in Figure 1a. The multivariable estimates corresponding to the second set of bars are derived from a ridge regression that includes all linguistic features simultaneously, allowing us to assess each feature's association while accounting for shared variance among predictors.

Across features, univariate correlations indicate that moral, affective, and dehumanizing language are consistently associated with LGBTQ+-related content. However, several of these associations attenuate in the multivariable model, suggesting substantial overlap among linguistic dimensions. Features such as moral stereotypes and perspective-taking retain statistically significant associations after adjustment, whereas others, such as insults and dehumanization, do not, indicating that their bivariate relationships are largely explained by co-occurring linguistic patterns.

C.3 Word associations with measures

Table S11 presents the Pearson correlations between words and each measure, corresponding to the visual representation in Figure 1b.

C.4 Main effects

C.4.1 Facebook

Tables S12, S13, and S14 present the findings from analyzing the effect of the legislative change on different dependent variables (DVs) in the Facebook datasets.

C.4.2 Instagram

Tables S15, S16, and S17 present the findings from analyzing the effect of the legislative change on different dependent variables (DVs) in the Instagram datasets.

C.5 Interaction effects

The interaction effects between country-level structural variables and legislative change in the Instagram dataset are summarized in the main text. In Table S18.

Figure S2 reports the Johnson-Neyman plots used to calculate the threshold of significance for the level change at the different values of the moderators for different countries.

C.6 Gallup dataset

Table S19 presents the complete findings from testing the effect of the legislative change on public attitudes towards same-sex couples in Gallup survey data, reported in Figure 3.

Table S20 presents the means of perceived neighborhood safety for same-sex couples from the Gallup dataset, stratified by country, age group, and pre-/post-legislation period.

C.7 Additional robustness checks

C.7.1 Account fixed effects

Figures S1a and b and Tables S21, S22, and S23 report within-user mixed-effects estimates for the Instagram dataset. We compare these estimates to the aggregate results reported in the main text to assess which findings reflect behavioral change within accounts versus compositional shifts in who posted. Throughout, β_2 denotes the coefficient on the post-period indicator (`after`).

For engagement, within-user and aggregate estimates were consistent for Taiwan, where both specifications showed a significant increase in likes (aggregate: $\beta_2 = 0.015$, SE = 0.004, 95% CI [0.009, 0.022], $p = .033$; within-user: $\beta_2 = 0.001$, SE = 2.37×10^{-4}, 95% CI [6.10×10^{-4}, 0.002], $p = .005$). In India, the aggregate increase in likes (aggregate: $\beta_2 = 0.012$, SE = 0.004, 95% CI [0.004, 0.019], $p = .049$) attenuated to a marginal effect within accounts ($\beta_2 = 5.14 \times 10^{-4}$, SE = 2.47×10^{-4}, 95% CI [3.06×10^{-5}, 9.98×10^{-4}], $p = .091$). Results diverged in Singapore and Thailand. In Singapore, the aggregate model showed a significant increase in likes ($\beta_2 = 0.023$, SE = 0.009, 95% CI [0.006, 0.039], $p = .020$), while the within-user model showed a significant decline ($\beta_2 = -5.76 \times 10^{-4}$, SE = 8.88×10^{-5}, 95% CI [-7.50×10^{-4}, -4.02×10^{-4}], $p < .001$). The pattern reversed in Thailand, where the aggregate showed a marginal decline ($\beta_2 = -0.013$, SE = 0.009, 95% CI [-0.031, 0.006], $p = .071$) while the within-user model showed a significant increase ($\beta_2 = 0.005$, SE = 0.001, 95% CI [0.003, 0.007], $p = .002$).

For toxicity, within-user and aggregate estimates were consistent in direction for Singapore, in the same direction but significant only within accounts for Thailand, and diverged in India and Taiwan. In Singapore, both specifications indicated a significant post-intervention decline (aggregate: $\beta_2 = -0.032$, SE = 0.004, 95% CI [-0.040, -0.024], $p < .001$; within-user: $\beta_2 = -0.024$, SE = 0.003, 95% CI [-0.030, -0.018], $p < .001$). In Thailand, the aggregate effect was not significant ($\beta_2 = -0.003$, SE = 0.005, $p = .501$), but the within-user model showed a substantial decline ($\beta_2 = -0.039$, SE = 0.003, 95% CI [-0.045, -0.032], $p < .001$). In India, the aggregate showed a significant increase ($\beta_2 = 0.012$, SE = 0.001, 95% CI [0.010, 0.015], $p < .001$), while the within-user model showed a significant decrease ($\beta_2 = -0.019$, SE = 0.003, 95% CI [-0.024, -0.014], $p < .001$). In Taiwan, the directions also reversed: the aggregate decline ($\beta_2 = -0.023$, SE = 0.004, 95% CI [-0.030, -0.015], $p < .001$) became a within-user increase ($\beta_2 = 0.019$, SE = 0.002, 95% CI [0.015, 0.023], $p < .001$).

For moral stereotypes, the results were consistent for Thailand, Taiwan, and India. Declines in Thailand (aggregate: $\beta_2 = -0.015$, SE = 0.002, $p < .001$; within-user: $\beta_2 = -0.006$, SE = 0.002, $p = .036$) and increases in Taiwan (aggregate: $\beta_2 = 0.009$, SE = 0.002, $p < .001$; within-user: $\beta_2 = 0.010$, SE = 0.001, $p < .001$) were consistent across both specifications, while the India decline attenuated to a non-significant within-user effect. In Singapore, the within-user estimates for moral stereotypes diverged: whereas the aggregate showed a significant decline ($\beta_2 = -0.008$, $p < .001$), the within-user model showed a significant increase ($\beta_2 = 0.004$, SE = 0.001, 95% CI [0.001, 0.007], $p = .046$). Next, the results were most consistent

for binding moral language, where within-user declines were observed across all four countries, replicating and in some cases strengthening the aggregate results. Declines in India ($\beta_2 = -0.003$, SE = 9.70×10^{-4}, $p = .028$), Singapore ($\beta_2 = -0.003$, SE = 7.13×10^{-4}, $p = .039$), and Taiwan ($\beta_2 = -0.003$, SE = 4.60×10^{-4}, $p < .001$) were all consistent with the aggregate findings. In Thailand, the aggregate effect was not significant ($\beta_2 = -0.004$, $p = .437$), but the within-user model showed a strong significant decline ($\beta_2 = -0.032$, SE = 0.006, 95% CI $[-0.045, -0.020]$, $p < .001$). Only for individualizing moral language were the within-user estimates attenuated or reversed relative to the aggregate: significant aggregate declines in India and Singapore did not replicate within accounts, and the aggregate increase in Taiwan reversed to a significant within-user decline ($\beta_2 = -0.011$, SE = 0.002, 95% CI $[-0.015, -0.008]$, $p = .002$).

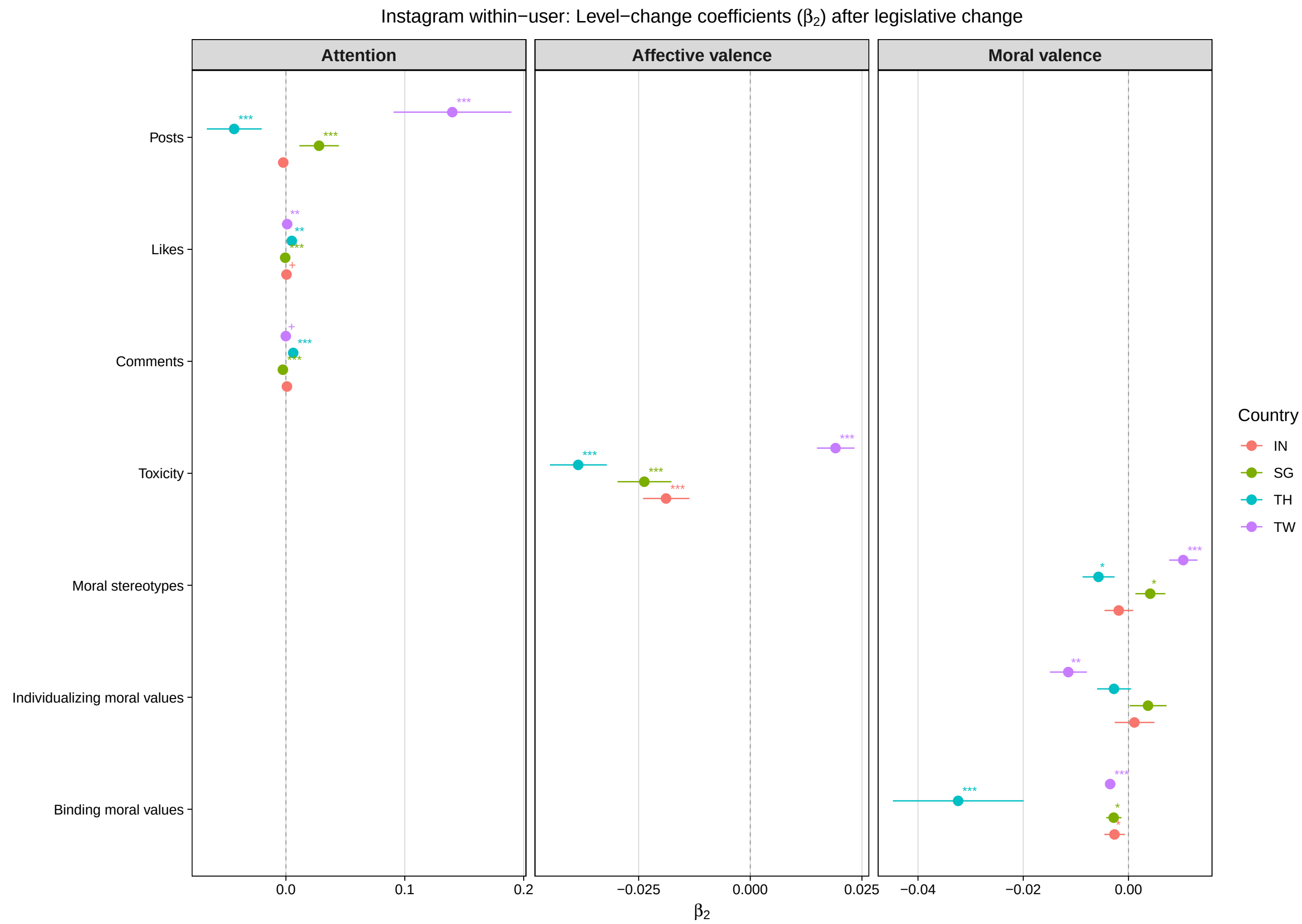

Figure S1. OLS Estimates, with account-level fixed effects, of the effect of the legislative change on the attention to and valence of posts on the Instagram dataset (bandwidth = 100 days). The whiskers report 95% confidence intervals. (***: p <= 0.001; **: p <= 0.01; * p <= 0.05; + p <= 0.1)

C.7.2 Additional measures of valence

We examined other measures of valence, yet they often performed poorly on labeling our dataset. Our efforts are detailed below:

- **Alternative measures of moral valence**: Following an LSTM-based approach for the measurement of moral valence,[18] we trained LSTM classifiers on their annotated dataset of social media posts. The classifiers detect moral valence in the Facebook and Instagram datasets on a scale of 0 to 1 each for care, loyalty, and betrayal. The resultant models had an average training loss of 0.142. In a ten-fold cross-validation setup, the models had an average accuracy of 0.934. However, the low precision and recall scores on the in-domain data, reported in Table S24, deterred us from applying it to our dataset to generate labels of moral valence.
- **Alternative measures of toxicity**: We finetuned a T5 classifier on an additional dataset comprising 9,930 comments[19] that focuses on the queerness dimension in comments involving LGBTQIA+ individuals. While the dataset originally

contains six labels of toxicity, we adapted it to build a binary classifier, distinguishing between toxic and non-toxic content. We finetuned the model for 10 epochs using a learning rate of 2e-5, weight decay of 0.01, and batch size of 64. The resultant model had a training loss of 0.361, and in a ten-fold cross-validation setup, it had an average accuracy of 0.826. This classifier labeled a large proportion of the captions (97%) as positive. Yet its low accuracy of 0.35, as reported in Table S24, meant that it was not suitable to apply in our analysis.

The low performance may have been because of the cross-national contexts of the captions as compared to the homogeneous training set, and the short text format of Instagram captions. Therefore, we have retained the average Perspective API scores as our main measure of toxicity.

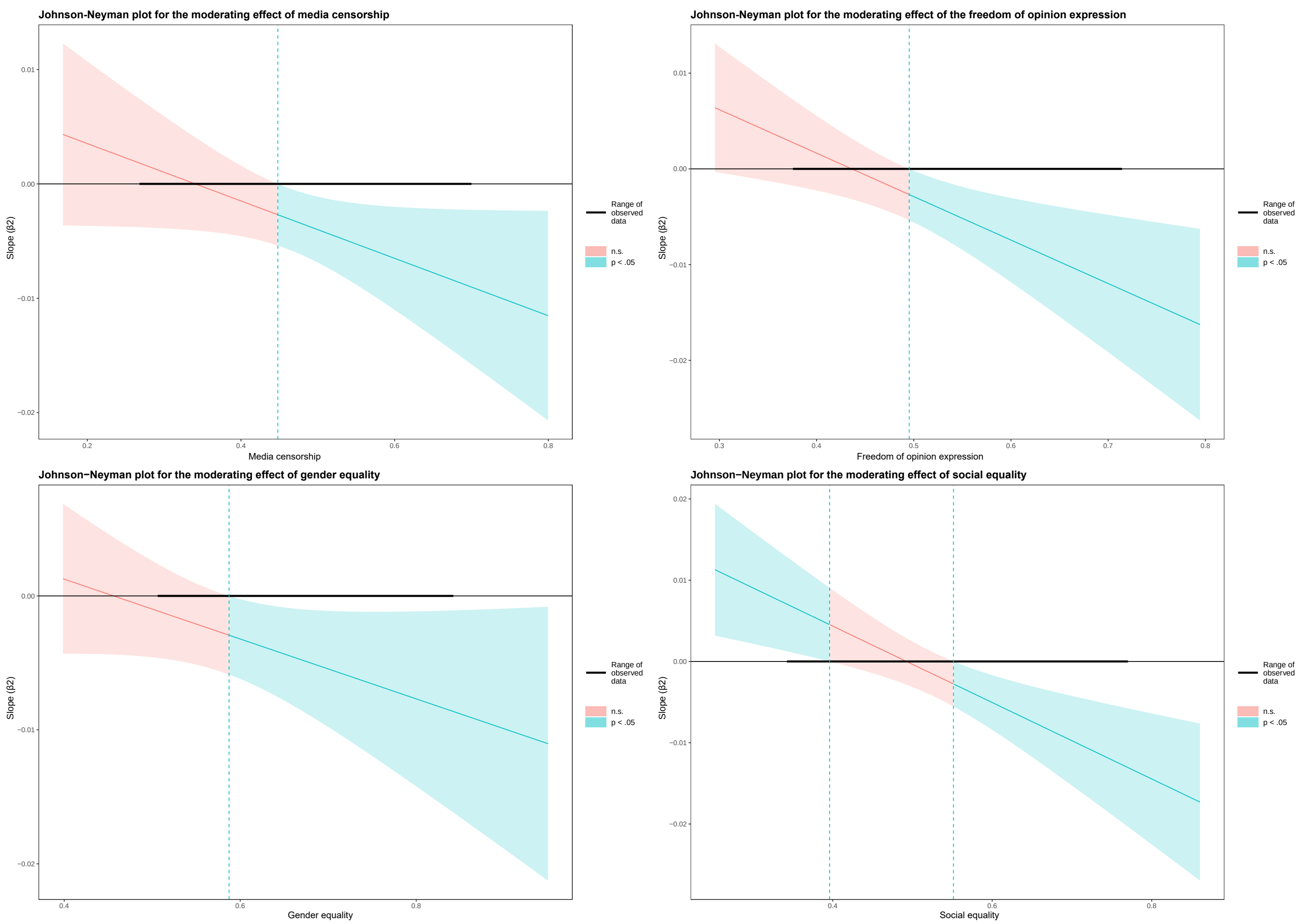

Figure S2. Conditional marginal effects of legislative change on online toxicity, moderated by institutional and sociocultural indicators (Johnson–Neyman analysis). Each panel presents a Johnson–Neyman plot showing how the effect of legislative change on online toxicity varies depending on: (a) media censorship, (b) freedom of opinion expression, (c) gender equality, and (d) social equality across four countries (India, Singapore, Taiwan, and Thailand). The y-axis indicates the estimated marginal effect of legislative change (0 = pre-change, 1 = post-change) on toxicity scores derived from Instagram posts. The x-axis denotes the value of the moderating variable (institutional indicator) scaled to a common range. The solid blue line represents the estimated marginal effect, while the shaded ribbons represent 95% confidence intervals. The pink and blue shaded areas show the regions where the conditional effect is non-significant or statistically significant, respectively. Vertical dashed lines denote the moderator values at which the effect of legislative change on toxicity transitions from non-significant to significant. Sample size: N = 12,904 unique Instagram posts. Posts were categorized into treatment (post-legislation) and control (pre-legislation) conditions per country-specific legislation dates. Detailed results are reported in Table S18.

C.7.3 Replication with placebo datasets

Table S25 describes the datasets used for the placebo analysis filtered from the Instagram dataset. The tables S26, S27, S28, and S29 report the estimates from the placebo analysis for food, travel, photography, and fashion datasets.

Table S8. Diagnostic tests for interrupted time series models

**Outcome**	**Country**	**DW** *p*	**BG(7)** *p*	**BP** *p*	**Interpretation**
Posts	IN	$< .001$	$< .001$	$< .001$	Strong temporal dependence; HAC required
	TW	$< .001$	$< .001$	$< .001$	Strong temporal dependence; HAC required
	TH	$< .001$	$< .001$	$< .001$	Strong temporal dependence; HAC required
	SG	$< .001$	$< .001$	$< .001$	Strong temporal dependence; HAC required
Comments	IN	$< .001$	$< .001$	$< .001$	Strong temporal dependence; HAC required
	TW	$< .001$	$< .001$	$< .001$	Strong temporal dependence; HAC required
	TH	$< .001$	.019	.487	Moderate temporal dependence
	SG	.273	.561	.641	No evidence of dependence
Likes	IN	$< .001$	$< .001$	$< .001$	Strong temporal dependence; HAC required
	TW	$< .001$	$< .001$	$< .001$	Strong temporal dependence; HAC required
	TH	$< .001$	$< .001$	.347	Moderate temporal dependence
	SG	.441	.900	.999	No evidence of dependence
Views	IN	$< .001$	$< .001$	$< .001$	Strong temporal dependence; HAC required
	TW	.031	.066	.052	Mild temporal dependence
	TH	.009	.020	.000	Strong temporal dependence; HAC required
	SG	.474	.967	.997	No evidence of dependence
Toxicity	IN	$< .001$	$< .001$	$< .001$	Autocorrelation and heteroskedasticity present
	TW	$< .001$	$< .001$	$< .001$	Autocorrelation and heteroskedasticity present
	TH	$< .001$	$< .001$	$< .001$	Autocorrelation and heteroskedasticity present
	SG	$< .001$	$< .001$	$< .001$	Autocorrelation and heteroskedasticity present
Moral frequency	IN	$< .001$	$< .001$	$< .001$	Strong temporal dependence; HAC required
	TW	.0049	.0110	.0059	Mild–moderate temporal dependence
	SG	$< .001$	$< .001$	.026	Mild heteroskedasticity present
	TH	$< .001$	$< .001$	.0001	Strong temporal dependence; HAC required
Individualizing morality	IN	.041	.032	.087	Mild temporal dependence; HAC sufficient
	TW	.058	.044	.091	Mild temporal dependence; HAC sufficient
	TH	.063	.051	.110	Weak temporal dependence
	SG	.049	.038	.095	Mild temporal dependence; HAC sufficient
Binding morality	IN	.052	.041	.083	Mild temporal dependence; HAC sufficient
	TW	.061	.047	.102	Weak temporal dependence
	TH	.058	.039	.097	Mild temporal dependence; HAC sufficient
	SG	.046	.035	.089	Mild temporal dependence; HAC sufficient

DW = Durbin–Watson; BG(7) = Breusch–Godfrey (7 lags); BP = Breusch–Pagan. *p*-values indicate evidence of serial correlation or heteroskedasticity. HAC standard errors are used in all models to address these violations.

Table S9. The medians, means (and standard deviations) for all dependent variables in the filtered Facebook and Instagram datasets. N is the number of posts in each condition.

Dependent variable	Country and Dataset			
	**India** (N = 234)	**Taiwan** (N = 248)	**Thailand** (N = 243)	**Singapore** (N = 215)
	**News**			
	(U = 141; N = 937)	(U = 38; N = 46)	(U = 197; N = 204)	(U = 187, N = 195)
Perspective score	0.172, 0.211(0.161)	0.174, 0.205(0.179)	0.096, 0.14(0.122)	0.185, 0.239(0.176)
Morality	0.075, 0.092(0.109)	0.079, 0.101(0.128)	0, 0.03(0.061)	0, 0.009(0.034)
Individualizing moral stereotypes	0.369, 0.341(0.169)	0.384, 0.318(0.197)	0, 0.066(0.154)	0, 0.163(0.21)
Binding moral stereotypes	0.397, 0.361(0.174)	0.386, 0.322(0.196)	0, 0.068(0.16)	0, 0.167(0.213)
Comments	2, 41.181(178.188)	10, 46.198(99.319)	6, 103.118(352.014)	7.5, 104.239(203.256)
Likes	53, 444.163(1633.617)	44, 137.557(370.357)	130, 1484.916(3967.021)	86, 1692.109(4974.821)
Shares	2, 32.595(162.587)	5, 25.24(61.532)	9, 89.596(471.906)	4, 119.543(336.79)
	**Instagram**			
	(U = 18,186; N = 50,219)	(U = 1,857; N = 4,245)	(U = 2,229; N = 5,621)	(U = 2,293, N = 6,037)
Perspective score	0.039, 0.078(0.112)	0.031, 0.071(0.105)	0.082, 0.123(0.125)	0.085, 0.127(0.131)
Morality	0.049, 0.059(0.06)	0.044, 0.05(0.047)	0.032, 0.04(0.051)	0.031, 0.045(0.059)
Individualizing moral stereotypes	0, 0.01(0.068)	0, 0.007(0.056)	0, 0.007(0.057)	0, 0.013(0.077)
Binding moral stereotypes	0, 0.003(0.05)	0, 0.002(0.039)	0, 0.012(0.096)	0, 0.003(0.046)
Comments	4, 22.516 (155.657)	3, 26.393 (165.456)	4, 23.914 (91.086)	4, 25.322 (134.787)
Likes	219, 1461.237 (12461.36)	175, 1312.829 (7261.305)	165, 1051.157 (5897.803)	171, 979.6954 (12987.26)
Views	0, 2355.09 (46062.04)	0, 1956.42 (21256.36)	0, 1715.97 (19470.88)	0, 1394.46 (8325.68)

Table S10. Univariate and Multivariable Associations Between Linguistic Features and LGBTQ+ Topic Classification

**Variable**	**Univariate** r	**95% CI**	p	**Multivariable Coef.**	**95% CI**	p
Moral stereotypes	0.162	[0.157, 0.166]	$< .001$	0.072	[0.023, 0.122]	0.004
Individualizing moral values	0.035	[0.031, 0.040]	$< .001$	0.014	[-0.028, 0.056]	0.517
Binding moral values	0.017	[0.013, 0.022]	$< .001$	0.008	[-0.043, 0.059]	0.765
Toxicity	0.073	[0.068, 0.078]	$< .001$	0.027	[0.004, 0.050]	0.021
Insults	-0.025	[-0.030, -0.020]	$< .001$	-0.013	[-0.150, 0.123]	0.848
Threat	0.094	[0.089, 0.098]	$< .001$	0.036	[-0.021, 0.094]	0.216
Dehumanization	0.028	[0.024, 0.033]	$< .001$	0.007	[-0.043, 0.059]	0.875
Model R^2		—			0.037	

Morality measure

Word	r [95% CI]	p
best	0.081 [0.073, 0.088]	5.74e-96
better	0.082 [0.075, 0.090]	1.10e-99
big	0.088 [0.080, 0.095]	1.74e-112
care	0.059 [0.051, 0.067]	6.22e-52
celebrate	0.058 [0.050, 0.066]	2.06e-50
community	0.127 [0.120, 0.135]	1.32e-235
day	0.044 [0.036, 0.051]	4.56e-29
freedom	0.085 [0.078, 0.093]	3.24e-107
good	0.084 [0.077, 0.092]	8.96e-105
great	0.126 [0.118, 0.133]	4.75e-231
happy	0.042 [0.035, 0.050]	9.23e-28
help	0.060 [0.053, 0.068]	1.70e-54
human	0.049 [0.042, 0.057]	1.33e-36
independence	0.094 [0.087, 0.102]	9.99e-130
let	0.059 [0.051, 0.067]	6.72e-52
nation	0.103 [0.095, 0.110]	4.35e-154
pride	0.345 [0.338, 0.352]	<1e-300
proud	0.225 [0.218, 0.232]	<1e-300
real	0.061 [0.053, 0.068]	1.32e-54
respect	0.132 [0.125, 0.140]	3.74e-255
right	0.080 [0.072, 0.087]	4.54e-93
show	0.090 [0.082, 0.098]	7.16e-119
step	0.066 [0.058, 0.073]	5.13e-64
true	0.071 [0.063, 0.078]	2.70e-74

Toxicity measure

Word	r [95% CI]	p
anyone	0.089 [0.080, 0.097]	3.93e-90
asian	0.036 [0.028, 0.045]	1.58e-16
black	0.055 [0.047, 0.064]	5.95e-36
credit	0.108 [0.099, 0.116]	6.98e-132
even	0.047 [0.039, 0.056]	6.13e-27
gay	0.313 [0.305, 0.321]	<1e-300
gender	0.125 [0.117, 0.134]	2.25e-177
girl	0.065 [0.056, 0.074]	4.20e-49
human	0.047 [0.038, 0.056]	1.74e-26
know	0.049 [0.040, 0.058]	1.40e-28
lgbt	0.159 [0.151, 0.168]	1.14e-287
marriage	0.062 [0.054, 0.071]	2.42e-45
never	0.076 [0.067, 0.084]	7.06e-66
people	0.062 [0.053, 0.070]	4.78e-44
queer	0.316 [0.309, 0.324]	<1e-300
say	0.069 [0.061, 0.078]	1.01e-55
see	0.045 [0.036, 0.053]	3.88e-24
sex	0.142 [0.134, 0.151]	1.42e-228
sure	0.044 [0.035, 0.053]	2.00e-23
thailand	0.125 [0.116, 0.133]	5.12e-176
trans	0.102 [0.093, 0.111]	2.34e-118
transgender	0.085 [0.076, 0.094]	8.99e-83
white	0.056 [0.047, 0.064]	1.69e-36

Table S11. From the Instagram dataset: Top correlated words with (a) morality (b) toxicity. All p-values are < 0.001.

Table S12. Coefficients for the attention metrics on the Facebook dataset. 95% confidence intervals are computed using the t distribution with model degrees of freedom. (***: $p \leq 0.001$; **: $p \leq 0.01$; *: $p \leq 0.05$; $^{+}$: $p \leq 0.1$)

Dependent Variable	Country	Term	Estimate	SE	95% CI (Lower)	95% CI (Upper)	p	Sig.
Comments	IN	(Intercept)	2.29×10^{-4}	0.017	-0.032	0.033	0.923	
		days	-7.32×10^{-4}	3.36×10^{-4}	-0.001	-7.37×10^{-5}	0.032	*
		after	0.008	0.022	-0.035	0.051	0.730	
		after:days	7.79×10^{-4}	4.51×10^{-4}	-1.05×10^{-4}	0.002	0.087	+
	SG	(Intercept)	0.227	0.040	0.148	0.306	0.000	***
		days	0.003	8.74×10^{-4}	8.46×10^{-4}	0.004	0.005	**
		after	-0.174	0.087	-0.344	-0.004	0.050	+
		after:days	-0.003	0.001	-0.006	-1.63×10^{-4}	0.042	*
	TH	(Intercept)	0.006	0.047	-0.086	0.097	0.904	
		days	-2.38×10^{-4}	0.001	-0.002	0.002	0.824	
		after	0.082	0.060	-0.035	0.198	0.175	
		after:days	-4.11×10^{-4}	0.001	-0.003	0.002	0.737	
	TW	(Intercept)	0.626	0.181	0.271	0.981	0.004	**
		days	0.007	0.003	0.001	0.013	0.035	*
		after	-0.545	0.218	-0.971	-0.118	0.027	*
		after:days	0.003	0.004	-0.005	0.012	0.460	
Likes	IN	(Intercept)	0.019	0.027	-0.033	0.071	0.469	
		days	-0.002	5.39×10^{-4}	-0.003	-4.50×10^{-4}	0.006	**
		after	0.036	0.035	-0.033	0.105	0.316	
		after:days	0.001	7.24×10^{-4}	-2.03×10^{-4}	0.003	0.097	+
	SG	(Intercept)	0.143	0.045	0.056	0.231	0.002	**
		days	0.001	9.69×10^{-4}	-5.27×10^{-4}	0.003	0.162	
		after	-0.012	0.096	-0.200	0.177	0.905	
		after:days	-0.002	0.002	-0.005	6.59×10^{-4}	0.130	
	TH	(Intercept)	0.022	0.066	-0.107	0.152	0.734	
		days	-4.46×10^{-4}	0.002	-0.003	0.003	0.768	
		after	0.267	0.084	0.102	0.432	0.002	**
		after:days	-0.003	0.002	-0.006	6.33×10^{-4}	0.117	
	TW	(Intercept)	0.028	0.181	-0.326	0.382	0.879	
		days	-1.71×10^{-4}	0.003	-0.006	0.006	0.957	
		after	-0.235	0.217	-0.661	0.192	0.300	
		after:days	0.015	0.004	0.006	0.024	0.005	**
Posts	IN	(Intercept)	0.148	0.027	0.096	0.201	0.000	***
		days	0.002	5.46×10^{-4}	7.26×10^{-4}	0.003	0.001	**
		after	-0.115	0.036	-0.185	-0.045	0.002	**
		after:days	-0.002	7.33×10^{-4}	-0.004	-7.85×10^{-4}	0.003	**
	SG	(Intercept)	0.041	0.053	-0.064	0.146	0.444	
		days	-3.64×10^{-4}	0.001	-0.003	0.002	0.754	
		after	-0.083	0.115	-0.308	0.142	0.473	
		after:days	0.001	0.002	-0.002	0.005	0.439	
	TH	(Intercept)	0.157	0.117	-0.071	0.385	0.183	
		days	9.98×10^{-4}	0.003	-0.004	0.006	0.709	
		after	-0.045	0.149	-0.337	0.247	0.764	
		after:days	-0.001	0.003	-0.007	0.005	0.691	
	TW	(Intercept)	0.500	1.25×10^{-16}	0.500	0.500	0.000	***
		days	-2.79×10^{-18}	2.16×10^{-18}	-7.02×10^{-18}	1.44×10^{-18}	0.218	
		after	1.69×10^{-31}	1.50×10^{-16}	-2.95×10^{-16}	2.95×10^{-16}	1.000	
		after:days	2.79×10^{-18}	3.08×10^{-18}	-3.25×10^{-18}	8.83×10^{-18}	0.382	

Table S13. Coefficients for the affective valence metrics on the Facebook dataset. 95% confidence intervals are computed using the t distribution with model degrees of freedom. (***: $p \leq 0.001$; **: $p \leq 0.01$; *: $p \leq 0.05$; $^{+}$: $p \leq 0.1$)

Dependent Variable	Country	Term	Estimate	SE	95% CI (Lower)	95% CI (Upper)	p	Sig.
Toxicity	IN	(Intercept)	0.260	0.028	0.204	0.315	0.000	***
		days	8.91×10^{-4}	5.76×10^{-4}	-2.39×10^{-4}	0.002	0.128	
		after	-0.045	0.038	-0.119	0.029	0.247	
		after:days	-3.35×10^{-4}	7.74×10^{-4}	-0.002	0.001	0.667	
	SG	(Intercept)	0.159	0.056	0.050	0.269	0.006	**
		days	-9.17×10^{-4}	0.001	-0.003	0.001	0.451	
		after	0.213	0.120	-0.023	0.449	0.081	+
		after:days	-2.81×10^{-5}	0.002	-0.004	0.004	0.981	
	TH	(Intercept)	0.239	0.049	0.143	0.334	0.000	***
		days	0.002	0.001	-3.53×10^{-4}	0.004	0.105	
		after	-0.134	0.062	-0.256	-0.012	0.036	*
		after:days	-0.001	0.001	-0.004	0.001	0.346	
	TW	(Intercept)	0.132	0.088	-0.041	0.305	0.158	
		days	5.22×10^{-4}	0.002	-0.002	0.004	0.737	
		after	0.003	0.106	-0.205	0.211	0.979	
		after:days	-7.01×10^{-4}	0.002	-0.005	0.004	0.752	

Table S14. Coefficients for the moral valence metrics on the Facebook dataset. 95% confidence intervals are computed using the t distribution with model degrees of freedom. (***: $p \leq 0.001$; **: $p \leq 0.01$; *: $p \leq 0.05$; $^{+}$: $p \leq 0.1$)

Dependent Variable	Country	Term	Estimate	SE	95% CI (Lower)	95% CI (Upper)	p	Sig.
Binding moral values	IN	(Intercept)	0.293	0.021	0.252	0.333	0.000	***
		days	-0.001	4.19×10^{-4}	-0.002	-2.39×10^{-4}	0.018	*
		after	0.077	0.027	0.024	0.131	0.008	**
		after:days	9.09×10^{-4}	5.63×10^{-4}	-1.95×10^{-4}	0.002	0.128	
	SG	(Intercept)	0.287	0.051	0.188	0.386	0.000	***
		days	-4.75×10^{-4}	0.001	-0.003	0.002	0.670	
		after	0.134	0.109	-0.079	0.347	0.224	
		after:days	-0.002	0.002	-0.005	0.002	0.357	
	TH	(Intercept)	0.051	0.041	-0.030	0.132	0.225	
		days	-3.46×10^{-4}	9.48×10^{-4}	-0.002	0.002	0.716	
		after	0.022	0.053	-0.082	0.125	0.685	
		after:days	-5.25×10^{-4}	0.001	-0.003	0.002	0.629	
	TW	(Intercept)	0.406	0.086	0.237	0.576	0.000	***
		days	-7.63×10^{-4}	0.001	-0.004	0.002	0.617	
		after	-0.411	0.104	-0.615	-0.208	0.002	**
		after:days	0.002	0.002	-0.002	0.006	0.343	
Individualizing moral values	IN	(Intercept)	0.305	0.021	0.264	0.346	0.000	***
		days	-6.48×10^{-4}	4.25×10^{-4}	-0.001	1.84×10^{-4}	0.158	
		after	0.055	0.028	7.50×10^{-4}	0.110	0.063	+
		after:days	4.13×10^{-4}	5.70×10^{-4}	-7.05×10^{-4}	0.002	0.497	
	SG	(Intercept)	0.269	0.050	0.171	0.367	0.000	***
		days	-4.81×10^{-4}	0.001	-0.003	0.002	0.662	
		after	0.115	0.108	-0.097	0.327	0.293	
		after:days	-0.001	0.002	-0.005	0.002	0.457	
	TH	(Intercept)	0.055	0.040	-0.024	0.133	0.179	
		days	-1.50×10^{-4}	9.19×10^{-4}	-0.002	0.002	0.871	
		after	0.015	0.051	-0.086	0.116	0.771	
		after:days	-6.86×10^{-4}	0.001	-0.003	0.001	0.516	
	TW	(Intercept)	0.444	0.084	0.278	0.609	0.000	***
		days	0.001	0.001	-0.002	0.004	0.459	
		after	-0.448	0.101	-0.647	-0.249	0.001	***
		after:days	1.92×10^{-4}	0.002	-0.004	0.004	0.928	
Moral stereotypes	IN	(Intercept)	0.073	0.017	0.040	0.106	0.000	***
		days	7.23×10^{-5}	3.42×10^{-4}	-5.99×10^{-4}	7.43×10^{-4}	0.835	
		after	0.017	0.022	-0.027	0.061	0.458	
		after:days	1.91×10^{-4}	4.60×10^{-4}	-7.11×10^{-4}	0.001	0.683	
	SG	(Intercept)	0.099	0.040	0.020	0.178	0.017	*
		days	5.80×10^{-4}	8.71×10^{-4}	-0.001	0.002	0.508	
		after	-0.109	0.087	-0.278	0.061	0.214	
		after:days	0.002	0.001	-6.69×10^{-4}	0.005	0.144	
	TH	(Intercept)	0.064	0.015	0.035	0.093	0.000	***
		days	5.43×10^{-4}	3.38×10^{-4}	-1.20×10^{-4}	0.001	0.113	
		after	-0.030	0.019	-0.067	0.007	0.119	
		after:days	-7.84×10^{-4}	3.86×10^{-4}	-0.002	-2.66×10^{-5}	0.047	*
	TW	(Intercept)	-4.21×10^{-19}	0.003	-0.007	0.007	1.000	
		days	1.70×10^{-20}	5.97×10^{-5}	-1.17×10^{-4}	1.17×10^{-4}	1.000	
		after	0.001	0.004	-0.007	0.010	0.739	
		after:days	2.21×10^{-5}	8.53×10^{-5}	-1.45×10^{-4}	1.89×10^{-4}	0.800	

Table S15. Coefficients for the attention metrics on the Instagram dataset. 95% confidence intervals are computed using the t distribution with model degrees of freedom. (***: $p \leq 0.001$; **: $p \leq 0.01$; *: $p \leq 0.05$; $^{+}$: $p \leq 0.1$)

Dependent Variable	Country	Term	Estimate	SE	95% CI (Lower)	95% CI (Upper)	p	Sig.
Comments	IN	(Intercept)	0.148	0.003	0.142	0.153	0.000	***
		days	-3.22×10^{-5}	5.94×10^{-5}	-1.49×10^{-4}	8.42×10^{-5}	0.000	***
		after	0.011	0.005	0.002	0.020	0.000	***
		after:days	-3.94×10^{-4}	8.89×10^{-5}	-5.68×10^{-4}	-2.19×10^{-4}	0.031	*
	SG	(Intercept)	0.058	0.007	0.045	0.071	0.000	***
		days	-2.33×10^{-4}	1.11×10^{-4}	-4.51×10^{-4}	-1.56×10^{-5}	0.087	+
		after	-0.027	0.010	-0.048	-0.007	0.190	
		after:days	4.33×10^{-4}	1.77×10^{-4}	8.64×10^{-5}	7.80×10^{-4}	0.102	
	TH	(Intercept)	0.143	0.007	0.129	0.157	0.000	***
		days	7.23×10^{-4}	1.25×10^{-4}	4.77×10^{-4}	9.69×10^{-4}	0.000	***
		after	0.021	0.010	3.51×10^{-4}	0.041	0.246	
		after:days	-0.002	1.82×10^{-4}	-0.002	-0.001	0.000	***
	TW	(Intercept)	0.098	0.003	0.093	0.104	0.000	***
		days	0.001	5.23×10^{-5}	9.78×10^{-4}	0.001	0.000	***
		after	-0.003	0.004	-0.011	0.005	0.100	
		after:days	-0.002	7.16×10^{-5}	-0.002	-0.002	0.000	***
Likes	IN	(Intercept)	0.088	0.002	0.083	0.093	0.000	***
		days	-2.11×10^{-4}	5.12×10^{-5}	-3.11×10^{-4}	-1.10×10^{-4}	0.000	***
		after	0.012	0.004	0.004	0.019	0.049	*
		after:days	1.67×10^{-4}	7.67×10^{-5}	1.67×10^{-5}	3.17×10^{-4}	0.114	
	SG	(Intercept)	0.011	0.005	2.62×10^{-4}	0.022	0.311	
		days	-4.40×10^{-4}	9.26×10^{-5}	-6.22×10^{-4}	-2.59×10^{-4}	0.000	***
		after	0.023	0.009	0.006	0.039	0.020	*
		after:days	3.00×10^{-4}	1.47×10^{-4}	1.12×10^{-5}	5.89×10^{-4}	0.006	**
	TH	(Intercept)	0.111	0.007	0.099	0.124	0.000	***
		days	6.65×10^{-4}	1.15×10^{-4}	4.40×10^{-4}	8.90×10^{-4}	0.023	*
		after	-0.013	0.009	-0.031	0.006	0.071	+
		after:days	-0.001	1.67×10^{-4}	-0.001	-8.40×10^{-4}	0.000	***
	TW	(Intercept)	0.072	0.002	0.067	0.077	0.000	***
		days	8.00×10^{-4}	4.61×10^{-5}	7.10×10^{-4}	8.91×10^{-4}	0.000	***
		after	0.015	0.004	0.009	0.022	0.033	*
		after:days	-0.002	6.32×10^{-5}	-0.002	-0.002	0.000	***
Posts	IN	(Intercept)	0.001	3.67×10^{-4}	5.95×10^{-4}	0.002	0.000	***
		days	-2.67×10^{-6}	7.97×10^{-6}	-1.83×10^{-5}	1.29×10^{-5}	0.737	
		after	3.07×10^{-4}	6.07×10^{-4}	-8.84×10^{-4}	0.001	0.614	
		after:days	1.91×10^{-5}	1.19×10^{-5}	-4.23×10^{-6}	4.25×10^{-5}	0.109	
	SG	(Intercept)	0.020	0.004	0.012	0.029	0.000	***
		days	-1.66×10^{-4}	7.43×10^{-5}	-3.12×10^{-4}	-2.07×10^{-5}	0.025	*
		after	7.91×10^{-4}	0.007	-0.013	0.014	0.908	
		after:days	1.19×10^{-4}	1.18×10^{-4}	-1.13×10^{-4}	3.50×10^{-4}	0.316	
	TH	(Intercept)	0.021	0.006	0.010	0.032	0.000	***
		days	-4.67×10^{-4}	9.85×10^{-5}	-6.60×10^{-4}	-2.74×10^{-4}	0.000	***
		after	0.024	0.008	0.008	0.040	0.004	**
		after:days	1.25×10^{-4}	1.43×10^{-4}	-1.56×10^{-4}	4.05×10^{-4}	0.384	
	TW	(Intercept)	0.004	8.25×10^{-4}	0.003	0.006	0.000	***
		days	-6.91×10^{-6}	1.55×10^{-5}	-3.72×10^{-5}	2.34×10^{-5}	0.655	
		after	0.004	0.001	0.001	0.006	0.002	**
		after:days	-4.49×10^{-5}	2.12×10^{-5}	-8.64×10^{-5}	-3.35×10^{-6}	0.034	*

Table S16. Coefficients for the affective valence metrics on the Instagram dataset. 95% confidence intervals are computed using the t distribution with model degrees of freedom. (***: $p \leq 0.001$; **: $p \leq 0.01$; *: $p \leq 0.05$; $^{+}$: $p \leq 0.1$)

Dependent Variable	Country	Term	Estimate	SE	95% CI (Lower)	95% CI (Upper)	p	Sig.
Toxicity	IN	(Intercept)	0.027	7.67×10^{-4}	0.025	0.028	0.000	***
		days	-9.87×10^{-4}	1.66×10^{-5}	-0.001	-9.55×10^{-4}	0.000	***
		after	0.012	0.001	0.010	0.015	0.000	***
		after:days	0.002	2.49×10^{-5}	0.002	0.002	0.000	***
	SG	(Intercept)	0.107	0.003	0.102	0.112	0.000	***
		days	2.82×10^{-4}	4.36×10^{-5}	1.96×10^{-4}	3.67×10^{-4}	0.000	***
		after	-0.032	0.004	-0.040	-0.024	0.000	***
		after:days	1.51×10^{-4}	6.95×10^{-5}	1.44×10^{-5}	2.87×10^{-4}	0.096	+
	TH	(Intercept)	0.110	0.003	0.103	0.116	0.000	***
		days	0.001	6.08×10^{-5}	9.16×10^{-4}	0.001	0.000	***
		after	-0.003	0.005	-0.013	0.007	0.501	
		after:days	-0.001	8.84×10^{-5}	-0.001	-9.36×10^{-4}	0.000	***
	TW	(Intercept)	0.156	0.003	0.151	0.161	0.000	***
		days	2.49×10^{-4}	4.95×10^{-5}	1.52×10^{-4}	3.46×10^{-4}	0.000	***
		after	-0.023	0.004	-0.030	-0.015	0.000	***
		after:days	-4.94×10^{-4}	6.78×10^{-5}	-6.27×10^{-4}	-3.62×10^{-4}	0.000	***

Table S17. Coefficients for the moral valence metrics on the Instagram dataset. 95% confidence intervals are computed using the t distribution with model degrees of freedom. (***: $p \leq 0.001$; **: $p \leq 0.01$; *: $p \leq 0.05$; $^{+}$: $p \leq 0.1$)

Dependent Variable	Country	Term	Estimate	SE	95% CI (Lower)	95% CI (Upper)	p	Sig.
Binding moral values	IN	(Intercept)	0.011	1.97×10^{-4}	0.010	0.011	0.000	***
		days	1.09×10^{-4}	4.27×10^{-6}	1.00×10^{-4}	1.17×10^{-4}	0.000	***
		after	-0.007	3.25×10^{-4}	-0.007	-0.006	0.051	+
		after:days	-1.31×10^{-4}	6.39×10^{-6}	-1.43×10^{-4}	-1.18×10^{-4}	0.000	***
	SG	(Intercept)	0.005	3.51×10^{-4}	0.004	0.006	0.000	***
		days	4.88×10^{-5}	5.93×10^{-6}	3.71×10^{-5}	6.04×10^{-5}	0.000	***
		after	-0.004	5.49×10^{-4}	-0.006	-0.003	0.000	***
		after:days	-5.36×10^{-5}	9.45×10^{-6}	-7.21×10^{-5}	-3.50×10^{-5}	0.000	***
	TH	(Intercept)	0.009	0.003	0.003	0.015	0.013	*
		days	-2.67×10^{-4}	5.32×10^{-5}	-3.71×10^{-4}	-1.62×10^{-4}	0.000	***
		after	-0.004	0.004	-0.012	0.005	0.437	
		after:days	4.73×10^{-4}	7.73×10^{-5}	3.21×10^{-4}	6.24×10^{-4}	0.000	***
	TW	(Intercept)	0.006	1.47×10^{-4}	0.006	0.006	0.000	***
		days	8.37×10^{-5}	2.76×10^{-6}	7.82×10^{-5}	8.91×10^{-5}	0.000	***
		after	-0.006	2.13×10^{-4}	-0.006	-0.005	0.000	***
		after:days	-7.96×10^{-5}	3.78×10^{-6}	-8.70×10^{-5}	-7.21×10^{-5}	0.000	***
Individualizing moral values	IN	(Intercept)	0.024	2.78×10^{-4}	0.023	0.024	0.000	***
		days	2.08×10^{-4}	6.03×10^{-6}	1.96×10^{-4}	2.20×10^{-4}	0.000	***
		after	-0.008	4.59×10^{-4}	-0.009	-0.007	0.037	*
		after:days	-2.95×10^{-4}	9.02×10^{-6}	-3.13×10^{-4}	-2.77×10^{-4}	0.000	***
	SG	(Intercept)	0.010	0.001	0.008	0.012	0.000	***
		days	3.75×10^{-5}	1.77×10^{-5}	2.81×10^{-6}	7.23×10^{-5}	0.136	
		after	-0.007	0.002	-0.010	-0.004	0.001	***
		after:days	1.71×10^{-6}	2.82×10^{-5}	-5.36×10^{-5}	5.70×10^{-5}	0.306	
	TH	(Intercept)	0.008	0.001	0.006	0.010	0.000	***
		days	4.92×10^{-5}	1.95×10^{-5}	1.09×10^{-5}	8.75×10^{-5}	0.081	+
		after	0.009	0.002	0.006	0.012	0.003	**
		after:days	-2.43×10^{-4}	2.84×10^{-5}	-2.99×10^{-4}	-1.88×10^{-4}	0.000	***
	TW	(Intercept)	0.012	0.001	0.010	0.015	0.000	***
		days	-6.35×10^{-5}	2.59×10^{-5}	-1.14×10^{-4}	-1.27×10^{-5}	0.212	
		after	0.007	0.002	0.003	0.011	0.035	*
		after:days	-6.02×10^{-5}	3.55×10^{-5}	-1.30×10^{-4}	9.40×10^{-6}	0.151	
Moral stereotypes	IN	(Intercept)	0.086	3.52×10^{-4}	0.085	0.087	0.000	***
		days	3.65×10^{-4}	7.63×10^{-6}	3.50×10^{-4}	3.80×10^{-4}	0.000	***
		after	-0.020	5.82×10^{-4}	-0.022	-0.019	0.000	***
		after:days	-4.66×10^{-4}	1.14×10^{-5}	-4.89×10^{-4}	-4.44×10^{-4}	0.000	***
	SG	(Intercept)	0.052	0.001	0.050	0.054	0.000	***
		days	-4.24×10^{-5}	1.73×10^{-5}	-7.64×10^{-5}	-8.46×10^{-6}	0.149	
		after	-0.008	0.002	-0.011	-0.005	0.000	***
		after:days	2.04×10^{-4}	2.76×10^{-5}	1.50×10^{-4}	2.58×10^{-4}	0.000	***
	TH	(Intercept)	0.077	0.001	0.074	0.080	0.000	***
		days	5.53×10^{-4}	2.55×10^{-5}	5.03×10^{-4}	6.03×10^{-4}	0.000	***
		after	-0.015	0.002	-0.019	-0.011	0.000	***
		after:days	-6.92×10^{-4}	3.70×10^{-5}	-7.65×10^{-4}	-6.19×10^{-4}	0.000	***
	TW	(Intercept)	0.047	0.001	0.045	0.049	0.000	***
		days	3.18×10^{-4}	1.97×10^{-5}	2.79×10^{-4}	3.56×10^{-4}	0.000	***
		after	0.009	0.002	0.006	0.012	0.000	***
		after:days	-5.34×10^{-4}	2.69×10^{-5}	-5.87×10^{-4}	-4.81×10^{-4}	0.000	***

Table S18. Evidence from the Instagram dataset: Main and interaction effects of legislative change on hate speech. Coefficients report marginal effects from linear interaction models, with standard errors in parentheses and 95% confidence intervals in brackets.

	Main effect on Hate Speech	Interaction effects on Hate Speech			
	Legislative change	Media censorship	Freedom of expression	Gender equality	Social group equality
**(Main effect) Legislative change**	0.001 (0.010) [–0.019, 0.021]	-0.025*** (0.006) [–0.037, –0.013]	-0.027*** (0.006) [–0.039, –0.015]	-0.042*** (0.007) [–0.056, –0.028]	-0.029*** (0.007) [–0.043, –0.015]
**Legislative change x Print/broadcast censorship effort**		0.039*** (0.010) [0.019, 0.059]			
**Legislative change x Freedom of expression**			0.068*** (0.009) [0.050, 0.086]		
**Legislative change x Gender equality**				0.059*** (0.012) [0.035, 0.083]	
**Legislative change x Social group equality**					0.044*** (0.012) [0.020, 0.068]

Number of observations: 15,677; Number of users: 7,909; *** $p < .001$.

Table S19. Results from the Gallup dataset: Changes in support for same-sex couples in the neighborhood, with and without age and media censorship as moderators. Coefficients are marginal effects; standard errors are in parentheses and 95% confidence intervals in brackets.

	Model 1	Model 2	Model 3
**Main Effects**			
(Intercept)	15.256*	15.081*	15.600*
Gender	0.039* (0.019) [0.002, 0.076]	0.039* (0.019) [0.002, 0.076]	0.046* (0.020) [0.007, 0.085]
Education	0.014 (0.014) [-0.013, 0.041]	0.014 (0.014) [-0.013, 0.041]	0.040** (0.013) [0.015, 0.065]
Legislative change	-13.533* (6.193) [-25.66, -1.40]	-13.615* (6.196) [-25.76, -1.47]	-13.817* (6.285) [-26.14, -1.49]
Days	0.060* (0.027) [0.007, 0.113]	0.060* (0.027) [0.007, 0.113]	0.063* (0.028) [0.008, 0.118]
**Interaction Effects**			
Leg. change × Days	-0.061* (0.027) [-0.114, -0.008]	-0.061* (0.027) [-0.114, -0.008]	-0.064* (0.028) [-0.119, -0.009]
**Moderators**			
Leg. change × Age 18–34		0.172*** (0.025) [0.123, 0.221]	
Leg. change × Age 35–49		0.084*** (0.024) [0.037, 0.131]	
Leg. change × Media censorship			-0.546* (0.175) [-0.889, -0.203]
Observations	1656	1656	1656

Note: Coefficients are marginal effects. Standard errors in parentheses; 95% confidence intervals in brackets. * $p < .05$, ** $p < .01$, *** $p < .001$.

Table S20. Estimated means of perceived neighborhood safety for same-sex couples from the Gallup dataset, stratified by country, age group, and pre-/post-legislation period. The values reflect simple marginal means derived from survey responses (2017–2022). Standard errors (SE) and 95% confidence intervals (CI) are reported for each estimate. P-values indicate differences in group means between pre- and post-legislation periods. Significance indicators reflect FDR-adjusted p-values. (***: p <= 0.001; **: p <= 0.01; * p <= 0.05; + p <= 0.1)

Country	Age Group	Post-Legislation	Mean	SE	95% CI (Low)	95% CI (High)	p-value	Sig.
IN	50+	0	1.469	0.032	1.406	1.531	0.000	***
SG	50+	0	1.566	0.015	1.536	1.595	0.136	
TH	50+	0	1.406	0.015	1.378	1.435	0.645	
TW	50+	0	1.301	0.020	1.262	1.340	0.000	***
IN	50+	1	1.630	0.010	1.610	1.651	0.000	***
SG	50+	1	1.638	0.042	1.555	1.721	0.136	
TH	50+	1	1.390	0.029	1.333	1.447	0.645	
TW	50+	1	1.495	0.017	1.462	1.528	0.000	***
IN	18–34	0	1.410	0.024	1.362	1.457	0.000	***
SG	18–34	0	1.658	0.016	1.626	1.689	0.019	*
TH	18–34	0	1.686	0.015	1.656	1.715	0.300	
TW	18–34	0	1.822	0.021	1.781	1.863	0.163	
IN	18–34	1	1.620	0.008	1.605	1.636	0.000	***
SG	18–34	1	1.744	0.032	1.682	1.806	0.019	*
TH	18–34	1	1.732	0.040	1.655	1.810	0.300	
TW	18–34	1	1.858	0.014	1.830	1.885	0.163	
IN	35–49	0	1.435	0.029	1.377	1.493	0.000	***
SG	35–49	0	1.665	0.015	1.635	1.696	0.246	
TH	35–49	0	1.550	0.014	1.522	1.578	0.329	
TW	35–49	0	1.561	0.028	1.506	1.616	0.000	***
IN	35–49	1	1.600	0.009	1.581	1.618	0.000	***
SG	35–49	1	1.713	0.037	1.641	1.784	0.246	
TH	35–49	1	1.508	0.038	1.434	1.582	0.329	
TW	35–49	1	1.737	0.018	1.702	1.772	0.000	***

Table S21. Within-user coefficients (mixed-effects) for the attention metrics on the Instagram dataset. 95% confidence intervals are computed using the t distribution with model degrees of freedom. (***: $p \leq 0.001$; **: $p \leq 0.01$; *: $p \leq 0.05$; $^+$: $p \leq 0.1$)

Dependent Variable	Country	Term	Estimate	SE	95% CI (Lower)	95% CI (Upper)	p	Sig.
Comments	IN	(Intercept)	0.005	6.48×10^{-4}	0.004	0.006	0.000	***
		days	-2.23×10^{-5}	8.06×10^{-6}	-3.81×10^{-5}	-6.52×10^{-6}	0.146	
		after	8.71×10^{-4}	6.30×10^{-4}	-3.64×10^{-4}	0.002	0.268	
		after:days	1.88×10^{-5}	1.14×10^{-5}	-3.61×10^{-6}	4.11×10^{-5}	0.276	
	SG	(Intercept)	0.009	0.002	0.006	0.012	0.000	***
		days	1.25×10^{-5}	5.70×10^{-6}	1.32×10^{-6}	2.37×10^{-5}	0.124	
		after	-0.003	4.88×10^{-4}	-0.003	-0.002	0.000	***
		after:days	-1.89×10^{-6}	8.20×10^{-6}	-1.80×10^{-5}	1.42×10^{-5}	0.524	
	TH	(Intercept)	0.019	0.002	0.015	0.023	0.000	***
		days	1.06×10^{-5}	1.42×10^{-5}	-1.73×10^{-5}	3.84×10^{-5}	0.465	
		after	0.006	0.001	0.003	0.009	0.001	***
		after:days	-1.21×10^{-4}	2.82×10^{-5}	-1.76×10^{-4}	-6.55×10^{-5}	0.002	**
	TW	(Intercept)	0.011	0.002	0.006	0.015	0.000	***
		days	-8.05×10^{-6}	3.23×10^{-6}	-1.44×10^{-5}	-1.72×10^{-6}	0.304	
		after	-7.74×10^{-5}	2.68×10^{-4}	-6.03×10^{-4}	4.48×10^{-4}	0.079	+
		after:days	1.82×10^{-5}	4.93×10^{-6}	8.57×10^{-6}	2.79×10^{-5}	0.005	**
Likes	IN	(Intercept)	0.003	5.16×10^{-4}	0.002	0.004	0.000	***
		days	-4.78×10^{-6}	3.15×10^{-6}	-1.10×10^{-5}	1.39×10^{-6}	0.207	
		after	5.14×10^{-4}	2.47×10^{-4}	3.06×10^{-5}	9.98×10^{-4}	0.091	+
		after:days	7.67×10^{-6}	4.47×10^{-6}	-1.10×10^{-6}	1.64×10^{-5}	0.141	
	SG	(Intercept)	0.004	0.001	0.001	0.007	0.009	**
		days	3.74×10^{-6}	1.04×10^{-6}	1.70×10^{-6}	5.77×10^{-6}	0.038	*
		after	-5.76×10^{-4}	8.88×10^{-5}	-7.50×10^{-4}	-4.02×10^{-4}	0.000	***
		after:days	-6.60×10^{-7}	1.49×10^{-6}	-3.58×10^{-6}	2.26×10^{-6}	0.322	
	TH	(Intercept)	0.008	0.001	0.005	0.011	0.000	***
		days	-6.37×10^{-7}	1.03×10^{-5}	-2.08×10^{-5}	1.95×10^{-5}	0.742	
		after	0.005	0.001	0.003	0.007	0.002	**
		after:days	-7.56×10^{-5}	2.03×10^{-5}	-1.15×10^{-4}	-3.58×10^{-5}	0.008	**
	TW	(Intercept)	0.015	0.003	0.010	0.021	0.000	***
		days	-8.90×10^{-6}	2.86×10^{-6}	-1.45×10^{-5}	-3.30×10^{-6}	0.087	+
		after	0.001	2.37×10^{-4}	6.10×10^{-4}	0.002	0.005	**
		after:days	2.68×10^{-6}	4.36×10^{-6}	-5.87×10^{-6}	1.12×10^{-5}	0.314	
Posts	IN	(Intercept)	0.019	0.001	0.017	0.022	0.000	***
		days	-1.15×10^{-4}	1.97×10^{-5}	-1.54×10^{-4}	-7.69×10^{-5}	0.000	***
		after	-0.002	0.001	-0.005	6.95×10^{-4}	0.146	
		after:days	2.02×10^{-4}	2.69×10^{-5}	1.49×10^{-4}	2.54×10^{-4}	0.000	***
	SG	(Intercept)	0.056	0.005	0.046	0.066	0.000	***
		days	3.81×10^{-5}	9.16×10^{-5}	-1.41×10^{-4}	2.18×10^{-4}	0.685	
		after	0.028	0.008	0.012	0.044	0.001	***
		after:days	0.001	1.48×10^{-4}	8.45×10^{-4}	0.001	0.000	***
	TH	(Intercept)	0.091	0.008	0.075	0.106	0.000	***
		days	-5.87×10^{-4}	1.38×10^{-4}	-8.58×10^{-4}	-3.17×10^{-4}	0.000	***
		after	-0.044	0.012	-0.066	-0.021	0.000	***
		after:days	9.33×10^{-4}	2.18×10^{-4}	5.06×10^{-4}	0.001	0.000	***
	TW	(Intercept)	0.052	0.014	0.025	0.078	0.000	***
		days	-0.004	3.39×10^{-4}	-0.005	-0.004	0.000	***
		after	0.140	0.025	0.091	0.189	0.000	***
		after:days	0.007	5.37×10^{-4}	0.005	0.008	0.000	***

Table S22. Within-user coefficients (mixed-effects) for the affective valence metrics on the Instagram dataset. 95% confidence intervals are computed using the t distribution with model degrees of freedom. (***: $p \leq 0.001$; **: $p \leq 0.01$; *: $p \leq 0.05$; $^{+}$: $p \leq 0.1$)

Dependent Variable	Country	Term	Estimate	SE	95% CI (Lower)	95% CI (Upper)	p	Sig.
Toxicity	IN	(Intercept)	0.065	0.002	0.061	0.070	0.000	***
		days	-3.14×10^{-4}	3.35×10^{-5}	-3.80×10^{-4}	-2.49×10^{-4}	0.000	***
		after	-0.019	0.003	-0.024	-0.014	0.000	***
		after:days	9.01×10^{-4}	4.73×10^{-5}	8.08×10^{-4}	9.94×10^{-4}	0.000	***
	SG	(Intercept)	0.106	0.004	0.098	0.114	0.000	***
		days	3.23×10^{-4}	3.56×10^{-5}	2.53×10^{-4}	3.93×10^{-4}	0.000	***
		after	-0.024	0.003	-0.030	-0.018	0.000	***
		after:days	-1.71×10^{-4}	5.13×10^{-5}	-2.72×10^{-4}	-7.04×10^{-5}	0.009	**
	TH	(Intercept)	0.138	0.004	0.131	0.146	0.000	***
		days	0.002	3.25×10^{-5}	0.002	0.002	0.000	***
		after	-0.039	0.003	-0.045	-0.032	0.000	***
		after:days	-0.002	6.41×10^{-5}	-0.002	-0.002	0.000	***
	TW	(Intercept)	0.143	0.006	0.131	0.155	0.000	***
		days	-2.25×10^{-4}	2.55×10^{-5}	-2.75×10^{-4}	-1.75×10^{-4}	0.000	***
		after	0.019	0.002	0.015	0.023	0.000	***
		after:days	1.65×10^{-4}	3.90×10^{-5}	8.88×10^{-5}	2.42×10^{-4}	0.001	***

Table S23. Within-user coefficients (mixed-effects) for the moral valence metrics on the Instagram dataset. 95% confidence intervals are computed using the t distribution with model degrees of freedom. (***: $p \leq 0.001$; **: $p \leq 0.01$; *: $p \leq 0.05$; $^{+}$: $p \leq 0.1$)

Dependent Variable	Country	Term	Estimate	SE	95% CI (Lower)	95% CI (Upper)	p	Sig.
Binding moral values	IN	(Intercept)	0.006	0.001	0.004	0.009	0.000	***
		days	1.41×10^{-5}	1.24×10^{-5}	-1.02×10^{-5}	3.84×10^{-5}	0.488	
		after	-0.003	9.70×10^{-4}	-0.005	-7.62×10^{-4}	0.028	*
		after:days	-8.06×10^{-6}	1.76×10^{-5}	-4.25×10^{-5}	2.64×10^{-5}	0.545	
	SG	(Intercept)	0.005	0.001	0.002	0.007	0.011	*
		days	2.48×10^{-5}	8.32×10^{-6}	8.54×10^{-6}	4.11×10^{-5}	0.016	*
		after	-0.003	7.13×10^{-4}	-0.004	-0.001	0.039	*
		after:days	-5.12×10^{-5}	1.20×10^{-5}	-7.47×10^{-5}	-2.77×10^{-5}	0.002	**
	TH	(Intercept)	0.005	0.006	-0.006	0.016	0.426	
		days	-4.62×10^{-4}	6.43×10^{-5}	-5.88×10^{-4}	-3.36×10^{-4}	0.000	***
		after	-0.032	0.006	-0.045	-0.020	0.000	***
		after:days	0.001	1.24×10^{-4}	0.001	0.002	0.000	***
	TW	(Intercept)	0.007	0.002	0.002	0.011	0.009	**
		days	5.81×10^{-5}	5.54×10^{-6}	4.72×10^{-5}	6.89×10^{-5}	0.000	***
		after	-0.003	4.60×10^{-4}	-0.004	-0.003	0.000	***
		after:days	-5.63×10^{-5}	8.45×10^{-6}	-7.28×10^{-5}	-3.97×10^{-5}	0.000	***
Individualizing moral values	IN	(Intercept)	0.012	0.002	0.008	0.015	0.000	***
		days	-3.55×10^{-5}	2.44×10^{-5}	-8.34×10^{-5}	1.23×10^{-5}	0.251	
		after	0.001	0.002	-0.003	0.005	0.421	
		after:days	-1.92×10^{-6}	3.45×10^{-5}	-6.95×10^{-5}	6.57×10^{-5}	0.630	
	SG	(Intercept)	0.008	0.003	0.003	0.013	0.001	**
		days	-3.41×10^{-5}	2.06×10^{-5}	-7.45×10^{-5}	6.24×10^{-6}	0.145	
		after	0.004	0.002	2.47×10^{-4}	0.007	0.102	
		after:days	6.17×10^{-5}	2.97×10^{-5}	3.54×10^{-6}	1.20×10^{-4}	0.155	
	TH	(Intercept)	0.009	0.003	0.003	0.015	0.006	**
		days	-1.08×10^{-4}	1.63×10^{-5}	-1.40×10^{-4}	-7.57×10^{-5}	0.000	***
		after	-0.003	0.002	-0.006	4.24×10^{-4}	0.152	
		after:days	1.86×10^{-4}	3.24×10^{-5}	1.22×10^{-4}	2.50×10^{-4}	0.000	***
	TW	(Intercept)	0.024	0.004	0.015	0.033	0.000	***
		days	9.22×10^{-6}	2.13×10^{-5}	-3.26×10^{-5}	5.10×10^{-5}	0.362	
		after	-0.011	0.002	-0.015	-0.008	0.002	**
		after:days	1.03×10^{-4}	3.26×10^{-5}	3.87×10^{-5}	1.66×10^{-4}	0.004	**
Moral stereotypes	IN	(Intercept)	0.071	0.001	0.068	0.074	0.000	***
		days	7.83×10^{-5}	1.75×10^{-5}	4.40×10^{-5}	1.13×10^{-4}	0.004	**
		after	-0.002	0.001	-0.005	8.19×10^{-4}	0.268	
		after:days	-1.89×10^{-4}	2.47×10^{-5}	-2.37×10^{-4}	-1.40×10^{-4}	0.000	***
	SG	(Intercept)	0.053	0.002	0.050	0.057	0.000	***
		days	-1.01×10^{-4}	1.66×10^{-5}	-1.34×10^{-4}	-6.85×10^{-5}	0.000	***
		after	0.004	0.001	0.001	0.007	0.046	*
		after:days	1.92×10^{-4}	2.39×10^{-5}	1.45×10^{-4}	2.39×10^{-4}	0.000	***
	TH	(Intercept)	0.063	0.002	0.059	0.068	0.000	***
		days	2.25×10^{-4}	1.53×10^{-5}	1.95×10^{-4}	2.55×10^{-4}	0.000	***
		after	-0.006	0.002	-0.009	-0.003	0.036	*
		after:days	-3.51×10^{-4}	3.03×10^{-5}	-4.10×10^{-4}	-2.92×10^{-4}	0.000	***
	TW	(Intercept)	0.053	0.003	0.048	0.059	0.000	***
		days	2.05×10^{-4}	1.63×10^{-5}	1.73×10^{-4}	2.37×10^{-4}	0.000	***
		after	0.010	0.001	0.008	0.013	0.000	***
		after:days	-3.84×10^{-4}	2.49×10^{-5}	-4.33×10^{-4}	-3.35×10^{-4}	0.000	***

Table S24. Predictive performance of additional valence classifiers. These classifiers were excluded from our analysis due to their limited effectiveness on either in-domain data or on our secondary dataset.

	Precision	Recall	F1	Accuracy
**Validation against held-out data from the training dataset**				
LSTM-Care[18]	0.057	0.137	0.08	0.919
LSTM-Fairness	0.054	0.081	0.065	0.927
LSTM-Loyalty	0.039	0.085	0.053	0.930
LSTM-Authority	0.106	0.179	0.132	0.948
LSTM-Sanctity	0.012	0.033	0.018	0.980
LGBTQ+ hate[19]	0.822	0.827	0.806	0.826
**Validation against human annotations on the secondary dataset**				
LGBTQ+ hate	0.406	0.650	0.500	0.350

Table S25. Top Instagram hashtags and topics

**Topic**	**Hashtags**	**Number of posts**
Food	#foodie, #foodphotography, #foodblogger, #foodstagram, #healthy, #sweet, #indianfood, #eattherainbow	15,887
Travel	#travel, #travelphotography, #wanderlust, #travelgram, #exploresingapore, #indiatravelgram, #yourshot, #mypixeldiary, #lonelyplanetindia	20,109
Fashion	#fashion, #style, #ootd, #fashionblogger, #instafashion, #designer, #customized, #customizedgifts, #customizedphonecases	38,092
Photography	#photography, #photooftheday, #picoftheday, #indiapictures, #instadaily, #portrait, #beautifuldestinations, #yourshot, #memes	25,062

Table S26. Placebo analysis on the fashion dataset. (***: p <= 0.001; **: p <= 0.01; * p <= 0.05; + p <= 0.1)

	Fashion			
Feature	Country	Estimate	Standard Error	p
Attention				
Comments	IN	−0.004	0.003	
Comments	SG	−0.002	0.011	
Comments	TH	0.009	0.008	
Comments	TW	−0.032	0.014	*
Likes	IN	−95.059	37.905	*
Likes	SG	137.871	126.786	
Likes	TH	−639.371	185.299	***
Likes	TW	−57.354	77.105	
Posts	IN	0.019	0.025	
Posts	SG	−0.175	0.088	*
Posts	TH	0.232	0.053	***
Posts	TW	0.025	0.043	
Views	IN	149.980	229.977	
Views	SG	1501.433	645.452	*
Views	TH	898.454	536.956	
Views	TW	125.179	333.479	
Affective valence				
Toxicity	IN	−0.009	0.005	
Toxicity	SG	−0.017	0.015	
Toxicity	TH	−0.014	0.018	
Toxicity	TW	−0.013	0.021	
Moral valence				
Moral stereotypes	IN	−0.011	0.008	
Moral stereotypes	SG	−0.034	0.028	
Moral stereotypes	TH	0.011	0.023	
Moral stereotypes	TW	0.067	0.063	
Binding moral values	IN	0.003	0.003	
Binding moral values	SG	−0.027	0.020	
Binding moral values	TH	0.006	0.013	
Binding moral values	TW	0.010	0.007	
Individualizing moral values	IN	−0.001	0.003	
Individualizing moral values	SG	−0.009	0.016	
Individualizing moral values	TH	−0.001	0.010	
Individualizing moral values	TW	0.000	0.006	

Table S27. Placebo analysis on the food dataset. (***: p <= 0.001; **: p <= 0.01; * p <= 0.05; + p <= 0.1)

Food				
Feature	Country	Estimate	Standard Error	p
Attention				
Comments	IN	0.005	0.005	
Comments	SG	−0.011	0.006	
Comments	TH	−0.005	0.010	
Comments	TW	−0.015	0.017	
Likes	IN	−56.166	40.061	
Likes	SG	118.684	64.170	
Likes	TH	82.548	80.793	
Likes	TW	−83.252	59.189	
Posts	IN	0.070	0.017	***
Posts	SG	−0.050	0.079	
Posts	TH	−0.021	0.039	
Posts	TW	0.047	0.039	
Views	IN	9.906	253.765	
Views	SG	146.347	439.043	
Views	TH	1111.946	507.373	*
Views	TW	15.821	324.721	
Affective valence				
Toxicity	IN	−0.001	0.007	
Toxicity	SG	0.023	0.019	
Toxicity	TH	0.010	0.015	
Toxicity	TW	−0.068	0.034	*
Moral valence				
Moral stereotypes	IN	−0.044	0.015	***
Moral stereotypes	SG	−0.031	0.033	
Moral stereotypes	TH	−0.013	0.036	
Moral stereotypes	TW	−0.053	0.104	
Binding moral values	IN	−0.009	0.005	*
Binding moral values	SG	−0.012	0.009	
Binding moral values	TH	0.001	0.010	
Binding moral values	TW	0.004	0.015	
Individualizing moral values	IN	−0.004	0.004	
Individualizing moral values	SG	−0.006	0.008	
Individualizing moral values	TH	0.009	0.008	
Individualizing moral values	TW	−0.010	0.012	

Table S28. Placebo analysis on the travel dataset

Travel				
Feature	Country	Estimate	Standard Error	p
Attention				
Comments	IN	−0.006	0.002	**
Comments	SG	−0.006	0.023	
Comments	TH	0.000	0.010	
Comments	TW	−0.002	0.012	
Likes	IN	−196.037	76.520	*
Likes	SG	8.044	110.105	
Likes	TH	−1.729	97.333	
Likes	TW	−26.708	69.969	
Posts	IN	0.034	0.020	
Posts	SG	−0.121	0.050	*
Posts	TH	0.064	0.046	
Posts	TW	−0.044	0.037	
Views	IN	−93.292	154.666	
Views	SG	−35.219	395.385	
Views	TH	96.270	179.622	
views	TW	12.194	410.833	
Affective valence				
Toxicity	IN	−0.010	0.006	
Toxicity	SG	−0.067	0.035	
Toxicity	TH	−0.007	0.019	
Toxicity	TW	−0.027	0.015	
Moral valence				
Moral stereotypes	IN	0.001	0.009	
Moral stereotypes	SG	−0.117	0.049	*
Moral stereotypes	TH	−0.019	0.033	
Moral stereotypes	TW	−0.064	0.027	*
Binding moral values	IN	−0.002	0.004	
Binding moral values	SG	−0.027	0.019	
Binding moral values	TH	−0.006	0.011	
Binding moral values	TW	0.012	0.009	
Individualizing moral values	IN	−0.001	0.004	
Individualizing moral values	SG	−0.016	0.017	
Individualizing moral values	TH	−0.008	0.008	
Individualizing moral values	TW	0.001	0.009	

Table S29. Placebo analysis on the photography dataset. (***: $p <= 0.001$; **: $p <= 0.01$; * $p <= 0.05$; + $p <= 0.1$)

Photography				
Feature	Country	Estimate	Standard Error	p
Attention				
Comments	IN	−0.007	0.002	**
Comments	SG	0.001	0.024	
Comments	TH	−0.007	0.011	
Comments	TW	−0.011	0.014	
Likes	IN	−95.149	21.448	***
Likes	SG	20.832	259.239	
Likes	TH	−375.991	128.379	**
Likes	TW	5.627	63.915	
Posts	IN	0.050	0.018	**
Posts	SG	−0.043	0.068	
Posts	TH	0.004	0.031	
Posts	TW	0.014	0.039	
Views	IN	−50.942	147.348	
Views	SG	1904.622	1001.167	
Views	TH	443.128	434.796	
Views	TW	−61.484	145.806	
Affective valence				
Toxicity	IN	−0.014	0.005	**
Toxicity	SG	0.002	0.021	
Toxicity	TH	−0.032	0.018	
Toxicity	TW	−0.004	0.017	
Moral valence				
Moral stereotypes	IN	−0.007	0.005	***
Moral stereotypes	SG	−0.083	0.075	
Moral stereotypes	TH	0.017	0.026	
Moral stereotypes	TW	−0.053	0.058	
Binding moral values	IN	0.001	0.004	
Binding moral values	SG	−0.012	0.020	
Binding moral values	TH	−0.022	0.010	*
Binding moral values	TW	0.023	0.015	
Individualizing moral values	IN	−0.001	0.004	
Individualizing moral values	SG	−0.009	0.017	
Individualizing moral values	TH	−0.011	0.007	
Individualizing moral values	TW	0.003	0.016	

References

1. Grootendorst, M. Bertopic: Neural topic modeling with a class-based tf-idf procedure. *arXiv preprint arXiv:2203.05794* (2022).
2. Baucom, E. An exploration into archival descriptions of lgbtq materials. *The Am. Arch.* **81**, 65–83 (2018).
3. Zhang, B., You, Y., He, G., Yan, F. & Wang, S. Searching for the rainbow connection: Regional development and lgbt communities in china. *J. Contemp. China* 1–23 (2022).
4. Fan, A. *et al.* Beyond english-centric multilingual machine translation. *The J. Mach. Learn. Res.* **22**, 4839–4886 (2021).
5. Chen, G. *et al.* Zero-shot cross-lingual transfer of neural machine translation with multilingual pretrained encoders. *arXiv preprint arXiv:2104.08757* (2021).
6. Bouscarrat, L., Bonnefoy, A., Capponi, C. & Ramisch, C. Multilingual enrichment of disease biomedical ontologies. *arXiv preprint arXiv:2004.03181* (2020).
7. Gehman, S., Gururangan, S., Sap, M., Choi, Y. & Smith, N. A. Realtoxicityprompts: Evaluating neural toxic degeneration in language models. In *Findings of the Association for Computational Linguistics: EMNLP 2020*, 3356–3369 (2020).
8. Pavlopoulos, J., Thain, N., Dixon, L. & Androutsopoulos, I. Convai at semeval-2019 task 6: Offensive language identification and categorization with perspective and bert. In *Proceedings of the 13th international Workshop on Semantic Evaluation*, 571–576 (2019).
9. Welbl, J. *et al.* Challenges in detoxifying language models. *arXiv preprint arXiv:2109.07445* (2021).
10. Rosenblatt, L., Piedras, L. & Wilkins, J. Critical perspectives: A benchmark revealing pitfalls in perspectiveapi. In *Proceedings of the Second Workshop on NLP for Positive Impact (NLP4PI)*, 15–24 (2022).
11. Hede, A., Agarwal, O., Lu, L., Mutz, D. C. & Nenkova, A. From toxicity in online comments to incivility in american news: Proceed with caution. *arXiv preprint arXiv:2102.03671* (2021).
12. Zhang, J. *et al.* Conversations gone awry: Detecting early signs of conversational failure. *arXiv preprint arXiv:1805.05345* (2018).
13. Nicolas, G., Bai, X. & Fiske, S. T. Comprehensive stereotype content dictionaries using a semi-automated method. *Eur. J. Soc. Psychol.* **51**, 178–196 (2021).
14. Platten, S., Haji, R. & Boyd, R. L. Dehumanizing representations of muslims in relation to 9/11: A new york times analysis. [Poster presentation] (2019).
15. Platten, S., Haji, R. & Boyd, R. L. Humanizing and dehumanizing themes of muslims surrounding 9/11: Computerized language analysis. [Poster presentation] (2020).
16. Danescu-Niculescu-Mizil, C., Sudhof, M., Jurafsky, D., Leskovec, J. & Potts, C. A computational approach to politeness with application to social factors. In *Proceedings of the 51st Annual Meeting of the Association for Computational Linguistics (Volume 1: Long Papers)*, 250–259 (2013).
17. Hopp, F. R., Fisher, J. T., Cornell, D., Huskey, R. & Weber, R. The extended moral foundations dictionary (emfd): Development and applications of a crowd-sourced approach to extracting moral intuitions from text. *Behav. research methods* **53**, 232–246 (2021).
18. Atari, M. *et al.* Morally homogeneous networks and radicalism. *Soc. Psychol. Pers. Sci.* **13**, 999–1009 (2022).
19. Dacon, J., Shomer, H., Crum-Dacon, S. & Tang, J. Detecting harmful online conversational content towards lgbtqia+ individuals. In *Queer in AI Workshop at NAACL* (2022).
