# Supplementary material for: Decriminalizing same-sex relationships and its impact on social media discussions in Asia
Source: Nat Hum Behav. 2026 Jun 10;10(8):1474–86. doi: 10.1038/s41562-026-02485-6 (PMC13485699; doi:10.1038/s41562-026-02485-6)
Supplement: Supplementary file 2 [file 41562_2026_2485_MOESM2_ESM.pdf]

nature portfolio

Corresponding author(s): *Double-anonymous peer review submissions: write DAPR and your manuscript number here instead of author names.*

Last updated by author(s): *YYYY-MM-DD*

Reporting Summary

Nature Portfolio wishes to improve the reproducibility of the work that we publish. This form provides structure for consistency and transparency in reporting. For further information on Nature Portfolio policies, see our Editorial Policies and the Editorial Policy Checklist.

Statistics

For all statistical analyses, confirm that the following items are present in the figure legend, table legend, main text, or Methods section.

n/a	Confirmed	
☐	☒	The exact sample size (*n*) for each experimental group/condition, given as a discrete number and unit of measurement
☐	☒	A statement on whether measurements were taken from distinct samples or whether the same sample was measured repeatedly
☐	☒	The statistical test(s) used AND whether they are one- or two-sided<br>*Only common tests should be described solely by name; describe more complex techniques in the Methods section.*
☐	☒	A description of all covariates tested
☐	☒	A description of any assumptions or corrections, such as tests of normality and adjustment for multiple comparisons
☐	☒	A full description of the statistical parameters including central tendency (e.g. means) or other basic estimates (e.g. regression coefficient) AND variation (e.g. standard deviation) or associated estimates of uncertainty (e.g. confidence intervals)
☒	☐	For null hypothesis testing, the test statistic (e.g. *F*, *t*, *r*) with confidence intervals, effect sizes, degrees of freedom and *P* value noted<br>*Give P values as exact values whenever suitable.*
☒	☐	For Bayesian analysis, information on the choice of priors and Markov chain Monte Carlo settings
☒	☐	For hierarchical and complex designs, identification of the appropriate level for tests and full reporting of outcomes
☐	☒	Estimates of effect sizes (e.g. Cohen's *d*, Pearson's *r*), indicating how they were calculated

Our web collection on statistics for biologists contains articles on many of the points above.

Software and code

Policy information about availability of computer code

Data collection	The CrowdTangle dashboard (https://github.com/CrowdTangle/API, version Dec 12, 2022) was used to collect the Instagram and Facebook data. No software was used to collect the Gallup data.
Data analysis	The Python package regressors (version 0.0.3) was used to prepare Figure 1. The R packages lmerTest (version 3.1.3) were used for Figure 2 and S1, Tables S10-S14. The interactions package (version 1.2.0) was used for Figure 3 and S2.

For manuscripts utilizing custom algorithms or software that are central to the research but not yet described in published literature, software must be made available to editors and reviewers. We strongly encourage code deposition in a community repository (e.g. GitHub). See the Nature Portfolio guidelines for submitting code & software for further information.

Data

Policy information about availability of data

All manuscripts must include a data availability statement. This statement should provide the following information, where applicable:

- Accession codes, unique identifiers, or web links for publicly available datasets
- A description of any restrictions on data availability
- For clinical datasets or third party data, please ensure that the statement adheres to our policy

The Facebook and Instagram datasets were collected using Meta's Content Library (formerly CrowdTangle), in accordance with Meta's platform policies and data access requirements. These data are not redistributed by the authors. Readers may apply for access to Meta data via the Meta Content Library and Researcher

Platform at: https://transparency.meta.com/researchtools/meta-content-library.The Gallup World Poll data were accessed through an institutional subscription. Researchers seeking to use these data must obtain access directly from Gallup through their licensing process.

Research involving human participants, their data, or biological material

Policy information about studies with human participants or human data. See also policy information about sex, gender (identity/presentation), and sexual orientation and race, ethnicity and racism.

Reporting on sex and gender	This study analyzes secondary survey data collected by the Gallup World Poll. Sex was self-reported by respondents and recorded as binary (male/female) in the source dataset. Analyses treat sex as a demographic characteristic rather than as a causal variable. Sex-disaggregated descriptive statistics are reported where relevant. No inference is made regarding gender identity beyond the categories available in the source data.
Reporting on race, ethnicity, or other socially relevant groupings	Race and ethnicity were not measured in a harmonized or comparable manner across countries in the Gallup World Poll and therefore were not included in the present analyses. Instead, national context was used as the primary unit of comparison, consistent with the cross-national design of the survey. Socioeconomic status was captured using a relative income measure (country-specific income quintiles), which reflects respondents' position within their national income distribution rather than absolute income levels. Education was measured using harmonized categories provided by Gallup (primary, secondary, tertiary). These variables were used as covariates to account for structural differences across respondents.
Population characteristics	The study analyzes survey responses from 46,200 adult respondents across four countries (India, Singapore, Taiwan, and Thailand), collected between 2017 and 2022. Samples are nationally representative within each country. Respondents span a wide adult age range (18–65+), with broadly balanced gender distributions across countries. Education and income were measured using harmonized, country-relative scales. Detailed demographic distributions by country (age, gender, education, and income) are reported in Table S7 of the Supplementary Materials.
Recruitment	All data were obtained from the Gallup World Poll, which uses probability-based sampling methods designed to produce nationally representative samples of the adult population in each country. Recruitment procedures, informed consent, and survey administration were conducted by Gallup in accordance with their standard protocols. The authors had no role in participant recruitment, consent procedures, or data collection. As such, potential self-selection or nonresponse biases reflect those inherent to large-scale survey research and are discussed in the interpretation of findings.
Ethics oversight	This study involved secondary analysis of fully anonymized, publicly available survey data. No new data were collected, and no identifiable private information was accessed. In accordance with institutional and journal guidelines, this research does not constitute human subjects research requiring additional ethics review. The original data collection procedures were reviewed and approved by Gallup's internal ethics and compliance processes.

Note that full information on the approval of the study protocol must also be provided in the manuscript.

Field-specific reporting

Please select the one below that is the best fit for your research. If you are not sure, read the appropriate sections before making your selection.

☐ Life sciences ☒ Behavioural & social sciences ☐ Ecological, evolutionary & environmental sciences

For a reference copy of the document with all sections, see nature.com/documents/nr-reporting-summary-flat.pdf

Behavioural & social sciences study design

All studies must disclose on these points even when the disclosure is negative.

Study description	This study employed a quasi-experimental, multi-method design to examine the impact of LGBTQ+-related legal reforms on public discourse across social media and survey data. Using an interrupted time series (ITS) framework, we analyzed over 136,000 Facebook posts and 65,000 Instagram posts related to LGBTQ+ issues in India, Taiwan, Thailand, and Singapore before and after key legislative changes. Supplementary analysis used nationally representative survey data from the Gallup World Poll to compare trends in perceived community acceptance. We triangulated attention, affective valence, and moral framing across platforms, integrating automated text analysis and contextual indicators (e.g., civil liberties, social equality) to examine how structural conditions shape responses to reform.
Research sample	We analyzed social media posts from Facebook and Instagram, as well as survey data from the Gallup World Poll, focusing on four Asian countries: India, Taiwan, Thailand, and Singapore. Facebook data consisted of 136,255 posts published by major national news outlets, providing a high-reach measure of institutional media discourse. Instagram posts (N = 65,969) were collected using country-specific hashtags and filtered to retain only those relevant to LGBTQ+ individuals and topics, thereby capturing user-driven expressions of identity and solidarity. These platforms were selected due to their popularity in the region and their respective affordances for institutional communication versus individual expression. To complement this digital trace data, we incorporated individual-level survey data from the Gallup World Poll, filtered to responses from the same four countries and in the time period of interest (N = 46.200), to compare shifts in public discourse with reported perceptions of LGBTQ+ acceptance. Together, these data sources offer a multi-perspective, ecologically valid sample for examining how LGBTQ+ discourse evolves in response to legal change across diverse socio-political contexts.
Sampling strategy	The complete dataset was used.

Data collection	The Facebook and Instagram datasets were collected using the CrowdTangle dashboard, which provided access to public content from verified and high-reach accounts in accordance with Meta's terms of service. As of August 2024, the CrowdTangle dashboard is no longer available. Facebook data were retrieved based on a curated list of national news outlets for each country, while Instagram data were collected using country-specific keywords and hashtags to identify posts relevant to LGBTQ+ topics. This process was automated and conducted without manual intervention, ensuring that data collection was blind to the hypotheses and statistical models of the study.<br><br>The Gallup World Poll dataset was accessed through a university subscription and includes nationally representative survey responses from randomly sampled individuals, collected using standardized instruments and validated translation protocols. The collection of this survey data was also independent of the current research design. The Facebook and Instagram datasets can be made available to accredited researchers under a non-disclosure agreement, while access to the Gallup dataset requires an institutional subscription. All preprocessing code, model parameters, and derived data used for analysis have been deposited on the Open Science Framework (OSF) at https://osf.io/3h478/.
Timing	Data collection started on January 15, 2023 and ended on March 31, 2023.
Data exclusions	No participants were excluded based on demographic characteristics, responses, or outcomes of interest. Analyses were conducted on all available observations from the Gallup World Poll that met the inclusion criteria of (i) being collected in one of the four focal countries (India, Singapore, Taiwan, and Thailand) and (ii) falling within the specified temporal window surrounding the legislative events under study.Observations with missing values on key variables (e.g., outcome measures or covariates) were excluded on a listwise basis for the specific models in which those variables were required. This resulted in minor and outcome-specific variation in analytic sample sizes, which is reported in the corresponding tables. No additional exclusion criteria were applied, and no observations were removed based on outcome values or post hoc considerations.
Non-participation	Non-participation was determined by the original survey design of the Gallup World Poll. Individuals who declined to participate, were unreachable, or did not complete the survey were not included in the dataset provided to the authors. Because this study relies on secondary analysis of anonymized, cross-sectional survey data, the research team did not have access to information about non-respondents or the ability to influence participation rates. Potential non-response bias is therefore inherent to the source data and reflects standard limitations of large-scale population surveys.
Randomization	Randomization was not applicable to this study as the complete dataset was used.

Reporting for specific materials, systems and methods

We require information from authors about some types of materials, experimental systems and methods used in many studies. Here, indicate whether each material, system or method listed is relevant to your study. If you are not sure if a list item applies to your research, read the appropriate section before selecting a response.

Materials & experimental systems

n/a	Involved in the study
☒	☐ Antibodies
☒	☐ Eukaryotic cell lines
☒	☐ Palaeontology and archaeology
☒	☐ Animals and other organisms
☒	☐ Clinical data
☒	☐ Dual use research of concern
☒	☐ Plants

Methods

n/a	Involved in the study
☒	☐ ChIP-seq
☒	☐ Flow cytometry
☒	☐ MRI-based neuroimaging

Plants

Seed stocks	*Report on the source of all seed stocks or other plant material used. If applicable, state the seed stock centre and catalogue number. If plant specimens were collected from the field, describe the collection location, date and sampling procedures.*
Novel plant genotypes	*Describe the methods by which all novel plant genotypes were produced. This includes those generated by transgenic approaches, gene editing, chemical/radiation-based mutagenesis and hybridization. For transgenic lines, describe the transformation method, the number of independent lines analyzed and the generation upon which experiments were performed. For gene-edited lines, describe the editor used, the endogenous sequence targeted for editing, the targeting guide RNA sequence (if applicable) and how the editor was applied.*
Authentication	*Describe any authentication procedures for each seed stock used or novel genotype generated. Describe any experiments used to assess the effect of a mutation and, where applicable, how potential secondary effects (e.g. second site T-DNA insertions, mosiacism, off-target gene editing) were examined.*
