# Supplementary material for: Decriminalizing same-sex relationships and its impact on social media discussions in Asia
Source: Nat Hum Behav. 2026 Jun 10;10(8):1474–86. doi: 10.1038/s41562-026-02485-6 (PMC13485699; doi:10.1038/s41562-026-02485-6)
Supplement: Supplementary file 3 [file 41562_2026_2485_MOESM3_ESM.pdf]

nature portfolio

Peer Review File

Decriminalizing same-sex relationships and its impact on social media discussions in Asia

Corresponding Author: Professor Kokil Jaidka

Version 0:

Decision Letter:

15th August 2024

Dear Dr Jaidka,

Thank you once again for your manuscript, entitled "Decriminalizing Homosexuality and Its Impact on Social Media Discussions in Asia," and for your patience during the peer review process.

Your manuscript has now been evaluated by 3 reviewers, whose comments are included at the end of this letter. In the light of their advice, I regret that we cannot offer to publish your manuscript in Nature Human Behaviour.

While the reviewers find your work of some interest, they raise concerns about the theoretical advance your findings represent over earlier work, and over the strength of the novel conclusions that can be drawn at this stage. We feel that these reservations are sufficiently important as to preclude publication of this work in Nature Human Behaviour.

I am sorry that we cannot be more positive on this occasion but hope that you will find our reviewers' comments helpful when preparing your paper for submission elsewhere.

Sincerely,
[redacted]

[redacted]

Reviewers' Comments:

Reviewer #1:
Remarks to the Author:
The authors engage in an analysis of social media data to assess the impact of legislative changes in four Asian countries on societal attitudes towards LGBTQ+ issues. The authors transparency in discussing their data and methods should be applauded, and they share some potentially illuminating findings about the relationship between social media, legislation, and public discourse. However, there are several issues that should be addressed before this manuscript is ready for publication, as well as a few minor points that need to be resolved.

First, the authors claim that the results of this study reflect public opinion on LGBTQ+ topics in India, Taiwan, Thailand, and Singapore. The Instagram and Facebook posts were limited to discourse related to LGBTQ+ related legislation in these nations reported by news outlets in these nations, but I did not see any indication that authors were able to assess the location of users who responded to these news stories. Is it possible that the sample includes individuals living in other countries? If so, the authors need to rework their discussion to clarify that this is an assessment of global attitudes rather than reporting findings as “discourse in India [Thailand, Taiwan, or Singapore]”.

Second, the authors effectively situated their research within moral foundation theory. However, they did not provide any theoretical basis for the main purpose of this study—assessing the impact of legislation on social attitudes towards LGBTQ+ individuals. There are numerous theoretical approaches to this topic and without providing the reader with information about which theoretical frameworks are driving their assessment of the relationship between state-level policies and cultural

norms, the study lacks any theoretical significance.

Third, given the inconsistency in significance observed between Facebook and Instagram around moral stereotypes, individualizing moral values, binding moral values, and toxicity. There are many potential explanations for the inconsistencies found between the two social media platforms (age is likely a contributing factor (active Facebook users tend to be older than Instagram users) but the authors must address this in more detail and include it in the limitations section. Furthermore, the authors also make strong claims that may be overstated given this inconsistency, for example "Our findings support the role played by national institutions in ameliorating the public acrimony around marginalized groups and potentially mending inter-group relations. We found that after homosexuality was decriminalized in four Asian countries, public discourse was subdued but less affectively polarized and morally charged than before."

The three issues mentioned above must be addressed before the study is fit for publication as they represent significant weaknesses in the manuscript as currently written. That stated, I found that the most compelling finding (the third related to the secondary data) was underrepresented and warranted further exploration.

Minor Issues/Comments:

The decrease in LGBTQ+ discourse could also be related to the perception that legalization was the only discrimination that LGBTQ+ people face. This has been discussed in relation to marriage equality in the U.S. Queer theorists have argued that these legalization efforts are attempts to assimilate LGBTQ people into heteronormativity, thereby promoting an "acceptable" level of queerness while still perpetuating cultural norms that marginalize LGBTQ+ people.

Use same-sex, sexual minority, or LGBTQ instead of homosexual and homosexuality. The term homosexuality is linked to a history of medicalization and stigmatization.

"region-level" should be capitalized in the first paragraph on page 2

Reviewer #2:
Remarks to the Author:
Thank you for inviting me to review this article. While the research questions this study sought to explore are highly intriguing and important, I found the article to contain several weaknesses and confusing content. I would suggest thorough clarification and revisions to enhance readability, logical coherence, and the clear presentation of results. My specific comments are provided below.

1. The question about the moderation effect of democratic indicators on how individuals appraise LGBTQ+ topics seems disconnected from the preceding statements. Could you clarify the importance of identifying these potential moderation effects? Did you aim to examine the robustness of relationships between variables of interest or highlight contextual variabilities in the findings? Providing clear rationales and theoretical bases for examining moderation effects would be beneficial. Additionally, the term "democratic indicators" may be confusing as the moderators mentioned appear to be methodological (sampling, measurement, locations). In the results section, the interaction effects tested involve social indicators (such as press freedom, freedom of speech, and social group equities), which are presented as contextual moderators in the methods section, leading to confusion.
2. The relevance of MFT to this study is not very clear. While it could provide insights into characterizing the moral reasoning underlying public discourse, the study's focus extends beyond this to examine the impact of legal changes on public discourse. It may be beneficial to incorporate other theories to support your main research questions and hypotheses. Additionally, the theories are not adequately elaborated for readers. As someone encountering this theory for the first time, I found it challenging to comprehend its meaning, application, and relevance to the study. While one might intuitively expect public discourse on LGBTQ individuals to be categorized as friendly/accepting or exclusionary/hostile, MFT offers an alternative approach to assessing discourse. However, the justification for its appropriateness in this study is not well-established.
3. I believe the introduction section could be condensed to better prepare readers to understand the research questions and hypotheses. Currently, the section covers multiple discussion threads, but the coherence and relevance to the study are not sufficiently strong. For instance, the initial discussion about the design of social media platforms leading to LGBTQ individuals being hypervigilant online and becoming targets of harassment, as well as the mention of ally activism potentially causing victimization, are not directly related to the core questions of the study. The study aims to investigate the impact of societal acceptance and legal changes on discourses about LGBTQ individuals on social media. Similarly, the paragraph on page 2 that starts with 'this study posits that a comprehensive understanding of social media's impact on society...' seems to confuse the study's focus. I recommend that the authors maintain a clear focus on the research objectives throughout the article.
4. Concerning the tertiary dataset, it is debatable to utilize the question of whether respondents perceived their community as safe for LGBT+ individuals as a proxy for public attitudes towards LGBTQ+. This item reflects one's perception of the environment rather than their direct attitudes towards this population. While it may be correlated with related measures, it does not represent public attitudes towards LGBTQ+ individuals.
5. In the paragraph preceding the limitations section, the authors wrote, "there is an increasing need to recognize LGBTQ+ individuals as a gender minority group." I believe the authors may be conflating sexual minority with gender minority, despite the overlap between them. The relationship between the implications proposed here and the study findings is unclear.
6. In the limitations the authors acknowledged that this study did not distinguish between stances towards LGBTQ+ people

and LGBTQ+ self-expression. Could you elaborate on this point further?

Reviewer #3:
Remarks to the Author:
The paper titled, "Decriminalizing Homosexuality and Its Impact on Social Media Discussions in Asia" explores four Asian countries that have decriminalized homosexuality via a study of causal
effective legislative change on public attitudes and acceptance towards LGBTQ+ community and intersectional topics thereof. The three multifaceted questions: 1) What are the effects of a legislative change decriminalizing homosexuality, on the (a) news coverage and (b) social media attention to LGBTQ+ topics?; 2) What is the impact of the legal acceptance of homosexual relationships on the (a) negative affect and (b) moral stereotypes in the LGBTQ+ discourse on social media?; and 3)What is the moderation effect of democratic indicators on how individuals appraise LGBTQ+ topics? are centered from a social and psychological lens centered in a legislative perspective. Looking at something from a legislative lens typically means examining it with a focus on how laws, regulations, and policies apply or could apply to the subject at hand. It involves considering the legal implications, constraints, and opportunities that legislation or potential legislation might bring to the issue or topic under discussion. This perspective is particularly important when discussing how laws can shape behaviors, industries, governance structures, and societal norms.

There were a couple typos/grammatical errors and capitalization issues in the article, such as " region-level" on page 2. Please go over the paper, and add in correction punctuations as well as some sentences are run-on sentences and need to be shorter (more concise).

The datasets constitutes 136,255 multilingual posts from national news outlets in India, Taiwan, Thailand, and Singapore on Facebook between 2016-2023, of which 86.87% were in English, 7.6 million Instagram posts between 2016-2023, of which 74.37% were in English, and 46,200 survey responses from the Gallup World Poll between 2017-2022. Although these datasets are of different distributions, the authors claimed that it enabled a focused understanding and comparison of online vs. offline attitudes towards LGBTQ+ people in four Asian countries namely, India, Taiwan, Thailand, and Singapore. The background does not include or mention much of online discussions, panels, talk page edits, etc. such as Reddit which often contain harmful conversational content i.e., hate speech, death threats and offensive language, especially towards certain demographic groups compared to Instagram's censored or moderated comment section and posting policy, which can most certainly play a crucial role in the results. This study claims to attain a comprehensive understanding of social media’s impact on society by considering platforms that have may not display unfiltered comments from individuals who may oppose, criticize, or possess contradictory feelings, beliefs, or motivations towards the LGBTQ+ communities 9Twitter, Reddit), yet posits a NLP approach that focuses on evaluating the effects of legislative changes on media coverage and social media discourse regarding LGBTQ+ topics, examining the dynamics of negative affect and moral stereotypes. The claim, "However, prior work lacks a
comprehensive measurement of LGBTQ+ discourse, spanning different modalities and affective dimensions, and the effect of external interventions." is a very large claim with little citation to claim this. For example the following works:
- Experimental Design Considerations for Limiting Psychological Distress presented by Mary Smart (UCSD)

- Decentering Imputation: Fair Learning at the Margins of Demographics presented by Evan Dong (Brown University)

- Iterative Value-Aware Model Learning on the Value Improvement Path presented by Claas Voelcker (University of Toronto)

- Molecular Fingerprints Are a Simple Yet Effective Solution to the Drug–Drug Interaction Problem presented by Yanan Long (University of Chicago)

- From Pedestrian Detection to Crosswalk Estimation: An EM Algorithm, Analysis, and Evaluations on Diverse Datasets presented by Ross Greer (UCSD)

- Detecting Harmful Online Conversational Content towards LGBTQIA+ Individuals. Jamell Dacon, Harry Shomer, Shaylynn Crum-Dacon, Jiliang Tang

- Outed by an Algorithm: A Study on Facebook’s Friends Recommendation System for Queer, Trans and Gender-Non-Conforming Users. Jack Chang

- Towards WinoQueer: Developing a Benchmark for Anti-Queer Bias in Large Language Models. Virginia K. Felkner, Ho-Chun Herbert Chang, Eugene Jang, Jonathan May

- Use of a Stylometric Map-Based Corpus for Tracking Individual Variation in Relation to Gender and Sex. Theodore Daniel Manning, Harleigh Niyu, Alejandro Jorge Napolitano Jawerbaum, Patrick Juola

- Overview of STEM Science as Process, Data, Material, and Method Named Entities. Jennifer D'Souza

- CNSC: Czech news article dataset for classification of originating source and its credibility. Matyáš Bohácek

- Tackling Gender Microaggressions in Hindi. Vishakha Agrawal

How do you define the term "morality" in your study given "Morality refers to the use of language or appeal to moral principles, particularly in discussions involving
LGBTQ+ topics. This includes advocating for ethical treatment, human rights, and equality, but it may also condemn LGBTQ+ individuals". Ethical Clarity: It can refer to the ethical or moral dimensions of how something is defined. For example, in discussions about policies or actions (in this article), a moral definition would emphasize the ethical principles and values that guide how something is understood or categorized but from whose perspective, the LGBTQ+ community in the 4 Asian countries,

Defining Morality: It can also imply defining what is considered moral or ethical in a particular context. This involves specifying the principles, standards, or norms that are deemed morally acceptable or desirable within a society, culture, or philosophical framework.

In both cases, "moral definition" involves clarifying the ethical implications, considerations, or criteria that shape our understanding and judgment of a given subject or situation.

There are NLP models that are trained on toxicity that can also determine the level of toxicity of the english comments and not solely rely on the methods used in this paper namely, pairwise Pearson correlation and ridge regression analysis. This paper does not do a thorough analysis of existing work that has been done and fails to conduct a thorough analysis using state of the art technologies, thus raising several questions about the findings in terms of correlation, harmful language detection models, specifically for language contains threats, obscene, etc. Also, when taking into consideration "Swear words" it does not account for sarcasm.

Finally, analyzing Pearson correlations (r) between language-based measurements from Instagram posts and the likelihood that a social media post is about LGBTQ+ topics has its advantages and limitations. Although the paper may display its advantages, limitations of this technique need to be discussed:
1) Causation vs. Correlation: Correlation does not imply causation. A significant correlation between language-based measurements and LGBTQ+ content does not necessarily mean that language use causes the likelihood of LGBTQ+ topics or vice versa.

2) Contextual Understanding: Correlations may not capture the nuanced context or intent behind language use in social media posts, as previously mentioned . For instance, certain words or phrases might be used sarcastically, ironically, or in contexts unrelated to LGBTQ+ topics.

3) Sample Representativeness: The findings are limited by the representativeness of the sample. Instagram users who post about LGBTQ+ topics may not be representative of all social media users or all LGBTQ+ individuals.

4) Language and Culture Variations: The effectiveness of language-based measurements can vary across different languages, cultures, and demographics, which may affect the generalizability of the findings.

5) Data Quality: The quality and reliability of language-based measurements (such as sentiment analysis, keyword analysis, hashtags, etc.) can influence the validity of correlations. Inaccurate or biased measurements can lead to misleading results.

***

**Following suitable revisions, you may want to consider transferring your manuscript. Although we cannot publish your paper, it may be appropriate for another journal in the Nature Portfolio. If you wish to explore the journals and transfer your manuscript please use our manuscript transfer portal. You will not have to re-supply manuscript metadata and files, unless you wish to make modifications. For more information, please see our manuscript transfer FAQ page.

Version 1:

Decision Letter:

Dear Dr Jaidka,

Thank you for your correspondence asking us to reconsider our decision on your Article, "Decriminalizing Homosexuality and Its Impact on Social Media Discussions in Asia". After careful consideration we have decided that we would be willing to consider a revised version of your manuscript.

Along with your revised manuscript, you should also submit a separate point-by-point response to all of the concerns raised by the referees, in each case describing what changes have been made to the manuscript or, alternatively, if no action has

been taken, providing a compelling argument for why that is the case. If we feel that a substantial attempt has been made to address the referees' comments, this response will be sent back to the referees - along with the revised manuscript - so that they can judge whether their concerns have been addressed satisfactorily or otherwise.

I should stress, however, that we would be reluctant to trouble our referees again unless we thought that their comments had been addressed in full.

When revising your paper:

- ensure it complies with our format requirements as set out in our Guide to Authors.

- state in a cover note the length of the text, methods and figure legends; the number of references and the number of display items.

Please ensure that all correspondence is marked with your Nature Human Behaviour reference number in the subject line.

Please use the following link to submit your revised manuscript:

Link Redacted

** This url links to your confidential home page and associated information about manuscripts you may have submitted or be reviewing for us. If you wish to forward this email to co-authors, please delete the link to your homepage first **

We hope to receive your revised paper within four weeks. If you cannot send it within this time, please let us know so that we can close your file. In this event, we will still be happy to reconsider your paper at a later date so long as nothing similar has been accepted for publication at Nature Human Behaviour or published elsewhere in the meantime. Should you miss the four-week deadline and your paper is eventually published, the received date will be that of the revised, not the original, version.

I look forward to hearing from you soon.

Best regards,

[redacted]
Nature Human Behaviour

Version 2:

Decision Letter:

Our ref: NATHUMBEHAV-24030861B

24th February 2025

Dear Dr. Jaidka,

Thank you for submitting your revised manuscript "Decriminalizing same-sex relationships and its impact on social media discussions in Asia" (NATHUMBEHAV-24030861B). It has now been seen by two original and a new referees and their comments are below. As you can see, the reviewers find that the paper has improved in revision. We will therefore be happy in principle to publish it in Nature Human Behaviour, pending minor revisions to satisfy the referees' final requests and to comply with our editorial and formatting guidelines.

We are now performing detailed checks on your paper and will send you a checklist detailing our editorial and formatting requirements in the following weeks. Please do not upload the final materials and make any revisions until you receive this additional information from us.

Please do not hesitate to contact me if you have any questions.

Sincerely,

[redacted]
Nature Human Behaviour

Reviewer #1 (Remarks to the Author):

I appreciate the opportunity to once again review this manuscript. The author(s) have made significant attempts to address comments and issues raised by reviewers. In the following review, I will address the author's responses to issues raised by reviewer 1, as well as outstanding issues that must be tackled prior to publication.
Author's Response to Reviewer 1:
Comments 2-3:
Thank you for further clarifying the lack of location data as a limitation of the study. I find the authors' response justifies this methodological choice. The authors have also modified sentences that implied the discourse was limited to those residing in the countries of focus in this research.
Comment 4:
The author'(s) choice to frame the findings of this research using structuration theory has dramatically improved the manuscript and highlights the empirical contributions of this work.
That said, I would suggest that the author'(s) elaborate on how their findings align with saturation theory in the conclusion. For example, page 8 line 219 states:
"The results show that negative affect increased significantly in countries with reduced social equality, such as India. At the same time, it decreased in countries with higher press freedom and greater freedom of expression, such as Taiwan. These findings align with the expectations of the structuration theory as they corroborate the role of social norms, political institutions, and cultural attitudes in explaining societal responses to the legislative change."
The author(s) correctly claims that the results from this research support structuration theory, but it should be made explicitly clear to the reader WHY these results align with this theory. Also, how does this study add to structuration theory? What is the author(s) doing that has not been done in other research using this theoretical approach?
Comment 5:
The line referenced in the reviewer's response (page 7 line 196) was what I intended to reference with my original comment, and I thank the author(s) for providing this clarification. Furthermore, the changes to the findings provide important information about differences in the author'(s) findings across data sets and clarifies the author'(s) arguments regarding how legislation influences public opinion on LGBTQ+ issues and individuals.
Comment 6:
I appreciate the changes made to the two lines mentioned. I find this measured response accurately reflects the findings from this study yet aligns with the limitations of the data.
Comment 7:
The author has adequately addressed this issue. See response to comment 5.
Comment 8:
I appreciate the author(s) revisions to the manuscript which illustrate the role of assimilation in pathways to LGBTQ+ rights and acceptance.
Comment 9 and 10:
The author has adequately addressed these issue.

Reviewer #1 (Remarks on code availability):

I have reviewed the code for availability, not accuracy.

Reviewer #2 (Remarks to the Author):

Thank you for inviting me to re-review this article. I found the authors had adequately addressed my questions raised earlier. However, the content about MFT that was presented in the previous version was removed but essential for explaining for the characterization of the online posts. I would suggest adding a paragraph to the introduction section about MFT. Other than this, I do not have more questions. Thank you.

Reviewer #4 (Remarks to the Author):

1. Grammar, typos, formatting issues.

Line 180: "congeniality" should be "congenial environment"
Line 310
Before: Other studies have adopt a similar approach for curating online datasets...
After: Other studies have adopted a similar approach for curating online datasets...
Line 331: Missing period after "datasets"

2. Logical reasoning

The argumentation is logical but could benefit from more explicit discussion on the causality and the potential confounding variables affecting public discourse beyond legislative changes.
Alternative explanation for results may be needed.

3. Theoretical aspect

The study could further enhance its theoretical contribution by discussing how these findings fit into broader theories of media effects or cultural change.
The study's focus on moral stereotypes and affective valence in social media discussions adds to the existing literature on the social impacts of legislative changes.

4. Methodological aspect

The authors could further develop their theoretical framework to better explain the complex relationships between social media, public discourse, and social change.
The paper's focus on the "Instagram demographic" feels somewhat narrow, and the authors could consider exploring other social media platforms and their user bases.
The use of interrupted time series analysis is suitable for examining the effects of legislative changes.
The inclusion of control variables and interaction terms in the analysis is methodologically sound.

5. Practical/Industrial implications

The authors could further develop their discussion of the implications of their findings for policymakers, social media companies, and LGBTQ+ advocates.
The paper could benefit from more concrete recommendations for how to address the issues of hate speech, harassment, and marginalization on social media.
The findings have significant implications for policymakers, highlighting the role of legislative changes in reducing public acrimony around marginalized groups.
The study suggests that legislative changes can contribute to easing inter-group tensions and promoting social equality.

Open Access This Peer Review File is licensed under a Creative Commons Attribution 4.0 International License, which permits use, sharing, adaptation, distribution and reproduction in any medium or format, as long as you give appropriate credit to the original author(s) and the source, provide a link to the Creative Commons license, and indicate if changes were made.
In cases where reviewers are anonymous, credit should be given to 'Anonymous Referee' and the source.
The images or other third party material in this Peer Review File are included in the article's Creative Commons license, unless indicated otherwise in a credit line to the material. If material is not included in the article's Creative Commons license and your intended use is not permitted by statutory regulation or exceeds the permitted use, you will need to obtain permission directly from the copyright holder.
To view a copy of this license, visit https://creativecommons.org/licenses/by/4.0/

Response to Reviewer 1

1. **<u>Comment:</u>** The authors engage in an analysis of social media data to assess the impact of legislative changes in four Asian countries on societal attitudes towards LGBTQ+ issues. The authors transparency in discussing their data and methods should be applauded, and they share some potentially illuminating findings about the relationship between social media, legislation, and public discourse. However, there are several issues that should be addressed before this manuscript is ready for publication, as well as a few minor points that need to be resolved.

   <u>Response:</u> We're grateful for the feedback.

2. **<u>Comment:</u>**
   First, the authors claim that the results of this study reflect public opinion on LGBTQ+ topics in India, Taiwan, Thailand, and Singapore. The Instagram and Facebook posts were limited to discourse related to LGBTQ+ related legislation in these nations reported by news outlets in these nations, but I did not see any indication that authors were able to assess the location of users who responded to these news stories.

   <u>Response:</u>
   Clarification on Instagram Posts: We would like to clarify that the Instagram posts included in our study were not limited solely to those related to news outlets. As we say in line 9 of page 2 of the manuscript: *"The questions are addressed through the multi-level modeling of outlet-level data from news organizations in different countries, individual-level data from social media, individual-level data from the Gallup World Poll, and country-level democratic indicators from the Global State of Democracy Indices (GSDI)"*

3. **<u>Comment:</u>**
   Is it possible that the sample includes individuals living in other countries? If so, the authors need to rework their discussion to clarify that this is an assessment of global attitudes rather than reporting findings as "discourse in India [Thailand, Taiwan, or Singapore]".

   <u>Response:</u>
   Geolocation and Dataset Curation: We acknowledge the limitation of not having access to geocoded data through the Instagram API, which would have allowed us to precisely identify the location of users. To mitigate this, we curated our country-specific datasets by searching for posts using a combination of LGBTQ+ hashtags and country-specific keywords. While this method is not flawless, it provides a reasonable approximation of discourse related to specific countries. We will reintroduce a clarification that was present in an earlier version of the manuscript: "Without a way to restrict the Instagram API to geocoded posts, we curated country-specific datasets through searching based

on country keywords." The motivation of using this technique is inspired by other studies that adopt a similar approach for curating datasets of search results relevant to a geographic location, e.g., from curating location-specific Google News results:

Fischer, S., Jaidka, K., & Lelkes, Y. (2020). Auditing local news presence on Google News. *Nature Human Behaviour*, *4*(12), 1236-1244.

Fischer, S., Jaidka, K., & Lelkes, Y. (2022). Reply to: Local news in Google News. *Nature Human Behaviour*, *6*(8), 1045-1047.

Jaidka, K., Fischer, S., Lelkes, Y., & Wang, Y. (2023). News nationalization in a digital age: An examination of how local protests are covered and curated online. *The ANNALS of the American Academy of Political and Social Science*, *707*(1), 189-207.

Framing of Results: We will take additional steps to ensure that this distinction is consistently maintained and clearly communicated throughout the discussion. We are also open to adding this point to the limitations section (online attitudes toward LGBTQ+ people in these four Asian countries are not necessarily posted by individuals from those countries), highlighting it as a potential source of differences between the social media and survey data findings.

In the current manuscript, we had attempted to address this distinction as reflective of LGBTQ+ discourse about [country], rather than directly equating online discourse with public opinion within those countries. For example, in line 1 of the Discussion, we refer to our focus on the "public discourse related to LGBTQ+ topics in different countries."

4. **Comment:** Second, the authors effectively situated their research within moral foundation theory. However, they did not provide any theoretical basis for the main purpose of this study—assessing the impact of legislation on social attitudes towards LGBTQ+ individuals. There are numerous theoretical approaches to this topic and without providing the reader with information about which theoretical frameworks are driving their assessment of the relationship between state-level policies and cultural norms, the study lacks any theoretical significance.

   Response: We agree with the reviewer's assessment and will plan to address this feedback in our revision. The Moral Foundations Theory served as a lens to operationalize measures of public discussions that were relevant for understanding attitudes towards LGBTQ+ people. Yet, as our contribution relates to the role of social institutions such as governments in shifting social identity norms and the acceptance of minority groups, therefore, in our revision, we will involve the Structuration theory in our motivation of the problem and discussion of its findings. The Structuration theory posits

that social practices are shaped by both agency (individual actions) and structures (institutional frameworks) (Giddens, 2014).

When legislative changes, such as the decriminalization of LGBTQ+ identities, occur, they act as a formal recognition and reinforcement of these values at an institutional level. In societies with higher levels of social equality and institutional support for civil liberties, the structures that promote equality and protect individual rights are more robust and ingrained within the social fabric. Structuration theory would therefore lead one to expect that in societies with higher levels of social equality and institutional support for civil liberties, legislative changes around decriminalizing LGBTQ+ minorities may lead to a reconfiguration of moral attitudes.

We hope that incorporating structuration theory in this manner would satisfy the reviewer. Once again, we thank them for the opportunity to clarify the contribution of our work.

5. **Comment:** Third, given the inconsistency in significance observed between Facebook and Instagram around moral stereotypes, individualizing moral values, binding moral values, and toxicity. There are many potential explanations for the inconsistencies found between the two social media platforms (age is likely a contributing factor (active Facebook users tend to be older than Instagram users) but the authors must address this in more detail and include it in the limitations section.

    Response:
    a. Clarification Regarding Facebook Data: We would like to clarify that the Facebook data in our study is sourced from news outlets, rather than individual user posts, and is therefore used to analyze media agendas rather than direct public sentiment. This distinction is important because the Facebook data primarily reflects the content that news organizations choose to highlight, which can differ significantly from the user-generated content analyzed on Instagram. By also considering news content, our aim was to examine whether the legislative changes influence the media's portrayal and framing of LGBTQ+ issues; we find that there were no such changes in the short term.

    b. Addressing Differences Between Instagram and Survey Data: We agree with the concern regarding the inconsistencies between findings, but we decided to focus on the difference between Instagram and survey data as they both reflect individual-level user attitudes rather than news headlines, and are therefore more comparable.

    In fact, our rationale for the use of survey data was to address the lack of representativeness of the Instagram data, as well as to triangulate findings and provide a broader perspective on public attitudes towards LGBTQ+ issues.

In the discussion, while discussing the findings from the secondary (Instagram) dataset, we do say that:
"However, these opinions may be localized to the "Instagram demographic," as triangulating with survey data suggests that the legislative change did not impact the general public's attitudes."

But to examine the differences in findings more systematically, we plan to include a new moderation analysis with the Gallup data, as reported in the Table and Figure below.

	Interaction effects on same-sex couples in the neighborhood	
	Model 1	Model 2
**Main Effect**		
Gender		0.039*
		()
Education		0.014*
		()
Legislative change	-11.523	4.881
	()	()
Days	0.051	0.022 *
	()	()
**Interaction Effects**		
Legislative change x Days	-0.051	0.022 *
**Moderators**		
Legislative change x 18-34	0.172	0.025 ***
		()
Legislative change x 35-49	0.084	0.024 ***
		()
Number of observations: 180; 14,280; *: p< .05; + : p < .10		

Figure 4. Evidence from the secondary dataset: Main Effects and Interaction Effects of the legislative change on the hate speech in public discourse.

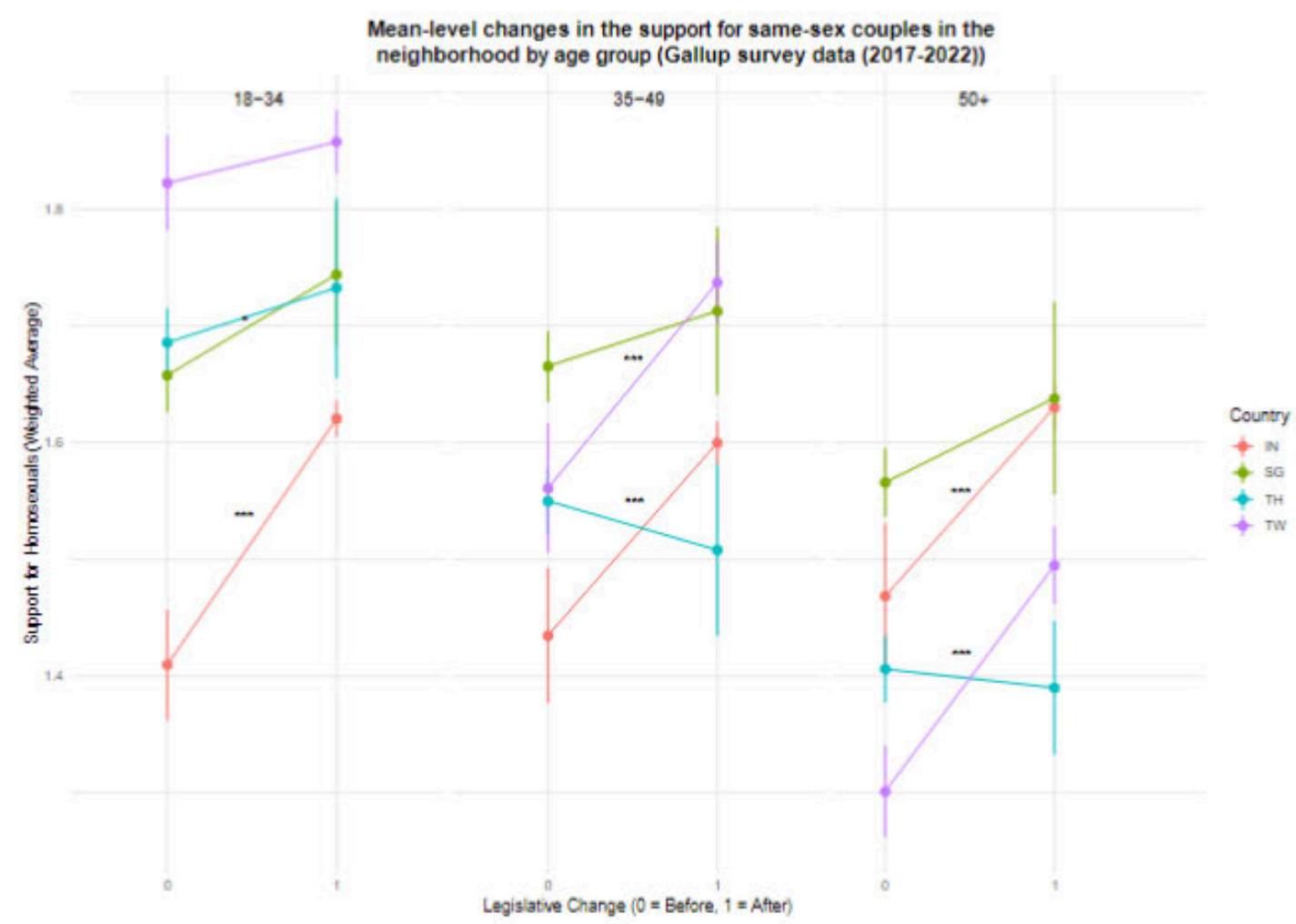

Figure 5. Mean differences in the public attitudes towards same-sex couples in the neighborhood, faceted by age group (bandwidth = 230 days). The error bars report 95% confidence intervals. *($p < 0.05$).

We used the same methods as in Table 1in the original manuscript to assess whether age groups influenced the effect of legislative changes on negative attitudes toward homosexuals in the neighborhood. We divided the age variable into three terciles: 18-34, 35-49, and 50 and above, based on recent reports that indicate at least over two-thirds of Instagram's user base is under 35. Using the 50 and above age group as the reference category, we included an interaction term in the primary effect model to test these differences, and our findings show that age had an interactive effect with legislative change, where, in India and Singapore, younger respondents on the Gallup survey were more likely to consider their neighborhoods safe for same-sex couples after the legislative change, while older respondents in Thailand were less likely to report their neighborhoods as safe for same-sex couples.

c. Inclusion in Limitations Section: We plan to expand our discussion in the limitations section about the representativeness of our datasets and other possible steps that can be taken to mitigate their effects in analyses.

6. **Comment:** Furthermore, the authors also make strong claims that may be overstated given this inconsistency, for example
    - "Our findings support the role played by national institutions in ameliorating the public acrimony around marginalized groups and potentially mending inter-group relations."
    - "We found that after homosexuality was decriminalized in four Asian countries, public discourse was subdued but less affectively polarized and morally charged than before."

    Response: We appreciate the reviewer's observation, and we agree that we will reword these and other statements to be more cautious and acknowledge the limitations and variability in our results. For instance, we plan to change the first example highlighted by the reviewer to: *"Our findings suggest that national institutions can play a role in reducing public acrimony around marginalized groups and may contribute to easing inter-group tensions."*

    As we plan to clarify that the Facebook dataset and Instagram datasets differ based on the authors of the content, we anticipate that claims such as the second one could be reworded as well but would be easier to defend.

7. **Comment:** The three issues mentioned above must be addressed before the study is fit for publication as they represent significant weaknesses in the manuscript as currently written. That stated, I found that the most compelling finding (the third related to the secondary data) was underrepresented and warranted further exploration.

    Response: We agree and we plan to address the issues as outlined above. Additionally, we hope that the proposed exploration of age as a moderator in the shift of public discourse gives the reviewer the depth that they were seeking.

8. **Comment:** The decrease in LGBTQ+ discourse could also be related to the perception that legalization was the only discrimination that LGBTQ+ people face. This has been discussed in relation to marriage equality in the U.S. Queer theorists have argued that these legalization efforts are attempts to assimilate LGBTQ people into heteronormativity, thereby promoting an "acceptable" level of queerness while still perpetuating cultural norms that marginalize LGBTQ+ people.

    Response: We agree with this perspective, and we will try to make this point more emphatically in our revision.
    In the present version of the manuscript, in the Discussion, we say that:

    "These findings suggest that legislative change is not the only factor influencing public discourse related to LGBTQ+ issues. Other factors, such as social norms, political institutions, and cultural attitudes, also play a critical role. These findings match other reports of online and offline LGBTQ+ tolerance from Thailand [33] and Taiwan [55]. For

instance, despite the relative openness of the Taiwanese culture, prior studies have reported how joining LGBTQ+ movements effectively alienates individuals from their family and friends [54]."

In the Conclusion, we say that

"*One would hope that the congeniality online and offline encourages greater LGBTQ+ representation, with positive narratives of authenticity for LGBTQ+ youths to relate to and form their identity [57]. However, beyond legalization, LGBTQ+ people must return to their everyday politics of resistance to question normativity [58]. Additionally, it is essential for the courts and activists to expand the priorities for protecting LGBTQ+ rights, thereby reframing their identities beyond the right to marry [58].*"

We will now plan to add in the reviewers' arguments as well and thank them for educating us.

9. **Comment:** Use same-sex, sexual minority, or LGBTQ instead of homosexual and homosexuality. The term homosexuality is linked to a history of medicalization and stigmatization.

   Response: We are grateful for the reviewer's feedback and will plan to revise the writing accordingly.

10. **Comment:** "region-level" should be capitalized in the first paragraph on page 2

    Response: We will edit this typo.

Response to Reviewer 2

1. **Comment:** Thank you for inviting me to review this article. While the research questions this study sought to explore are highly intriguing and important, I found the article to contain several weaknesses and confusing content. I would suggest thorough clarification and revisions to enhance readability, logical coherence, and the clear presentation of results. My specific comments are provided below.

   Response: We're grateful for the feedback.

2. **Comment:** The question about the moderation effect of democratic indicators on how individuals appraise LGBTQ+ topics seems disconnected from the preceding statements. Could you clarify the importance of identifying these potential moderation effects? Did you aim to examine the robustness of relationships between variables of interest or highlight contextual variabilities in the findings?

Response: We thank the reviewer for the feedback. The moderation effects of democratic indicators are central to our study because they help explain the varying impact of legislative changes on public attitudes toward LGBTQ+ topics. In our revision, we plan to rationalize our approach and discuss our findings in the context of the Structuration Theory, which suggests that individual behaviors and attitudes are shaped by the interaction between agency and institutional and social structures (Giddens, 2014).

As our paper notes, "*social and legislative norms around media and public freedom of expression, as well as social equality, may affect public opinion towards legislative change and the flow of information and ideas in public discourse.*" By including country-level indicators from the Global State of Democracy Indices (GSDI), we aim to explore how these structures influence the impact of legislative changes. As social norms are shaped by interactions between stakeholders within specific political contexts, in our study, democratic indicators represent the conditions that shape public discourse following legislative changes. Understanding these moderation effects helps us identify the contexts in which legislative changes are most likely to shift social attitudes and moral norms.

In doing so, we aimed to highlight contextual variabilities; i.e., how these findings vary across different contexts. Therefore, moderators such as freedom of expression and social equality offer insights into how the impact of legislative changes differs depending on the institutional and social environment.

3. **Comment:** Providing clear rationales and theoretical bases for examining moderation effects would be beneficial.

   Response: We agree with the reviewer. In our revision, we plan to rationalize our approach and discuss our findings in the context of the Structuration Theory, which suggests that individual behaviors and attitudes are shaped by the interaction between agency and institutional and social structures (Giddens, 2014).

4. **Comment:** Additionally, the term "democratic indicators" may be confusing as the moderators mentioned appear to be methodological (sampling, measurement, locations).

   Response: We apologize if this was confusing. Here, we are introducing the “social” indicators of social equality and civil liberaties, such as censorship, freedom of expression, and social equality, as potentially influencing the effect of legislative change on public discussions and attitudes. We will plan to distinguish mentions of moderators from other methodological decisions in the revision.

5. **Comment:** In the results section, the interaction effects tested involve social indicators (such as press freedom, freedom of speech, and social group equities), which are presented as contextual moderators in the methods section, leading to confusion.

   Response: We will plan to be more consistent in the terminology in the revision.

6. **Comment:** The relevance of MFT to this study is not very clear. While it could provide insights into characterizing the moral reasoning underlying public discourse, the study's focus extends beyond this to examine the impact of legal changes on public discourse. It may be beneficial to incorporate other theories to support your main research questions and hypotheses.

   Response: We agree that while MFT provides a valuable base for operationalizing measures of public discussion, the implications of our findings are in understanding the role of social institutions such as governments in shifting social identity norms and the acceptance of minority groups. This forms the basis of Structuration theory, which posits that social practices are shaped by both agency (individual actions) and structures (institutional frameworks) (Giddens, 2014).

   When legislative changes, such as the decriminalization of LGBTQ+ identities, occur, they act as a formal recognition and reinforcement of these values at an institutional level. In societies with higher levels of social equality and institutional support for civil liberties, the structures that promote equality and protect individual rights are more robust and ingrained within the social fabric. Structuration theory would therefore lead one to expect that in societies with higher levels of social equality and institutional support for civil liberties, legislative changes around decriminalizing LGBTQ+ minorities may lead to a reconfiguration of moral attitudes.
   We hope that incorporating structuration theory in this manner would satisfy the reviewer. Once again, we thank them for the opportunity to clarify the contribution of our work.

7. **Comment:** Additionally, the theories are not adequately elaborated for readers. As someone encountering this theory for the first time, I found it challenging to comprehend its meaning, application, and relevance to the study. While one might intuitively expect public discourse on LGBTQ individuals to be categorized as friendly/accepting or exclusionary/hostile, MFT offers an alternative approach to assessing discourse. However, the justification for its appropriateness in this study is not well-established.

   Response: We sincerely apologize for the brevity. In the revision, we will plan to address this feedback through a formal discussion of the expectations of Structuration Theory and how they motivate our study, as well as of the Moral Foundations Theory as a basis for understanding public attitudes.

The Structuration theory posits that social practices are shaped by both agency (individual actions) and structures (institutional frameworks) (Giddens, 2014). When legislative changes, such as the decriminalization of LGBTQ+ identities, occur, they act as a formal recognition and reinforcement of these values at an institutional level. In societies with higher levels of social equality and institutional support for civil liberties, the structures that promote equality and protect individual rights are more robust and ingrained within the social fabric. Structuration theory would therefore lead one to expect that in societies with higher levels of social equality and institutional support for civil liberties, legislative changes around decriminalizing LGBTQ+ minorities may lead to a reconfiguration of moral attitudes.

We hope that incorporating structuration theory in this manner would satisfy the reviewer. Once again, we thank them for the opportunity to clarify the contribution of our work.

8. **Comment:** I believe the introduction section could be condensed to better prepare readers to understand the research questions and hypotheses. Currently, the section covers multiple discussion threads, but the coherence and relevance to the study are not sufficiently strong. For instance, the initial discussion about the design of social media platforms leading to LGBTQ individuals being hypervigilant online and becoming targets of harassment, as well as the mention of ally activism potentially causing victimization, are not directly related to the core questions of the study. The study aims to investigate the impact of societal acceptance and legal changes on discourses about LGBTQ individuals on social media. Similarly, the paragraph on page 2 that starts with 'this study posits that a comprehensive understanding of social media's impact on society...' seems to confuse the study's focus. I recommend that the authors maintain a clear focus on the research objectives throughout the article.

   Response: We thank the reviewer and would be glad to tighten the Introduction section in our revision.

   Comment: 4. Concerning the tertiary dataset, it is debatable to utilize the question of whether respondents perceived their community as safe for LGBT+ individuals as a proxy for public attitudes towards LGBTQ+. This item reflects one's perception of the environment rather than their direct attitudes towards this population. While it may be correlated with related measures, it does not represent public attitudes towards LGBTQ+ individuals.

   Response: The reviewer is right that the questions asked in the Gallup survey relate to whether respondents perceive their community as safe for LGBTQ+ individuals, rather than their personal opinion. In our revision, we will explore prior research that may have been able to establish a link between perceived attitudes and direct attitudes, as in many

studies, perception-based questions such as this are used as a reasonable proxy for public attitudes, because they minimize the risk of biased responses.

As the only cross-national survey that poses such questions year-on-year, Gallup provided us with the unique opportunity to establish public attitudes towards LGBTQ+ people over time through this survey item. We will ensure that the use of this measure and its potential limitations are clearly justified in our revision. We will particularly acknowledge that the survey does not directly capture individual attitude, while offering a way to gauge the general acceptance and implicit attitudes that may relate to individual behavior.

Comment: 5. In the paragraph preceding the limitations section, the authors wrote, "there is an increasing need to recognize LGBTQ+ individuals as a gender minority group." I believe the authors may be conflating sexual minority with gender minority, despite the overlap between them. The relationship between the implications proposed here and the study findings is unclear.

Response: Thank you for bringing this to our attention. We agree that there is a distinction between sexual minorities and gender minorities, and it was not our intention to conflate the two. Our reference to LGBTQ+ individuals as a "gender minority group" was meant to emphasize the intersectionality that often exists within marginalized communities, including both sexual orientation and gender identity. However, we recognize that this phrasing may cause confusion and does not accurately reflect the focus of our study, which pertain to the broader social and institutional treatment of LGBTQ+ individuals as a sexual minority group.

We will revise the wording to clarify this distinction and ensure that the implications are more clearly connected to the study's findings and implications for recognizing the unique challenges faced by LGBTQ+ individuals as a sexual minority, particularly in the context of policy and social attitudes. We hope this change is satisfactory to the reviewer.

Comment: 6. In the limitations the authors acknowledged that this study did not distinguish between stances towards LGBTQ+ people and LGBTQ+ self-expression. Could you elaborate on this point further?

Response: Thank you for your question. In the limitations section, we noted that our study did not distinguish between stances towards LGBTQ+ people and LGBTQ+ self-expression. This limitation means that while the patterns observed provide valuable insights into how LGBTQ+ topics are perceived in the public sphere, the intent behind posting has not been disambiguated. We did not distinguish whether the posts acted to self-identify LGBTQ+ people or were discussions about them. We will ensure that we explain this more clearly in our revision.

Response to Reviewer 3

1. **Comment:** The paper titled, "Decriminalizing Homosexuality and Its Impact on Social Media Discussions in Asia" explores four Asian countries that have decriminalized homosexuality via a study of causal effective legislative change on public attitudes and acceptance towards LGBTQ+ community and intersectional topics thereof. The three multifaceted questions: 1) What are the effects of a legislative change decriminalizing homosexuality, on the (a) news coverage and (b) social media attention to LGBTQ+ topics?; 2) What is the impact of the legal acceptance of homosexual relationships on the (a) negative affect and (b) moral stereotypes in the LGBTQ+ discourse on social media?; and 3)What is the moderation effect of democratic indicators on how individuals appraise LGBTQ+ topics? are centered from a social and psychological lens centered in a legislative perspective. Looking at something from a legislative lens typically means examining it with a focus on how laws, regulations, and policies apply or could apply to the subject at hand. It involves considering the legal implications, constraints, and opportunities that legislation or potential legislation might bring to the issue or topic under discussion. This perspective is particularly important when discussing how laws can shape behaviors, industries, governance structures, and societal norms.

   Response: We're grateful for the feedback.

2. **Comment:** **There were a couple typos/grammatical errors and capitalization issues in the article, such as " region-level" on page 2. Please go over the paper, and add in correction punctuations as well as some sentences are run-on sentences and need to be shorter (more concise).**

   Response: We will be sure to address these in our revision.

3. **Comment:** The datasets constitutes 136,255 multilingual posts from national news outlets in India, Taiwan, Thailand, and Singapore on Facebook between 2016-2023, of which 86.87% were in English, 7.6 million Instagram posts between 2016-2023, of which 74.37% were in English, and 46,200 survey responses from the Gallup World Poll between 2017-2022. Although these datasets are of different distributions, the authors claimed that it enabled a focused understanding and comparison of online vs. offline attitudes towards LGBTQ+ people in four Asian countries namely, India, Taiwan, Thailand, and Singapore.

   Response: We thank the reviewer for highlighting this point. Our datasets indeed have different distributions, both in terms of language and platform usage. Despite these differences, we believe that the combined analysis of these datasets allows for a focused and meaningful comparison of online and offline attitudes towards LGBTQ+

individuals across the four Asian countries studied: India, Taiwan, Thailand, and Singapore. The Facebook dataset, composed mainly of news outlet posts, reflects the media's role in shaping the agenda and reporting on LGBTQ+ issues. The Instagram dataset captures a broader spectrum of public discourse, including both self-expression and public opinion. The Gallup World Poll provides a grounded measure of offline attitudes through a well-established survey methodology.

We used the offline, survey data to triangulate the findings from online data. While we acknowledge the inherent differences in these datasets, the convergence of insights across them strengthens our ability to draw meaningful conclusions about the social and cultural dynamics at play in these countries. We will ensure that the limitations and strengths of using such diverse datasets are clearly addressed in our Discussion.

4. **Comment:** The background does not include or mention much of online discussions, panels, talk page edits, etc. such as Reddit which often contain harmful conversational content i.e., hate speech, death threats and offensive language, especially towards certain demographic groups compared to Instagram's censored or moderated comment section and posting policy, which can most certainly play a crucial role in the results.

   Response: We thank the reviewer for this feedback. We would like to clarify that the point made seems inaccurate; Reddit is a platform that is known for being heavily censored and moderated, whereas Instagram's content moderation, while present, tends to be less strict in comparison. This distinction is important because it influences the type of content and discussions that occur on these platforms, which could indeed impact the results of studies examining online discourse.

   That said, we appreciate the suggestion and would be happy to extend our literature review to include more discussion on the role of platforms like Reddit, talk pages, and other online forums that may harbor harmful conversational content.

5. **Comment:** This study claims to attain a comprehensive understanding of social media's impact on society by considering platforms that have may not display unfiltered comments from individuals who may oppose, criticize, or possess contradictory feelings, beliefs, or motivations towards the LGBTQ+ communities 9Twitter, Reddit), yet posits a NLP approach that focuses on evaluating the effects of legislative changes on media coverage and social media discourse regarding LGBTQ+ topics, examining the dynamics of negative affect and moral stereotypes. The claim, "However, prior work lacks a comprehensive measurement of LGBTQ+ discourse, spanning different modalities and affective dimensions, and the effect of external interventions." is a very large claim with little citation to claim this. For example the following works:

- Experimental Design Considerations for Limiting Psychological Distress presented by Mary Smart (UCSD)
- Decentering Imputation: Fair Learning at the Margins of Demographics presented by Evan Dong (Brown University)
- Iterative Value-Aware Model Learning on the Value Improvement Path presented by Claas Voelcker (University of Toronto)
- Molecular Fingerprints Are a Simple Yet Effective Solution to the Drug–Drug Interaction Problem presented by Yanan Long (University of Chicago)
- From Pedestrian Detection to Crosswalk Estimation: An EM Algorithm, Analysis, and Evaluations on Diverse Datasets presented by Ross Greer (UCSD)
- Detecting Harmful Online Conversational Content towards LGBTQIA+ Individuals. Jamell Dacon, Harry Shomer, Shaylynn Crum-Dacon, Jiliang Tang
- Outed by an Algorithm: A Study on Facebook's Friends Recommendation System for Queer, Trans and Gender-Non-Conforming Users. Jack Chang
- Towards WinoQueer: Developing a Benchmark for Anti-Queer Bias in Large Language Models. Virginia K. Felkner, Ho-Chun Herbert Chang, Eugene Jang, Jonathan May
- Use of a Stylometric Map-Based Corpus for Tracking Individual Variation in Relation to Gender and Sex. Theodore Daniel Manning, Harleigh Niyu, Alejandro Jorge Napolitano Jawerbaum, Patrick Juola
- Overview of STEM Science as Process, Data, Material, and Method Named Entities. Jennifer D'Souza
- CNSC: Czech news article dataset for classification of originating source and its credibility. Matyáš Bohácek
- Tackling Gender Microaggressions in Hindi. Vishakha Agrawal

Response: We thank the reviewer for their feedback and efforts at providing us with a list of relevant references. Of this list of references, we were unable to locate many of them, while others did not seem relevant to the topic of examining social media discussions about LGBTQ+ topics.

We would be happy to include the ones which are relevant to measuring LGBTQ+ discourse and have been peer reviewed. We have observed that these two seem particularly relevant and we would be glad to include them (although the second one has not been included in any published proceedings):

- Felkner, V., Chang, H. C. H., Jang, E., & May, J. (2023, July). WinoQueer: A Community-in-the-Loop Benchmark for Anti-LGBTQ+ Bias in Large Language Models. In *Proceedings of the 61st Annual Meeting of the Association for Computational Linguistics (Volume 1: Long Papers)* (pp. 9126-9140).
- Detecting Harmful Online Conversational Content towards LGBTQIA+ Individuals. Jamell Dacon, Harry Shomer, Shaylynn Crum-Dacon, Jiliang Tang. *Presented at the Queer in AI workshop at NAACL 2022*.

Note that the second paper mentioned above also uses the same approach as ours, i.e., it uses Perspective API to label a Reddit dataset of posts discussing LGBTQIA+ topics, a

subset of which are then manually validated. We will be sure to reference their findings in our revision.

6. **Comment:** How do you define the term "morality" in your study given "Morality refers to the use of language or appeal to moral principles, particularly in discussions involving LGBTQ+ topics. This includes advocating for ethical treatment, human rights, and equality, but it may also condemn LGBTQ+ individuals". Ethical Clarity: It can refer to the ethical or moral dimensions of how something is defined. For example, in discussions about policies or actions (in this article), a moral definition would emphasize the ethical principles and values that guide how something is understood or categorized but from whose perspective, the LGBTQ+ community in the 4 Asian countries,

   Defining Morality: It can also imply defining what is considered moral or ethical in a particular context. This involves specifying the principles, standards, or norms that are deemed morally acceptable or desirable within a society, culture, or philosophical framework.

   In both cases, "moral definition" involves clarifying the ethical implications, considerations, or criteria that shape our understanding and judgment of a given subject or situation.

   Response: We thank the reviewer for this comment. In our Background section, we clarify that our focus is on moral reasoning rather than on broader or ambiguous definitions of morality. Specifically, we examine two dimensions of moral reasoning: individualizing and binding moral values. Individualizing moral values emphasize the compassionate and fair treatment of individuals and include the care/harm and fairness/cheating foundations. For example, care refers to expressions of empathy toward vulnerable individuals, while fairness involves discussions of justice, equality, and reciprocity. On the other hand, binding moral values are concerned with the cohesion and integrity of groups and communities. These include loyalty, which refers to coalition-building, authority, which involves adherence to hierarchical structures, and sanctity, which pertains to maintaining purity and avoiding what is considered unclean.

   The focus on moral reasoning allows us to assess how moral values are invoked in discussions about LGBTQ+ topics across different contexts and platforms. In our revision, we will ensure that our focus on moral reasoning is clear throughout the manuscript.

7. **Comment:** There are NLP models that are trained on toxicity that can also determine the level of toxicity of the english comments and not solely rely on the methods used in this paper namely, pairwise Pearson correlation and ridge regression analysis. This paper does not do a thorough analysis of existing work that has been done and fails to conduct a thorough analysis using state of the art technologies, thus raising several questions

about the findings in terms of correlation, harmful language detection models, specifically for language contains threats, obscene, etc. Also, when taking into consideration "Swear words" it does not account for sarcasm.

Response: We thank the reviewer for their feedback. We would like to clarify a few points. Among the papers that the reviewer shared, only one was directly relevant to our study, and it uses the same method as ours, specifically the Perspective API, to measure toxicity. This is a widely recognized and validated tool for detecting harmful language, including threats and obscenities, in online comments. We also provide its validation on our datasets, in our study.

Comment: 2) Contextual Understanding: Correlations may not capture the nuanced context or intent behind language use in social media posts, as previously mentioned .

Response: Our study does not use Pearson correlation or ridge regression analysis to measure hate speech. Instead, these methods are employed for feature selection, which is a common and effective first step in computational linguistic approaches, including hate speech detection and psycholinguistic analyses of social media text. This method helps identify the most relevant features for further analysis, ensuring that our models are both robust and accurate. Prior studies that have included similar methods for feature selection and to provide linguistic insights into the words with high predictive power include:

- Davidson, T., Warmsley, D., Macy, M., & Weber, I. (2017, May). Automated hate speech detection and the problem of offensive language. In *Proceedings of the international AAAI conference on web and social media* (Vol. 11, No. 1, pp. 512-515).
- Eichstaedt, J. C., Kern, M. L., Yaden, D. B., Schwartz, H. A., Giorgi, S., Park, G., ... & Ungar, L. H. (2021). Closed-and open-vocabulary approaches to text analysis: A review, quantitative comparison, and recommendations. Psychological Methods, 26(4), 398.
- Jaidka, K., Giorgi, S., Schwartz, H. A., Kern, M. L., Ungar, L. H., & Eichstaedt, J. C. (2020). Estimating geographic subjective well-being from Twitter: A comparison of dictionary and data-driven language methods. Proceedings of the national academy of sciences, 117(19), 10165-10171.
- Preoţiuc-Pietro, D., Eichstaedt, J., Park, G., Sap, M., Smith, L., Tobolsky, V., ... & Ungar, L. (2015). The role of personality, age, and gender in tweeting about mental illness. In Proceedings of the 2nd workshop on computational linguistics and clinical psychology: From linguistic signal to clinical reality (pp. 21-30).

However, we acknowledge the importance of considering advanced NLP models, and we will ensure that our approach is thoroughly justified in the manuscript. Additionally, we will cite the relevant references to demonstrate that our methodology aligns with current best practices in the field.

8. **Comment:** For instance, certain words or phrases might be used sarcastically, ironically, or in contexts unrelated to LGBTQ+ topics.

Response: We agree that certain words or phrases might be used sarcastically. Therefore, we conducted a validation on the validation set reported in the paper, after fine-tuning a Google T5 model on a Twitter Sarcasm dataset. We would be able to include this analysis in our appendix, as sarcasm acts as a form of micro-aggression, and possible an indicator or precursor to hate speech.

The Table below shows that about 6-17% of our validation dataset (that evidenced a moral trait) was also labeled positive for sarcasm. We can infer that the proportion of sarcasm in the dataset, and therefore the interactive effect of sarcasm, may be too low-powered to affect our main findings, but sarcasm itself may serve as a useful indicator of hate speech.

	% of Posts with a positive label for sarcasm			
	India	Singapore	Thailand	Taiwan
Posts with moral reasoning	10.91%	17.02%	8.00%	5.88%
Posts without moral reasoning	10.52%	2.98%	3.07%	14.84%
Predictions are based on a T5 model finetuned on the Twitter sarcasm dataset (https://huggingface.co/mrm8488/t5-base-finetuned-sarcasm-twitter)				

Regarding the concern about the potential irrelevance of some text to LGBTQ+ topics, we employed an extensive filtering process to identify and remove posts that may have been hijacking LGBTQ+ keywords for unrelated content. This process was designed to ensure that our dataset remained focused on genuine LGBTQ+ discourse. We conducted a semi-supervised grouping of the most frequent hashtags and performed an additional round of filtering based on the hashtags used in the posts. During this stage, we manually inspected the content and discarded posts that contained co-occurring hashtags irrelevant to LGBTQ+ discourse, such as those related to travel, photography, fitness, and fashion. These posts formed our placebo datasets and are detailed in the supplementary materials.

This approach significantly reduces the likelihood of irrelevant content skewing our analysis. We will acknowledge any remaining issues in our Limitations section to ensure full transparency.

9. **Comment:** Sample Representativeness: The findings are limited by the representativeness of the sample. Instagram users who post about LGBTQ+ topics may not be representative of all social media users or all LGBTQ+ individuals.

Response: We would like to clarify that our focus is not on LGBTQ+ individuals but rather on public attitudes towards LGBTQ+ topics, as we are interested in societal attitudes towards LGBTQ+ people and how they may change after legislative changes are passed.

Second, we fully acknowledge that Instagram users who post about LGBTQ+ topics may not be representative of all social media users or the general population. This is precisely why we triangulate our findings with survey data, which provides a more representative and grounded measure of public opinion. We will be sure to better motivate our decisions in the revision.

10. **Comment:** 4) Language and Culture Variations: The effectiveness of language-based measurements can vary across different languages, cultures, and demographics, which may affect the generalizability of the findings.
5) Data Quality: The quality and reliability of language-based measurements (such as sentiment analysis, keyword analysis, hashtags, etc.) can influence the validity of correlations. Inaccurate or biased measurements can lead to misleading results.

Response: We thank the reviewer for highlighting this. Before running any measurements, we translated all posts to English to circumvent some language-related errors. Yet, in our revision, we will note that there may be a variability in the precision of the methods for different countries.

In a previous response, we have detailed the approach we followed to ensure that the dataset based on keywords was high-quality. We employed an extensive filtering process to identify and remove posts that may have been hijacking LGBTQ+ keywords for unrelated content. This process was designed to ensure that our dataset remained focused on genuine LGBTQ+ discourse.

We have also hand-validated all our measurements on a validation set, as reported in the supplementary materials. We will be sure to emphasize these points in our revised manuscript.

References:

Giddens, A. (2014). Structuration theory: past, present and future. In *Giddens' theory of structuration* (pp. 201-221). Routledge.

Response to Reviewer 1

1. **Comment:** The authors engage in an analysis of social media data to assess the impact of legislative changes in four Asian countries on societal attitudes towards LGBTQ+ issues. The authors transparency in discussing their data and methods should be applauded, and they share some potentially illuminating findings about the relationship between social media, legislation, and public discourse. However, there are several issues that should be addressed before this manuscript is ready for publication, as well as a few minor points that need to be resolved.

   Response: We're grateful for the feedback.

2. **Comment:**
   First, the authors claim that the results of this study reflect public opinion on LGBTQ+ topics in India, Taiwan, Thailand, and Singapore. The Instagram and Facebook posts were limited to discourse related to LGBTQ+ related legislation in these nations reported by news outlets in these nations, but I did not see any indication that authors were able to assess the location of users who responded to these news stories.

   Response:
   Clarification on Instagram Posts: We would like to clarify that the Instagram posts included in our study were not limited solely to those related to news outlets. We have now further clarified this in line 47, Page 2:

   > *"For our analysis, we conducted the multi-level modeling of three datasets: outlet-level data from news organizations in different countries, individual-level data from social media, individual-level data from the Gallup World Poll, and country-level democratic indicators from the Global State of Democracy Indices (GSDI)."*

   The social media data captures a wider spectrum of public discourse, beyond news-related posts alone. However, we acknowledge that our study did not directly verify the geographic location of users who engaged with the posts, as the CrowdTangle API did not allow location-based searches, and we had mentioned it in our study. We had discussed that our findings should be understood as reflecting a global discourse on LGBTQ+ issues, not solely limited to domestic public opinion in India, Taiwan, Thailand, and Singapore. We have now clarified this point. The following paragraphs describe the changes made to the Method section. The next point reports the revisions made to the Discussion section.

   In the Method section, Page 9, line 299, we say that:

   > *The secondary dataset was sourced from Instagram captions via the CrowdTangle API, encompassing public posts about the same four countries during the identical timeframe. Given Instagram's status as Asia's second-most popular platform[66] and its significance for LGBTQ+ individuals' self-expression,[67,68] text analysis of*

Instagram posts is integral to this study. Furthermore, even when the visual content Instagram posts tend to be more abstract, focusing on personal experiences, emotions, or self-portraits[69] the content can be contextualized by the users' self-declared hashtags, indicating a focus on, for instance, the national LGBTQ+ discourse. While this study aims to analyze social media discourse surrounding LGBTQ+ legislation in India, Taiwan, Thailand, and Singapore, the CrowdTangle API did not allow location-based searches of social media posts. Nevertheless, users often rely on hashtags or contextual tags to indicate the focus or relevance of their posts, such as using tags related to specific countries, cities, or events. Accordingly, without a way to restrict the CrowdTangle API to geocoded posts, we curated country-specific datasets through searching based on country keywords, such as India, Taiwan, Thailand, and Singapore, and then further filtering the dataset to retain only those posts which are relevant to LGBTQ+ topics. (...)

We have also clarified why this choice was made. We have added that:

(....) Other studies have adopt a similar approach for curating online datasets relevant to a geographic location by using location-specific keywords in the search query, e.g., for curating location-specific Google News results.[70-71]

As a result of this choice, the data used in this study likely includes contributions from users both within and outside these countries. This means that the discourse captured on social media may reflect a broader, global conversation around LGBTQ issues in each country, rather than being strictly limited to local public opinion.

3. **Comment:**
Is it possible that the sample includes individuals living in other countries? If so, the authors need to rework their discussion to clarify that this is an assessment of global attitudes rather than reporting findings as "discourse in India [Thailand, Taiwan, or Singapore]".

Response:
Geolocation and Dataset Curation: We acknowledge the limitation of not having access to geocoded data through the CrowdTangle API, which would have allowed us to precisely identify the location of users. To mitigate this, we curated our country-specific datasets by searching for posts using a combination of LGBTQ+ hashtags and country-specific keywords, as we have described in our response to the previous section. While this method is not flawless, it provides a reasonable approximation of discourse related to specific countries.

In the current manuscript, we had attempted to address this distinction by ensuring all mentions are *"on LGBTQ+ topics for"* [a country], rather than directly equating online discourse with public opinion within those countries. For example, in line 1 of the

Discussion, we refer to our focus on the "*public discourse related to LGBTQ+ topics in different countries.*"

We have also taken additional steps to ensure that this distinction is consistently maintained and clearly communicated. In the Limitations section, Page 8 line 257, we say that:

> *For the primary and secondary datasets, while our analysis focused on Instagram and Facebook posts and engagement on LGBTQ+ legislation about the four countries studied, the responses we analyzed may include contributions from users outside these regions. The CrowdTangle API does not provide location-specific social media posts; as such, our findings reflect global discourse on local LGBTQ+ issues, which may incorporate perspectives beyond the target countries. This may also be appropriate, given the transnational nature of online discussions on LGBTQ+ topics and rights, and suggests that future research could explore ways to better isolate region-specific public opinion in similar contexts.*

4. **Comment:** Second, the authors effectively situated their research within moral foundation theory. However, they did not provide any theoretical basis for the main purpose of this study—assessing the impact of legislation on social attitudes towards LGBTQ+ individuals. There are numerous theoretical approaches to this topic and without providing the reader with information about which theoretical frameworks are driving their assessment of the relationship between state-level policies and cultural norms, the study lacks any theoretical significance.

   Response: We are grateful for the reviewer's feedback, which has substantially improved the quality of our argument. The Moral Foundations Theory served as a lens to operationalize measures of public discussions that were relevant for understanding attitudes towards LGBTQ+ people. Yet, as our contribution relates to the role of social institutions such as governments in shifting social identity norms and the acceptance of minority groups, therefore, in our revision, we have now involved the Structuration theory in our motivation of the problem and discussion of its findings. The Structuration theory posits that social practices are shaped by both agency (individual actions) and structures (institutional frameworks) (Giddens, 2014).

   In the Introduction, Page 1 line 9, we now say that:

   > *To further explain the dynamics between legislative change and social attitudes, Structuration Theory[10] offers a valuable lens, as it posits how social practices are shaped by both individual agency (the actions of individuals) and structures (institutional frameworks). Legislative changes, such as the decriminalization of LGBTQ+ identities, represent not only shifts in legal structures but also act as catalysts that can reshape social norms and attitudes. When*

governments formally recognize LGBTQ+ rights, they help reinforce societal values of inclusion, which can then influence public discourse, both offline and online. In this way, legislative actions become intertwined with social norms, promoting an environment where individuals feel more empowered to express their identities and engage in discussions about LGBTQ+ issues, particularly in online spaces where freedom of expression may be broader. Therefore, the actions of institutions (like the decriminalization of homosexuality) and individual expressions on social media are interconnected, with both contributing to shifts in public opinion and acceptance of LGBTQ+ individuals. For instance, a lack of representation in government policies may reflect in how LGBTQ+ people are under-represented or vilified in the media and online or portrayed through stereotypes.[11, 12] *This may also explain why individuals were more likely to express their opinions and provide their rationale on LGBTQ+ issues when news emphasizes LGBTQ+ issues.*[13]

On Page 2, line 39, we say that:

Structuration theory would also explain endogenous confounds, if any. In societies with higher levels of social equality and institutional support for civil liberties, the structures that promote equality and protect individual rights are more robust and ingrained within the social fabric. Structuration theory would therefore lead one to expect that in societies with higher levels of social equality and institutional support for civil liberties, legislative changes around decriminalizing LGBTQ+ minorities may lead to a reconfiguration of moral attitudes. In this way, legislative actions become intertwined with social norms, promoting an environment where individuals feel more empowered to express their identities and discuss LGBTQ+ issues, particularly in online spaces where freedom of expression may be broader. We can also establish whether our measurements of social discourse generalize to analyses of public perceptions with other datasets comprising systematic variations in data sampling, the measurement of social media use, and even the mode of data collection. This motivates our final research question: What is the moderation effect of democratic indicators on how individuals appraise LGBTQ+ topics? (RQ3)

In contrast, we have now clarified how Moral Foundations framework offers a way to operationalize societal discourse. On Page 2, in line 31 we say that:

Another research gap lies in understanding how individuals rationalize or reason about LGBTQ+ topics across different contexts and cultures. Prior research has modeled how moral vocabulary shapes discourse,[25] *and moral values influence the framing of news and social events.*[26] *Therefore, examining the moral motivations of LGBTQ+ discussion can provide insights into how they are perceived and rationalized.*[27–29] *LGBTQ+ issues are particularly expected to involve moral intuitions that affect stereotypes and judgments of whether marginalized groups*

> *deserve moral consideration.[30] Given the dual legal and moral nature of decriminalization, our next research question asks: How does the legal acceptance of same-sex relationships impact (a) negative affect and (b) moral stereotypes in LGBTQ+ discourse on social media? (RQ2)*

We return to these ideas in the Conclusion, Page 8 line 219, where we highlight that:

> *Our third finding offers a way to explain the wide variance in findings through examining the interaction effect of structural factors, such as press freedom, freedom of expression, and social group equities, on public responses. The results show that negative affect increased significantly in countries with reduced social equality, such as India. At the same time, it decreased in countries with higher press freedom and greater freedom of expression, such as Taiwan. These findings align with the expectations of the structuration theory as they corroborate the role of social norms, political institutions, and cultural attitudes in explaining societal responses to the legislative change. These findings match other reports of online and offline LGBTQ+ tolerance from Thailand[54] and Taiwan.[55] For instance, despite the relative openness of the Taiwanese culture, prior studies have reported how joining LGBTQ+ movements effectively alienates individuals from their family and friends.[55]*

We hope that incorporating structuration theory and clarifying the role of moral foundations in this manner will satisfy the reviewer. Once again, we thank them for the opportunity to clarify the contribution of our work.

5. **Comment:** Third, given the inconsistency in significance observed between Facebook and Instagram around moral stereotypes, individualizing moral values, binding moral values, and toxicity. There are many potential explanations for the inconsistencies found between the two social media platforms (age is likely a contributing factor (active Facebook users tend to be older than Instagram users) but the authors must address this in more detail and include it in the limitations section.

   Response:
   a. Clarification regarding Facebook data (the primary dataset): We would like to clarify that the Facebook data in our study is sourced from news outlets, rather than individual user posts, and is therefore used to analyze media agendas rather than direct public sentiment. This distinction is important because the Facebook data primarily reflects the content that news organizations choose to highlight, which can differ significantly from the user-generated content analyzed on Instagram. By also considering news content, our aim was to examine whether the legislative changes influence the media's portrayal and framing of LGBTQ+ issues; we find that there were no such changes in the short

term. Furthermore, replicating the analyses with the survey data (tertiary dataset) offers comparable insights to the Instagram data, as they both reflect individual-level opinions and perceptions.

Yet, we take the point that the reviewer may be referring to the lack of attention and engagement with news on the legislative change.

On Page 7, line 196, we say that:

> *However, these shifts may be localized to the "Instagram demographic." First, the findings did not reproduce in the primary dataset in terms of whether the public engaged with the press coverage on LGBTQ+ topics. We speculate that as Facebook users, on average, are older on average than the Instagram user,[47] they may be less favorable towards the legislative change.*

We have also added new analyses that help explain our findings. These are described in the following paragraphs.

b. Addressing the differences in the findings:

Our rationale for the use of survey data was to address the lack of representativeness of the Instagram data, as well as to triangulate findings and provide a broader perspective on public attitudes towards LGBTQ+ issues. To examine the differences in findings more systematically, we have included a new moderation analysis with the Gallup data, as reported in the Table and Figure below. This analysis is reported on Page 6, lines 150-159 and Page 11, lines 361-366.

	Interaction effects on same-sex couples in the neighborhood	
	Model 1	Model 2
**Main Effect**		
Gender		0.039*
		()
Education		0.014*
		()
Legislative change	-11.523	4.881
	()	()
Days	0.051	0.022 *
	()	()
**Interaction Effects**		
Legislative change x Days	-0.051	0.022 *
**Moderators**		
Legislative change x 18-34	0.172	0.025 ***
		()
Legislative change x 35-49	0.084	0.024 ***
		()
Number of observations: 180; 14,280; *: p< .05; + : p < .10		

Figure 4. Evidence from the secondary dataset: Main Effects and Interaction Effects of the legislative change on the hate speech in public discourse.

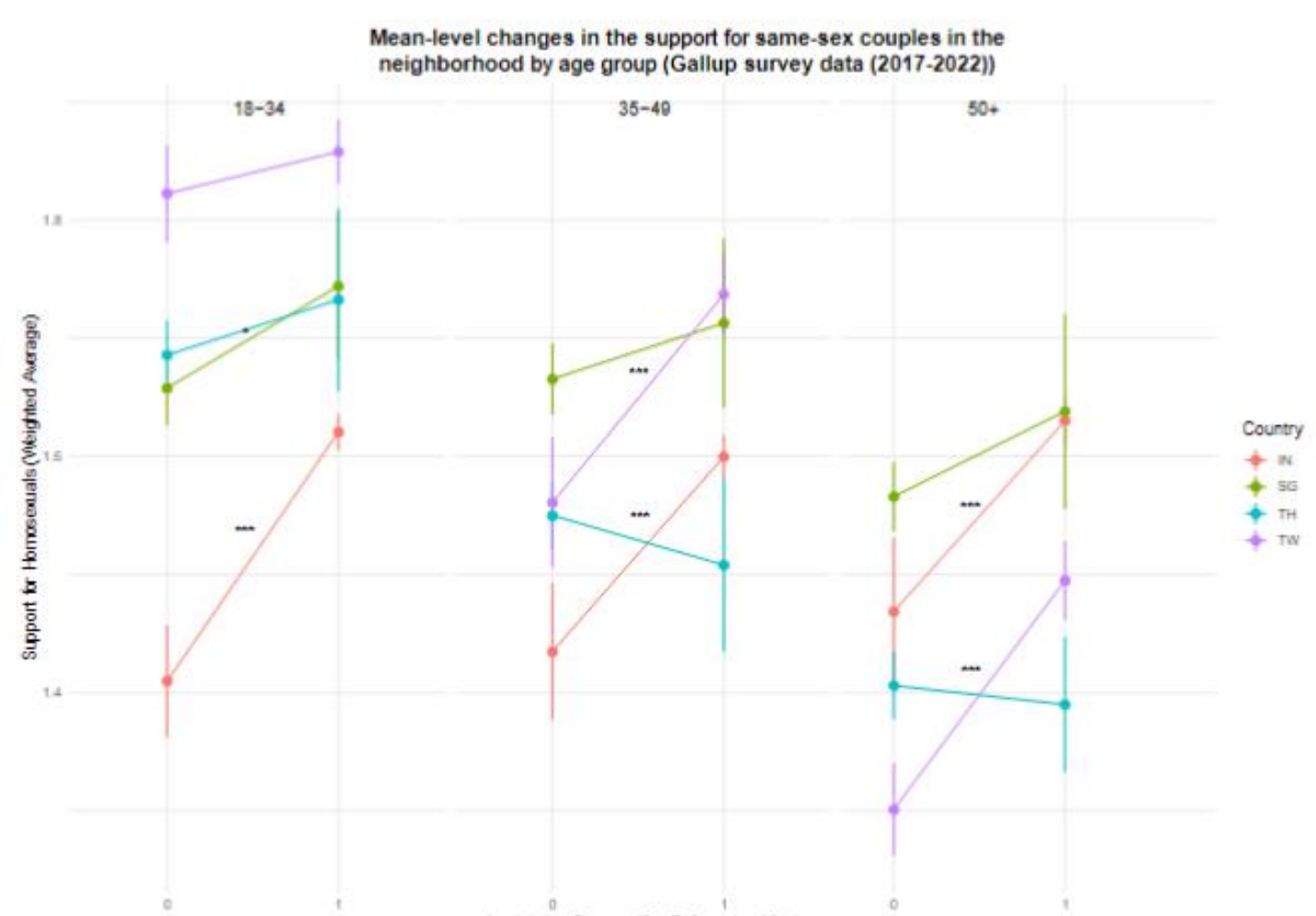

Figure 5. Mean differences in the public attitudes towards same-sex couples in the neighborhood, faceted by age group (bandwidth = 230 days). The error bars report 95% confidence intervals. *($p < 0.05$).

We used the same methods as in Table 1 in the original manuscript to assess whether age groups influenced the effect of legislative changes on negative attitudes toward

homosexuals in the neighborhood. We divided the age variable into three terciles: 18-34, 35-49, and 50 and above, based on recent reports that indicate at least over two-thirds of Instagram's user base is under 35. Using the 50 and above age group as the reference category, we included an interaction term in the primary effect model to test these differences, and our findings show that age had an interactive effect with legislative change, where, in India and Singapore, younger respondents on the Gallup survey were more likely to consider their neighborhoods safe for same-sex couples after the legislative change, while older respondents in Thailand were less likely to report their neighborhoods as safe for same-sex couples.

Then, in this revision, we have synthesized findings from the three datasets to offer a complete picture of the factors contributing to and affecting the variations in the findings we report. On Page 7, line 196, we say that:

> *However, these shifts may be localized to the "Instagram demographic." First, the findings did not reproduce in the primary dataset in terms of whether the public engaged with the press coverage on LGBTQ+ topics. We speculate that as Facebook users, on average, are older on average than the Instagram user,[47] they may be less favorable towards the legislative change. Second, a similar finding from the tertiary dataset suggests no real change in public perception of LGBTQ+ people among the older generation after the legislative change.*

c. Inclusion in Limitations Section: We have overhauled our discussion in the limitations section about the representativeness of our datasets and other possible steps that can be taken to mitigate their effects in analyses. These changes are on Page 8 and 9, lines 256-284.

6. **Comment:** Furthermore, the authors also make strong claims that may be overstated given this inconsistency, for example
    - "Our findings support the role played by national institutions in ameliorating the public acrimony around marginalized groups and potentially mending inter-group relations."
    - "We found that after homosexuality was decriminalized in four Asian countries, public discourse was subdued but less affectively polarized and morally charged than before."

    Response: We appreciate the reviewer's observation. In this revision, we have reworded these and other statements to be more cautious and acknowledge the limitations and variability in our results. For instance, we have changed the examples highlighted by the reviewer to:
    - (Page 8, Line 236): *Our findings suggest that national institutions can play a role in reducing public acrimony around marginalized groups and may contribute to easing inter-group tensions.*

- (Page 8, Line 237): *We found that after same-sex relationships were decriminalized in four Asian countries, the discourse on Instagram was attenuated but less affectively polarized and morally charged than before.*
  We have qualified the second statement as it reflects the findings from the Instagram dataset.

7. **Comment:** The three issues mentioned above must be addressed before the study is fit for publication as they represent significant weaknesses in the manuscript as currently written. That stated, I found that the most compelling finding (the third related to the secondary data) was underrepresented and warranted further exploration.

   Response: We hope that the proposed exploration of age as a moderator in the shift of public discourse has provided insights and a way to tie together our findings. As mentioned in response to the prior comment, we have synthesized findings from the three datasets to offer a complete picture of the factors contributing to and affecting the variations in the findings we report.

   In the Discussion, on Page 7 line 184, we say that:

   > *Our findings suggest that the positive shifts in social discourse around LGBTQ+ topics post-legislative changes may not fully generalize across different data sources, samples, and measurements of perceptions and social attitudes. Instead, these are a factor of their institutional and social environments. The first major finding is from the secondary dataset comprising Instagram posts discussing LGBTQ+ topics for the four countries. We generally observed decreased moral stereotyping of LGBTQ+ topics in the online discourse after the legislative change. (...)*

   We continue on line 196:

   > *However, these shifts may be localized to the "Instagram demographic." First, the findings did not reproduce in the primary dataset in terms of whether the public engaged with the press coverage on LGBTQ+ topics. We speculate that as Facebook users, on average, are older on average than the Instagram user,[47] they may be less favorable towards the legislative change. Second, a similar finding from the tertiary dataset suggests no real change in public perception of LGBTQ+ people among the older generation after the legislative change.*

8. **Comment:** The decrease in LGBTQ+ discourse could also be related to the perception that legalization was the only discrimination that LGBTQ+ people face. This has been discussed in relation to marriage equality in the U.S. Queer theorists have argued that these legalization efforts are attempts to assimilate LGBTQ people into heteronormativity, thereby promoting an "acceptable" level of queerness while still perpetuating cultural norms that marginalize LGBTQ+ people.

Response: We agree enthusiastically with this perspective, and we will try to make this point more emphatically in our revision.
In the Discussion, Page 8 line 222, we say that:

"These findings align with the expectations of the structuration theory as they corroborate the role of social norms, political institutions, and cultural attitudes in explaining societal responses to the legislative change. These findings match other reports of online and offline LGBTQ+ tolerance from Thailand[54] and Taiwan.[55] For instance, despite the relative openness of the Taiwanese culture, prior studies have reported how joining LGBTQ+ movements effectively alienates individuals from their family and friends.[55]"

In the Conclusion, Page 8 line 243, we say that

"While legalization efforts offer a path for LGBTQ+ couples to assimilate into society, legalization is not the only discrimination that LGBTQ+ people face. as they also enforce the heteronormativity that marginalizes LGBTQ+ people. Beyond legalization, LGBTQ+ people must still return to their everyday politics of resistance to question normativity.[59]

Our results show that the treatment of LGBTQ+ people is but a reflection of the gender inequalities that already prevail in society. Therefore, for lasting change, governments may need to look beyond individual laws to create secure environments that protect the freedom of the press, and with equitable treatment for all members of the society. There is also a need to better understand and systematically audit how social and technological infrastructures impact LGBTQ+ people.[18, 53] Policymakers should pay more attention to the impact of legislative change on public discourse related to LGBTQ+ issues and consider ways to create environments that enable social equality. Social media platforms should also take steps to mitigate the spread of hate speech, including through the use of automated content moderation tools and user reporting mechanisms, and to design targeted interventions to promote greater tolerance, acceptance, and freedom of expression for LGBTQ+ individuals."

We thank the reviewer for this pointer and for engaging with our work.

9. **Comment:** Use same-sex, sexual minority, or LGBTQ instead of homosexual and homosexuality. The term homosexuality is linked to a history of medicalization and stigmatization.

   Response: We are grateful for the reviewer's feedback and have revised the writing accordingly, starting with the title.

10. **Comment:** "region-level" should be capitalized in the first paragraph on page 2

    Response: We have edited any typographical errors that remained after a substantive revision of the text.

Response to Reviewer 2

1. **Comment:** Thank you for inviting me to review this article. While the research questions this study sought to explore are highly intriguing and important, I found the article to contain several weaknesses and confusing content. I would suggest thorough clarification and revisions to enhance readability, logical coherence, and the clear presentation of results. My specific comments are provided below.

   Response: We're grateful for the feedback.

2. **Comment:** The question about the moderation effect of democratic indicators on how individuals appraise LGBTQ+ topics seems disconnected from the preceding statements. Could you clarify the importance of identifying these potential moderation effects? Did you aim to examine the robustness of relationships between variables of interest or highlight contextual variabilities in the findings?

   Response: We thank the reviewer for the feedback. We have also revised the preceding text before RQ3 to clarify why studying democratic is central to our study. They help explain the varying impact of legislative changes on public attitudes toward LGBTQ+ topics. As our paper notes on Page 11, line 367, "*social and legislative norms around media and public freedom of expression, as well as social equality, may affect public opinion towards legislative change.*"

   In this revision, we have clarified that the country-level indicators from the Global State of Democracy Indices (GSDI) represent the structural conditions that shape public discourse following legislative changes. Therefore, understanding their moderation effects helps us identify how the impact of legislative changes differs depending on the institutional and social environment. We have explained this through rationalizing our approach in the context of the Structuration Theory, which suggests that individual behaviors and attitudes are shaped by the interaction between agency and institutional and social structures (Giddens, 2014). We provide more details of the related revisions in the following response.

3. **Comment:** Providing clear rationales and theoretical bases for examining moderation effects would be beneficial.

   Response: We agree with the reviewer. In our revision, we have rationalized our approach and discuss our findings in the context of the Structuration Theory, which suggests that individual behaviors and attitudes are shaped by the interaction between agency and institutional and social structures (Giddens, 2014).

On Page 2, line 39, we say that:

> *Structuration theory would also explain endogenous confounds, if any. In societies with higher levels of social equality and institutional support for civil liberties, the structures that promote equality and protect individual rights are more robust and ingrained within the social fabric. Structuration theory would therefore lead one to expect that in societies with higher levels of social equality and institutional support for civil liberties, legislative changes around decriminalizing LGBTQ+ minorities may lead to a reconfiguration of moral attitudes. In this way, examining the interactive effect of democratic indicators can help us identify how the impact of legislative changes differs depending on the institutional and social environment. This motivates our final research question: What is the moderation effect of sociocultural and democratic indicators on how individuals appraise LGBTQ+ topics? (RQ3)*

We return to these ideas in the Conclusion, Page 7 lin 199, where we highlight that:

> *Our third finding offers a way to explain the wide variance in findings through examining the interaction effect of structural factors, such as press freedom, freedom of expression, and social group equities, on public responses. The results show that negative affect increased significantly in countries with reduced social equality, such as India. At the same time, it decreased in countries with higher press freedom and greater freedom of expression, such as Taiwan. These findings align with the expectations of the structuration theory as they corroborate the role of social norms, political institutions, and cultural attitudes in explaining societal responses to the legislative change. These findings match other reports of online and offline LGBTQ+ tolerance from Thailand[54] and Taiwan.[55] For instance, despite the relative openness of the Taiwanese culture, prior studies have reported how joining LGBTQ+ movements effectively alienates individuals from their family and friends.[55]*

4. **Comment:** Additionally, the term "democratic indicators" may be confusing as the moderators mentioned appear to be methodological (sampling, measurement, locations).

   Response: We apologize if this was confusing. Here, we are focusing on the interaction effects of the democratic indicators in a country, such as censorship, freedom of expression, and social equality, as potentially influencing the effect of legislative change on public discussions and attitudes. Therefore, we have clarified any mentions of sampling, measurement and locations and the intent behind them.
   In the Introduction, Page 2 line 54 we say that:

“Triangulating our analyses and validating against survey-level data allows us to understand and compare the online vs. offline attitudes towards LGBTQ+ people in four Asian countries and how these attitudes may shift after legislative changes are instituted. It also helps us establish whether our findings are generalizable beyond the Instagram demographic.[31]”

In the Discussion, on Page 7 line 184, we say that:

Our findings suggest that the positive shifts in social discourse around LGBTQ+ topics post-legislative changes may not fully generalize across different data sources, samples, and measurements of perceptions and social attitudes. Instead, these are a factor of their institutional and social environments.

5. **Comment:** In the results section, the interaction effects tested involve social indicators (such as press freedom, freedom of speech, and social group equities), which are presented as contextual moderators in the methods section, leading to confusion.

   Response: In this revision, we have been more consistent in the terminology. We have retitled the subsection in the Results to “Contributing factors: interaction effects” and the subsection in the Methods to “Contributing factors: moderators.”

6. **Comment:** The relevance of MFT to this study is not very clear. While it could provide insights into characterizing the moral reasoning underlying public discourse, the study's focus extends beyond this to examine the impact of legal changes on public discourse. It may be beneficial to incorporate other theories to support your main research questions and hypotheses. Additionally, the theories are not adequately elaborated for readers. As someone encountering this theory for the first time, I found it challenging to comprehend its meaning, application, and relevance to the study. While one might intuitively expect public discourse on LGBTQ individuals to be categorized as friendly/accepting or exclusionary/hostile, MFT offers an alternative approach to assessing discourse. However, the justification for its appropriateness in this study is not well-established.

   Response: We agree that while MFT provides a valuable base for operationalizing measures of public discussion, the implications of our findings are in understanding the role of social institutions such as governments in shifting social identity norms and the acceptance of minority groups. This forms the basis of Structuration theory, which posits that social practices are shaped by both agency (individual actions) and structures (institutional frameworks) (Giddens, 2014). Therefore, in our revision, we have now involved the Structuration theory in our motivation of the problem and discussion of its findings.

We also apologize for the brevity in our earlier description. In this revision, we have addressed this feedback through a formal discussion of the expectations of Structuration Theory and how they motivate our study, as well as of the Moral Foundations Theory as a basis for understanding public attitudes. In the Discussion section as well, we return to the theory to explain the findings.

In the Introduction, Page 1 line 6, we now say that:

(...) the normative acceptance of same-sex relationships goes beyond legislative action and is intertwined with social and government infrastructural support.

To further explain the dynamics between legislative change and social attitudes, Structuration Theory[10] offers a valuable lens, as it posits how social practices are shaped by both individual agency (the actions of individuals) and structures (institutional frameworks). Legislative changes, such as the decriminalization of LGBTQ+ identities, represent not only shifts in legal structures but also act as catalysts that can reshape social norms and attitudes. When governments formally recognize LGBTQ+ rights, they help reinforce societal values of inclusion, which can then influence public discourse, both offline and online. In this way, legislative actions become intertwined with social norms, promoting an environment where individuals feel more empowered to express their identities and engage in discussions about LGBTQ+ issues, particularly in online spaces where freedom of expression may be broader. Therefore, the actions of institutions (like the decriminalization of homosexuality) and individual expressions on social media are interconnected, with both contributing to shifts in public opinion and acceptance of LGBTQ+ individuals. For instance, a lack of representation in government policies may reflect in how LGBTQ+ people are under-represented or vilified in the media and online or portrayed through stereotypes.[11,12] This may also explain why individuals were more likely to express their opinions and provide their rationale on LGBTQ+ issues when news emphasizes LGBTQ+ issues.[13]

On Page 2, line 37, we say that:

Structuration theory would also explain endogenous confounds, if any. In societies with higher levels of social equality and institutional support for civil liberties, the structures that promote equality and protect individual rights are more robust and ingrained within the social fabric. Structuration theory would therefore lead one to expect that in societies with higher levels of social equality and institutional support for civil liberties, legislative changes around decriminalizing LGBTQ+ minorities may lead to a reconfiguration of moral attitudes. In this way, legislative actions become intertwined with social norms, promoting an environment where individuals feel more empowered to express their identities and discuss LGBTQ+ issues, particularly in online spaces where freedom of expression may be broader. We can also establish whether our measurements of social

discourse generalize to analyses of public perceptions with other datasets comprising systematic variations in data sampling, the measurement of social media use, and even the mode of data collection. This motivates our final research question: What is the moderation effect of democratic indicators on how individuals appraise LGBTQ+ topics? (RQ3)

In contrast, we have now clarified how Moral Foundations framework offers a way to operationalize societal discourse.In our revision, on Page 2 line 31, we say that:

Another research gap lies in understanding how individuals rationalize or reason about LGBTQ+ topics across different contexts and cultures. Prior research has modeled how moral vocabulary shapes discourse,[25] and moral values influence the framing of news and social events.[26] Therefore, examining the moral motivations of LGBTQ+ discussion can provide insights into how they are perceived and rationalized.[27–29] LGBTQ+ issues are particularly expected to involve moral intuitions that affect stereotypes and judgments of whether marginalized groups deserve moral consideration.[30] Given the dual legal and moral nature of decriminalization, our next research question asks: How does the legal acceptance of same-sex relationships impact (a) negative affect and (b) moral stereotypes in LGBTQ+ discourse on social media?

We return to these ideas in the Conclusion, Page 8 line 219, where we highlight that:

Our third finding offers a way to explain the wide variance in findings through examining the interaction effect of structural factors, such as press freedom, freedom of expression, and social group equities, on public responses. The results show that negative affect increased significantly in countries with reduced social equality, such as India. At the same time, it decreased in countries with higher press freedom and greater freedom of expression, such as Taiwan. These findings align with the expectations of the structuration theory as they corroborate the role of social norms, political institutions, and cultural attitudes in explaining societal responses to the legislative change. These findings match other reports of online and offline LGBTQ+ tolerance from Thailand[54] and Taiwan.[55] For instance, despite the relative openness of the Taiwanese culture, prior studies have reported how joining LGBTQ+ movements effectively alienates individuals from their family and friends.[55]

We hope that our response will satisfy the reviewer’s concerns. We thank them for the opportunity to clarify the contribution of our work.

7. **<u>Comment:</u>** I believe the introduction section could be condensed to better prepare readers to understand the research questions and hypotheses. Currently, the section covers multiple discussion threads, but the coherence and relevance to the study are not sufficiently strong. For instance, the initial discussion about the design of social media platforms leading to LGBTQ individuals being hypervigilant online and becoming targets of harassment, as well as the mention of ally activism potentially causing victimization, are not directly related to the core questions of the study. The study aims to investigate the impact of societal acceptance and legal changes on discourses about LGBTQ individuals on social media. Similarly, the paragraph on page 2 that starts with 'this study posits that a comprehensive understanding of social media's impact on society...' seems to confuse the study's focus. I recommend that the authors maintain a clear focus on the research objectives throughout the article.

   <u>Response:</u> We are grateful to the reviewer for helping us streamline our work. We have overhauled the entire Introduction, and would be glad to receive further feedback. As the text comprises two pages, we have not pasted all of it here.

   <u>Comment:</u> 4. Concerning the tertiary dataset, it is debatable to utilize the question of whether respondents perceived their community as safe for LGBT+ individuals as a proxy for public attitudes towards LGBTQ+. This item reflects one's perception of the environment rather than their direct attitudes towards this population. While it may be correlated with related measures, it does not represent public attitudes towards LGBTQ+ individuals.

   <u>Response:</u> We are grateful to the reviewer for pointing this out and helping us to be be more precise in our descriptions. The reviewer is right that the questions asked in the Gallup survey relate to whether respondents perceive their community as safe for LGBTQ+ individuals, rather than their personal opinion. In our revision, we've now clarified this in our original description of the data. In the Method, Page 10, line 318, we say that:

   > *"Daily individual-level perceptions of attitudes towards LGBTQ+ people and rights were collected from the Gallup World Poll."*

   We have also qualified the measure wherever it was elsewhere mentioned.

   On Page 9, line 273, We clarify that:

   > *Although a single-item measure is not ideal, and a third-person perception of environmental safety differs from a direct report of personal attitudes towards LGBTQ+ people, it is the only available daily trend, as the other major cross-national surveys measuring attitudes towards LGBTQ+ people, the Social Acceptance Index of LGBTI people, reported as four-year averages across 178 counties.*[63]

Yet, prior research has also been able to establish a link between perceived attitudes and direct attitudes, as in many studies, perception-based questions such as this are used as a reasonable proxy for public attitudes, because they minimize the risk of biased responses. In the Limitations, we have also added that:

> *Furthermore, a perception-based variable in face-to-face surveys can minimize the risk of biased responses in questions of a sensitive nature. It may also offer a reasonable proxy for personal opinions, as prior work on self- and perceived attitudes of peers towards lesbians and gays suggest that people perceive others' attitudes based on their own predispositions.[64] Analyses based on an online panel of 856 American adults suggested that their media consumption and interpersonal contact predict their attitudes towards same-sex couples, and they presume similar but attenuated effects when they consider the attitudes of their close friends or their peer group.*

Comment: 5. In the paragraph preceding the limitations section, the authors wrote, "there is an increasing need to recognize LGBTQ+ individuals as a gender minority group." I believe the authors may be conflating sexual minority with gender minority, despite the overlap between them. The relationship between the implications proposed here and the study findings is unclear.

Response: Thank you for bringing this to our attention. We agree that there is a distinction between sexual minorities and gender minorities, and it was not our intention to conflate the two. Our reference to LGBTQ+ individuals as a "gender minority group" was meant to emphasize the intersectionality that often exists within marginalized communities, including both sexual orientation and gender identity. However, we recognize that this phrasing may cause confusion and does not accurately reflect the focus of our study, which pertain to the broader social and institutional treatment of LGBTQ+ individuals as a sexual minority group.

In the Conclusion, Page 8 line 249, we have revised the wording to focus more on our recommendation:

There is also a need to better understand and systematically audit how social and technological infrastructures impact LGBTQ+ people.[18,53]

Comment: 6. In the limitations the authors acknowledged that this study did not distinguish between stances towards LGBTQ+ people and LGBTQ+ self-expression. Could you elaborate on this point further?

Response: Thank you for your question. In the limitations section, we noted that our study did not distinguish between stances towards LGBTQ+ people and LGBTQ+ self-expression. This limitation means that while the patterns observed provide valuable

insights into how LGBTQ+ topics are perceived in the public sphere, the intent behind posting has not been disambiguated. We did not distinguish whether the posts acted to self-identify LGBTQ+ people or were discussions about them. We will ensure that we explain this more clearly in our revision. On Page 9, line 263, we have now clarified that:

> *We also recognize that in focusing on LGBTQ+ discourse more broadly, we lose the nuance between stances towards LGBTQ+ people and LGBTQ+ self-expression, which we plan to study in the future to better understand individual cognitive and emotional appraisals after the legislative change, such as through self-disclosures of emotional well-being,[33] stress,[60] locus of control,[61] and loneliness.[62]*

Response to Reviewer 3

1. **<u>Comment:</u>** The paper titled, "Decriminalizing Homosexuality and Its Impact on Social Media Discussions in Asia" explores four Asian countries that have decriminalized homosexuality via a study of causal effective legislative change on public attitudes and acceptance towards LGBTQ+ community and intersectional topics thereof. The three multifaceted questions: 1) What are the effects of a legislative change decriminalizing homosexuality, on the (a) news coverage and (b) social media attention to LGBTQ+ topics?; 2) What is the impact of the legal acceptance of homosexual relationships on the (a) negative affect and (b) moral stereotypes in the LGBTQ+ discourse on social media?; and 3)What is the moderation effect of democratic indicators on how individuals appraise LGBTQ+ topics? are centered from a social and psychological lens centered in a legislative perspective. Looking at something from a legislative lens typically means examining it with a focus on how laws, regulations, and policies apply or could apply to the subject at hand. It involves considering the legal implications, constraints, and opportunities that legislation or potential legislation might bring to the issue or topic under discussion. This perspective is particularly important when discussing how laws can shape behaviors, industries, governance structures, and societal norms.

   <u>Response:</u> We're grateful for the feedback.

2. **<u>Comment:</u>** **There were a couple typos/grammatical errors and capitalization issues in the article, such as " region-level" on page 2. Please go over the paper, and add in correction punctuations as well as some sentences are run-on sentences and need to be shorter (more concise).**

   <u>Response:</u> We have removed any typos that remained.

3. **<u>Comment:</u>** The datasets constitutes 136,255 multilingual posts from national news outlets in India, Taiwan, Thailand, and Singapore on Facebook between 2016-2023, of

which 86.87% were in English, 7.6 million Instagram posts between 2016-2023, of which 74.37% were in English, and 46,200 survey responses from the Gallup World Poll between 2017-2022. Although these datasets are of different distributions, the authors claimed that it enabled a focused understanding and comparison of online vs. offline attitudes towards LGBTQ+ people in four Asian countries namely, India, Taiwan, Thailand, and Singapore.

Response: We thank the reviewer for highlighting this point. Our datasets indeed have different distributions, both in terms of language and platform usage. Despite these differences, we believe that the combined analysis of these datasets allows for a meaningful comparison of online and offline attitudes towards LGBTQ+ individuals across the four Asian countries studied: India, Taiwan, Thailand, and Singapore. The Facebook dataset, composed mainly of news outlet posts, reflects the media's role in shaping the agenda and reporting on LGBTQ+ issues. The Instagram dataset captures a broader spectrum of public discourse, including both self-expression and public opinion. The Gallup World Poll provides a grounded measure of offline attitudes through a well-established survey methodology.

We used the offline, survey data to triangulate the findings from online data. While we acknowledge the inherent differences in these datasets, the convergence of insights across them strengthens our ability to draw meaningful conclusions about the social and cultural dynamics at play in these countries. We have, therefore, clarified how we synthesize results across the different datasets. First, we have better motivated our interaction analysis.

On Page 2, line 37, we say that:

> *Structuration theory would also explain endogenous confounds, if any. In societies with higher levels of social equality and institutional support for civil liberties, the structures that promote equality and protect individual rights are more robust and ingrained within the social fabric. Structuration theory would therefore lead one to expect that in societies with higher levels of social equality and institutional support for civil liberties, legislative changes around decriminalizing LGBTQ+ minorities may lead to a reconfiguration of moral attitudes. In this way, legislative actions become intertwined with social norms, promoting an environment where individuals feel more empowered to express their identities and discuss LGBTQ+ issues, particularly in online spaces where freedom of expression may be broader. We can also establish whether our measurements of social discourse generalize to analyses of public perceptions with other datasets comprising systematic variations in data sampling, the measurement of social media use, and even the mode of data collection. This motivates our final research question: What is the moderation effect of democratic indicators on how individuals appraise LGBTQ+ topics? (RQ3)*

We return to these ideas in the Conclusion, Page 7 lin 199, where we highlight that:

Our third finding offers a way to explain the wide variance in findings through examining the interaction effect of structural factors, such as press freedom, freedom of expression, and social group equities, on public responses. The results show that negative affect increased significantly in countries with reduced social equality, such as India. At the same time, it decreased in countries with higher press freedom and greater freedom of expression, such as Taiwan. These findings align with the expectations of the structuration theory as they corroborate the role of social norms, political institutions, and cultural attitudes in explaining societal responses to the legislative change. These findings match other reports of online and offline LGBTQ+ tolerance from Thailand[51] and Taiwan.[52] For instance, despite the relative openness of the Taiwanese culture, prior studies have reported how joining LGBTQ+ movements effectively alienates individuals from their family and friends.[52]

We hope that our response will satisfy the reviewer's concerns. We thank them for the opportunity to clarify the contribution of our work.

4. **Comment:** The background does not include or mention much of online discussions, panels, talk page edits, etc. such as Reddit which often contain harmful conversational content i.e., hate speech, death threats and offensive language, especially towards certain demographic groups compared to Instagram's censored or moderated comment section and posting policy, which can most certainly play a crucial role in the results.

   Response: We thank the reviewer for this feedback. We would like to clarify on the point being made: Reddit is a platform that is known for being heavily censored and moderated, whereas Instagram's content moderation, while present, tends to be less strict in comparison. This distinction is important because it influences the type of content and discussions that occur on these platforms, which could indeed impact the results of studies examining online discourse.

   That said, we appreciate the suggestion and have discussed how our effects might generalize to other platforms in the Discussion section. On Page 7, line 200, we say that:

   We would also need to consider how the interaction of local cultures with platform-specific dynamics drive the observed shifts in self-disclosure and opinion expression,[48] leading to distinct public discourses across different social media environments. For instance, Reddit, known for its heavily moderated discussions,[49] may display different dynamics. In contrast, Instagram's content moderation, while present, tends to be less strict, allowing for a wider range of expression. This may explain why the shifts in sentiment towards LGBTQ+ topics on Instagram are more visible—Instagram's demographic may not only be more favorable but also more exposed to a wider variety of opinions. Additionally, platforms like Twitter, where

real-time discussions often reflect immediate public reactions, could show entirely different trends depending on how moderation and user demographics interact.

5. **Comment:** This study claims to attain a comprehensive understanding of social media's impact on society by considering platforms that have may not display unfiltered comments from individuals who may oppose, criticize, or possess contradictory feelings, beliefs, or motivations towards the LGBTQ+ communities (Twitter, Reddit), yet posits a NLP approach that focuses on evaluating the effects of legislative changes on media coverage and social media discourse regarding LGBTQ+ topics, examining the dynamics of negative affect and moral stereotypes. The claim, "However, prior work lacks a comprehensive measurement of LGBTQ+ discourse, spanning different modalities and affective dimensions, and the effect of external interventions." is a very large claim with little citation to claim this. For example the following works:
   - Experimental Design Considerations for Limiting Psychological Distress presented by Mary Smart (UCSD)
   - Decentering Imputation: Fair Learning at the Margins of Demographics presented by Evan Dong (Brown University)
   - Iterative Value-Aware Model Learning on the Value Improvement Path presented by Claas Voelcker (University of Toronto)
   - Molecular Fingerprints Are a Simple Yet Effective Solution to the Drug–Drug Interaction Problem presented by Yanan Long (University of Chicago)
   - From Pedestrian Detection to Crosswalk Estimation: An EM Algorithm, Analysis, and Evaluations on Diverse Datasets presented by Ross Greer (UCSD)
   - Detecting Harmful Online Conversational Content towards LGBTQIA+ Individuals. Jamell Dacon, Harry Shomer, Shaylynn Crum-Dacon, Jiliang Tang
   - Outed by an Algorithm: A Study on Facebook's Friends Recommendation System for Queer, Trans and Gender-Non-Conforming Users. Jack Chang
   - Towards WinoQueer: Developing a Benchmark for Anti-Queer Bias in Large Language Models. Virginia K. Felkner, Ho-Chun Herbert Chang, Eugene Jang, Jonathan May
   - Use of a Stylometric Map-Based Corpus for Tracking Individual Variation in Relation to Gender and Sex. Theodore Daniel Manning, Harleigh Niyu, Alejandro Jorge Napolitano Jawerbaum, Patrick Juola
   - Overview of STEM Science as Process, Data, Material, and Method Named Entities. Jennifer D'Souza
   - CNSC: Czech news article dataset for classification of originating source and its credibility. Matyáš Bohácek
   - Tackling Gender Microaggressions in Hindi. Vishakha Agrawal

   Response: We thank the reviewer for their feedback and efforts at providing us with a list of relevant references. We agree that our original claim could be worded differently and have now removed this line, as it is not our research contribution.

In this revision, we have also evaluated another approach for toxicity measurement developed by Dacon et al. (2021). However, upon validating the labels with human annotations, we found that the Perspective API was more accurate, perhaps because of the cross-national context and the short text format of Instagram captions. Therefore, we have retained the average Perspective API scores as our main measure of toxicity.

However, of this list of references, we were unable to locate many of them, while others did not seem relevant to the topic of examining social media discussions about LGBTQ+ topics. Please see a point-by-point examination of the list of references below:

**Reviewer 3's suggested reference**	**Our response**
- Iterative Value-Aware Model Learning on the Value Improvement Path presented by Claas Voelcker (University of Toronto)	We could not locate this article online.
- Experimental Design Considerations for Limiting Psychological Distress presented by Mary Smart (UCSD)	Link - https://medium.com/@mary_ann_e/experimental-design-considerations-for-limiting-psychological-distress-d70d8f957007<br><br>We are grateful to the pointer and we believe the idea is useful to consider other psychological outcomes worthy of examination in a possible extension to the current research design. Therefore, we have included prominent research on the measurement of psychological distress and depression on social media in our Limitation section. On page 8, line 263, we say that:<br><br>*We also recognize that in focusing on LGBTQ+ discourse more broadly, we lose the nuance between stances towards LGBTQ+ people and LGBTQ+ self-expression, which we plan to study in the future to better understand individual cognitive and emotional appraisals after the legislative change, such as through self-disclosures of emotional well-being,[40] stress,[59] locus of control,[60] and loneliness.[61]*
- Decentering Imputation: Fair Learning at the Margins of Demographics presented by Evan Dong (Brown University)	Link: https://cs.brown.edu/people/ccousins/projects/adversarialfairness/decenteringQAI22.pdf<br><br>• This article does not appear to be relevant to the present study, and it is about data imputation. As we do not propose any machine learning for demographics from our dataset, we have not cited it in our revision.
- Molecular Fingerprints Are a Simple Yet Effective Solution to the Drug–Drug Interaction Problem presented by Yanan Long (University of Chicago)	Link: https://icml-compbio.github.io/2022/papers/WCBICML2022_paper_72.pdf<br>• This article does not appear to be relevant to the present study, and it is about pharmaceutical drugs from a

	computational biology workshop.
- From Pedestrian Detection to Crosswalk Estimation: An EM Algorithm, Analysis, and Evaluations on Diverse Datasets presented by Ross Greer (UCSD)	Link - https://arxiv.org/abs/2205.12579<br>• This article does not appear to be relevant to the present study, and it is about pedestrian crosswalks.
- Overview of STEM Science as Process, Data, Material, and Method Named Entities. Jennifer D'Souza	Link: https://www.mdpi.com/2673-9585/2/4/42<br>• This article does not appear to be relevant to the present study, and it is about STEM science.
- Outed by an Algorithm: A Study on Facebook's Friends Recommendation System for Queer, Trans and Gender-Non-Conforming Users. Jack Chang	We could not locate this article online. It was listed in the program of the Queer in AI workshop at NAACL 2022 as a panel presentation.<br><br>While the topic does not appear to be relevant for the current study, we take the point to discuss the algorithmic implications of engaging with LGBTQ+ content on social media, and have cited the following paper instead:<br><br>Cho, A. (2018). Default publicness: Queer youth of color, social media, and being outed by the machine. *New Media & Society*, *20*(9), 3183-3200.
- Detecting Harmful Online Conversational Content towards LGBTQIA+ Individuals. Jamell Dacon, Harry Shomer, Shaylynn Crum-Dacon, Jiliang Tang	We have now cited and evaluated this approach in our revision.
- Use of a Stylometric Map-Based Corpus for Tracking Individual Variation in Relation to Gender and Sex. Theodore Daniel Manning, Harleigh Niyu, Alejandro Jorge Napolitano Jawerbaum, Patrick Juola	We could not locate this article online. It was listed in the program of the Queer in AI workshop at NAACL 2022 as a panel presentation.<br><br>While the topic does not appear to be relevant for the current study, we take the point to discuss within-sample differences in how LGBTQ+ keywords and symbols may differ in the social media posts of different countries. On Page 7, line 200, we say that:<br>*We would also need to consider how the interaction of local cultures with platform-specific dynamics drive the observed shifts in self-disclosure and opinion expression,[48] leading to distinct public discourses across different social media environments.*<br><br>On Page 8, line 267, we say that:<br>*We offset our reliance on the accuracy of the translation and automatic classification technologies through validating the automatic scores with human evaluation, validating our measurements with new approaches, and ensuring that our results hold for different time windows. Our analytical design also focuses on the shifts in their measurements, leaving us to focus on the effect of the intervention keeping other things unchanged.*

	In the supplementary materials, we have also provided all the keywords that were used in the construction of our dataset as well as the contribution of the major accounts, barring some details on individual-level accounts for privacy reasons.
- Tackling Gender Microaggressions in Hindi. Vishakha Agrawal	We could not locate this article online. It was a presentation without a paper in the Queer in AI workshop at NAACL 2022.<br><br>Although were unable to find this article; however, we applied the idea to evaluate a context-specific hate speech detection approach in this revision.
- Towards WinoQueer: Developing a Benchmark for Anti-Queer Bias in Large Language Models. Virginia K. Felkner, Ho-Chun Herbert Chang, Eugene Jang, Jonathan May	We appreciate the pointer to this study. While we could not locate this particular paper online, we found its follow-up work, and have used it to corroborate our exploratory analysis:<br><br>Felkner, V., Chang, H. C. H., Jang, E., & May, J. (2023, July). WinoQueer: A Community-in-the-Loop Benchmark for Anti-LGBTQ+ Bias in Large Language Models. In *Proceedings of the 61st Annual Meeting of the Association for Computational Linguistics* (Volume 1: Long Papers) (pp. 9126-9140).<br><br>On Page 3, line 88, we say that:<br><br>*Figure 1b depicts a language matrix for the words with the highest positive correlation with the toxicity and moral stereotype measurements in the form of word clouds. The toxicity measure from the Perspective API follows a supervised learning approach.[33] Unexpectedly, its word cloud includes LGBTQ+ topics rather than traditional swear words, suggesting that posts related to LGBTQ+ discourse are more likely to be flagged as toxic, or that the machine learning models may be implicitly biased in recognizing LGBTQ+ topics as predictors of toxicity. For instance, the more the highly frequent word "queer" was mentioned, the more likely the post was to obtain a higher toxicity rating. Our findings corroborate recent work examining the bias in hate speech detection with the more sophisticated large language models.[41] The moral stereotypes measure is a psycholinguistic dictionary using a frequency-based approach, and the indicator words reflect those originally curated by psychologists.36 In the word cloud for moral stereotypes, we can observe that the word "pride" had a strong positive association with higher scores on the moral stereotypes measure.*

We have added a new subsection to our supplementary materials to discuss additional measures of valence.

In the supplementary materials, Pg 5 line 129, we say that:

Additional measures of valence:
We examined other measures of valence, yet they often performed poorly on labeling our dataset. Our efforts are detailed below:

- Alternative measures of moral valence: Following an LSTM-based approach for the measurement of moral valence,[18] we trained LSTM classifiers on their annotated dataset of social media posts. The classifiers detect moral valence in the primary and secondary datasets on a scale of 0 to 1 each for care, loyalty, and betrayal. The resultant models had an average training loss of 0.142. In a ten-fold cross-validation setup, the models had an average accuracy of 0.934. However, the low precision and recall scores on the in-domain data, reported in Table 6, deterred us from applying it to our dataset to generate labels of moral valence.
- Alternative measures of toxicity: We finetuned a T5 classifier on an additional dataset comprising 9,930 comments[19] that focuses on the queerness dimension in comments involving LGBTQIA+ individuals. While the dataset originally contains six labels of toxicity, we adapted it to build a binary classifier, distinguishing between toxic and non-toxic content. We finetuned the model for 10 epochs using a learning rate of 2e-5, weight decay of 0.01, and batch size of 64. The resultant model had a training loss of 0.361, and in a ten-fold cross-validation setup, it had an average accuracy of 0.826. This classifier labeled a large proportion of the captions (97%) as positive. Yet its low accuracy of 0.35, as reported in Table 6, meant that it was not suitable to apply in our analysis.

6. **<u>Comment:</u>** How do you define the term "morality" in your study given "Morality refers to the use of language or appeal to moral principles, particularly in discussions involving LGBTQ+ topics. This includes advocating for ethical treatment, human rights, and equality, but it may also condemn LGBTQ+ individuals". Ethical Clarity: It can refer to the ethical or moral dimensions of how something is defined. For example, in discussions about policies or actions (in this article), a moral definition would emphasize the ethical principles and values that guide how something is understood or categorized but from whose perspective, the LGBTQ+ community in the 4 Asian countries,

   Defining Morality: It can also imply defining what is considered moral or ethical in a particular context. This involves specifying the principles, standards, or norms that are deemed morally acceptable or desirable within a society, culture, or philosophical framework.

   In both cases, "moral definition" involves clarifying the ethical implications, considerations, or criteria that shape our understanding and judgment of a given subject or situation.

   <u>Response:</u> We thank the reviewer for their insightful comment regarding the definition of "morality" in our study. In our manuscript, we distinguish between broad definitions of morality and our specific focus on moral reasoning, particularly as it relates to LGBTQ+ discourse. We apply the moral foundations framework to examine how two key dimensions of moral reasoning—individualizing and binding moral values—shape discussions about LGBTQ+ issues.

Individualizing moral values, such as care/harm and fairness/cheating, emphasize empathy and justice at the individual level, while binding moral values, such as loyalty, authority, and sanctity, focus on maintaining group cohesion and societal norms. These moral dimensions help us understand how people rationalize or reason about LGBTQ+ issues across various contexts, including both supportive and oppositional viewpoints.

In our revision, on Page 2 line 31, we say that:

Another research gap lies in understanding how individuals rationalize or reason about LGBTQ+ topics across different contexts and cultures. Prior research has modeled how moral vocabulary shapes discourse,[25] and moral values influence the framing of news and social events.[26] Therefore, examining the moral motivations of LGBTQ+ discussion can provide insights into how they are perceived and rationalized.[27–29] LGBTQ+ issues are particularly expected to involve moral intuitions that affect stereotypes and judgments of whether marginalized groups deserve moral consideration.[30] Given the dual legal and moral nature of decriminalization, our next research question asks: How does the legal acceptance of same-sex relationships impact (a) negative affect and (b) moral stereotypes in LGBTQ+ discourse on social media?

7. **Comment:** There are NLP models that are trained on toxicity that can also determine the level of toxicity of the english comments and not solely rely on the methods used in this paper namely, pairwise Pearson correlation and ridge regression analysis. This paper does not do a thorough analysis of existing work that has been done and fails to conduct a thorough analysis using state of the art technologies, thus raising several questions about the findings in terms of correlation, harmful language detection models, specifically for language contains threats, obscene, etc. Also, when taking into consideration "Swear words" it does not account for sarcasm.

   Response: We thank the reviewer for their feedback. We would like to clarify a few points. Among the papers that the reviewer shared, only one was directly relevant to our study, and it uses the same method as ours, specifically the Perspective API, to examine toxicity. This is a widely recognized and validated tool for detecting harmful language, including threats and obscenities, in online comments. We also provide its validation on our datasets, in our study, and discuss the list of references provided by the reviewer in a following point.

   On the other hand, we recognize that certain words or phrases might be used sarcastically, which can impact how language is interpreted, particularly as sarcasm can serve as a form of micro-aggression and potentially as a precursor to hate speech. To account for this, we fine-tuned a T5 binary classifier on a Sarcasm Dataset[1] comprising

[1] https://huggingface.co/datasets/nikesh66/Sarcasm-dataset

5,000 tweets. Out of the total tweets in the training set, 4,000 were labeled as non-sarcastic, and 1,000 as sarcastic. We finetuned for 10 epochs with a constant learning rate of 2e-5, weight decay of 0.01, and batch size of 64. Ultimately, the trained model had a training loss of 0.361 and an accuracy on a held out validation set of 0.826.

Once we generated the labels for our dataset, we observed that it labeled 99% of the dataset as positive for sarcasm. Upon validating the labels with human annotations, where we considered "insults" to be the closest interpretation of sarcasm, we found the classifier had a low accuracy on our validation dataset, at 0.30. The Precision was 0.406 while the Recall was 0.650.

The low performance may have been because of the cross-national contexts of the captions as compared to the homogeneous training set, which was curated from American social media posts. We observed that similar issues occurred with other finetuned models based on data from a different culture, such as when we attempted to finetune an LSTM classifier with data annotated with moral foundations dimensions, and when finetuning a T5 classifier with hate speech data specific to LGBTQ+ communities. Yet, we agree that it would be interesting to examine sarcasm and other kinds of microaggressions in future work, mainly to understand whether our findings about the shift in discourse also occur for these more nuanced measures. We have addressed this idea in our Limitations section.

Comment: 2) Contextual Understanding: Correlations may not capture the nuanced context or intent behind language use in social media posts, as previously mentioned.

Response: Our study does not use Pearson correlation or ridge regression analysis to measure hate speech. Instead, these methods are employed for feature selection, which is a common and effective first step in computational linguistic approaches, including hate speech detection and psycholinguistic analyses of social media text. This method helps identify the most relevant features for further analysis, ensuring that our models are both robust and accurate.

In this revision, we have noted the reviewer's feedback and provided citations to other prior studies that have included similar methods for feature selection, and who also provide linguistic insights into the words with high predictive power. We have cited the following papers:

- Davidson, T., Warmsley, D., Macy, M., & Weber, I. (2017, May). Automated hate speech detection and the problem of offensive language. In *Proceedings of the international AAAI conference on web and social media* (Vol. 11, No. 1, pp. 512-515).
- Eichstaedt, J. C., Kern, M. L., Yaden, D. B., Schwartz, H. A., Giorgi, S., Park, G., ... & Ungar, L. H. (2021). Closed-and open-vocabulary approaches to text analysis: A review, quantitative comparison, and recommendations. Psychological Methods, 26(4), 398.
- Jaidka, K., Giorgi, S., Schwartz, H. A., Kern, M. L., Ungar, L. H., & Eichstaedt, J. C. (2020). Estimating geographic subjective well-being from Twitter: A comparison of dictionary and data-driven language methods. Proceedings of the national academy of sciences, 117(19), 10165-10171.
- Preoţiuc-Pietro, D., Eichstaedt, J., Park, G., Sap, M., Smith, L., Tobolsky, V., ... & Ungar, L. (2015). The role of personality, age, and gender in tweeting about mental illness. In Proceedings of the 2nd

workshop on computational linguistics and clinical psychology: From linguistic signal to clinical reality (pp. 21-30).

On Page 2, line 70, we say that:

First, an exploratory analysis was conducted to empirically select measures for the LGBTQ+ discourse in the primary and secondary datasets. The purpose of this analysis was to identify the most relevant language-based features driving the discourse, which helps ensure that our models are robust and capable of capturing key dynamics in LGBTQ+ discussions. Our feature selection process, which employs Pearson correlation and ridge regression analyses—common and effective approaches in computational linguistics, including hate speech detection and psycholinguistic analysis.[32-35]

8. **Comment:** For instance, certain words or phrases might be used sarcastically, ironically, or in contexts unrelated to LGBTQ+ topics.

   Response: We agree that certain words or phrases might be used sarcastically. Therefore, we conducted a validation on the validation set reported in the paper, after fine-tuning a Google T5 model on a Twitter Sarcasm dataset, and this has been described in response to Comment 6.

   Regarding the concern about the potential irrelevance of some text to LGBTQ+ topics, we employed an extensive filtering process to identify and remove posts that may have been hijacking LGBTQ+ keywords for unrelated content. This process was designed to ensure that our dataset remained focused on genuine LGBTQ+ discourse. We conducted a semi-supervised grouping of the most frequent hashtags and performed an additional round of filtering based on the hashtags used in the posts. During this stage, we manually inspected the content and discarded posts that contained co-occurring hashtags irrelevant to LGBTQ+ discourse, such as those related to travel, photography, fitness, and fashion. These posts formed our placebo datasets and are detailed in the supplementary materials.

   This approach significantly reduces the likelihood of irrelevant content skewing our analysis. We will acknowledge any remaining issues in our Limitations section to ensure full transparency.

9. **Comment:** Sample Representativeness: The findings are limited by the representativeness of the sample. Instagram users who post about LGBTQ+ topics may not be representative of all social media users or all LGBTQ+ individuals.

   Response: We would like to clarify that our focus is not on LGBTQ+ individuals but rather on public attitudes towards LGBTQ+ topics, as we are interested in societal attitudes towards LGBTQ+ people and how they may change after legislative changes are passed.

We also fully acknowledge that Instagram users who post about LGBTQ+ topics may not be representative of all social media users or the general population. This is precisely why we triangulate our findings with survey data, which provides a more representative and grounded measure of public opinion.

In the Discussion, on Page 7 line 184, we clarify the purpose of having multiple data sources by saying that:

> *Our findings suggest that the positive shifts in social discourse around LGBTQ+ topics post-legislative changes may not fully generalize across different data sources, samples, and measurements of perceptions and social attitudes. Instead, these are a factor of their institutional and social environments.*

We continue on line 196:

> *However, these shifts may be localized to the "Instagram demographic." First, the findings did not reproduce in the primary dataset in terms of whether the public engaged with the press coverage on LGBTQ+ topics. We speculate that as Facebook users, on average, are older on average than the Instagram user,[46] they may be less favorable towards the legislative change. Second, a similar finding from the tertiary dataset suggests no real change in public perception of LGBTQ+ people among the older generation after the legislative change.*

10. **Comment:** 4) Language and Culture Variations: The effectiveness of language-based measurements can vary across different languages, cultures, and demographics, which may affect the generalizability of the findings.
5) Data Quality: The quality and reliability of language-based measurements (such as sentiment analysis, keyword analysis, hashtags, etc.) can influence the validity of correlations. Inaccurate or biased measurements can lead to misleading results.

Response: We thank the reviewer for highlighting this. Before running any measurements, we translated all posts to English to circumvent some language-related errors. Yet, in our revision, we will note that there may be a variability in the precision of the methods for different countries.

In a previous response, we have detailed the approach we followed to ensure that the dataset based on keywords was high-quality. We employed an extensive filtering process to identify and remove posts that may have been hijacking LGBTQ+ keywords for unrelated content. This process was designed to ensure that our dataset remained focused on genuine LGBTQ+ discourse. On Page 8, line 267, we say that:

> *We offset our reliance on the accuracy of the translation and automatic classification technologies through validating the automatic scores with human evaluation, validating our measurements with new approaches, and ensuring that our results hold for different time windows. Our analytical design also focuses on the shifts in their measurements, leaving us to focus on the effect of the intervention keeping other things unchanged.*

References:

Dacon, J., Shomer, H., Crum-Dacon, S., & Tang, J. (2022). Detecting Harmful Online Conversational Content towards LGBTQIA+ Individuals. *arXiv preprint arXiv:2207.10032*.

Giddens, A. (2014). Structuration theory: past, present and future. In *Giddens' theory of structuration* (pp. 201-221). Routledge.
